# Supplementary material for: Molecular basis of XPF-ERCC1 targeting to SLX4-dependent DNA repair pathways
Source: Nat Commun. 2025 Dec 16;17:522. doi: 10.1038/s41467-025-67216-3 (PMC12804970; doi:10.1038/s41467-025-67216-3)
Supplement: Supplementary file 1 — Supplementary Information [file 41467_2025_67216_MOESM1_ESM.pdf]

Supplementary Information

Molecular basis of XPF-ERCC1 targeting to SLX4-dependent DNA repair pathways

Junjie Feng, Peter R. Martin, Szymon Kowalski, Maxime Lecot, Nora B. Cronin, Teige Matthews-Palmer, Wojciech Niedzwiedz, Basil J. Greber*

* correspondence to: basil.greber@icr.ac.uk (B.J.G.)

This file includes:
Supplementary Figures 1-17
Supplementary Tables 1-2

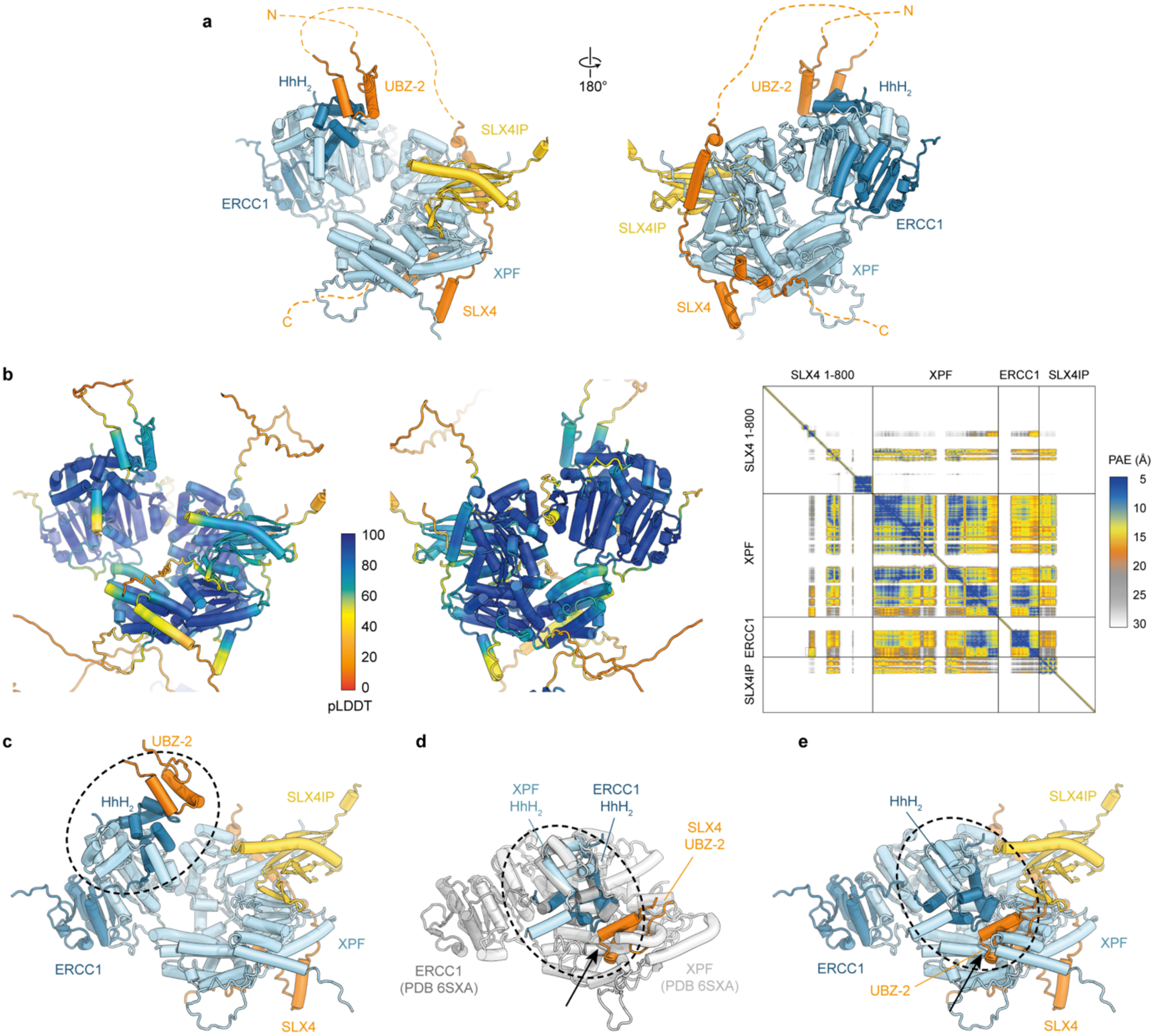

Supplementary Figure 1 | AlphaFold2 prediction of the structure of the XPF-ERCC1-SLX4IP-SLX4 complex. (**a**) AlphaFold2 prediction of the structure of the XPF-ERCC1-SLX4IP-SLX4 complex coloured by subunit (XPF cyan, ERCC1 blue, SLX4IP yellow, SLX4 orange; only SLX4 residues 1-800 were used for this prediction). Long unstructured segments with pLDDT score < 40 are truncated and are represented by dashed lines. (**b**) Structure prediction in panel a coloured by pLDDT score (left) and corresponding predicted aligned error (PAE) matrix (right). (**c**-**e**) Analysis of functional implications of the presence of the SLX4 UBZ-2 domain at the ERCC1 HhH_2 domain. The UBZ-2-HhH_2 module is highlighted in c. The same module superposed on the auto-inhibited resting state of the enzyme (PDB ID 6SXA) results in a clash between the SLX4 UBZ-2 domain and the XPF helical domain (d, indicated by an arrow). The clash is also present when using the AlphaFold2 prediction rather than the experimental structure to model the auto-inhibited complex in the presence of SLX4 (e).

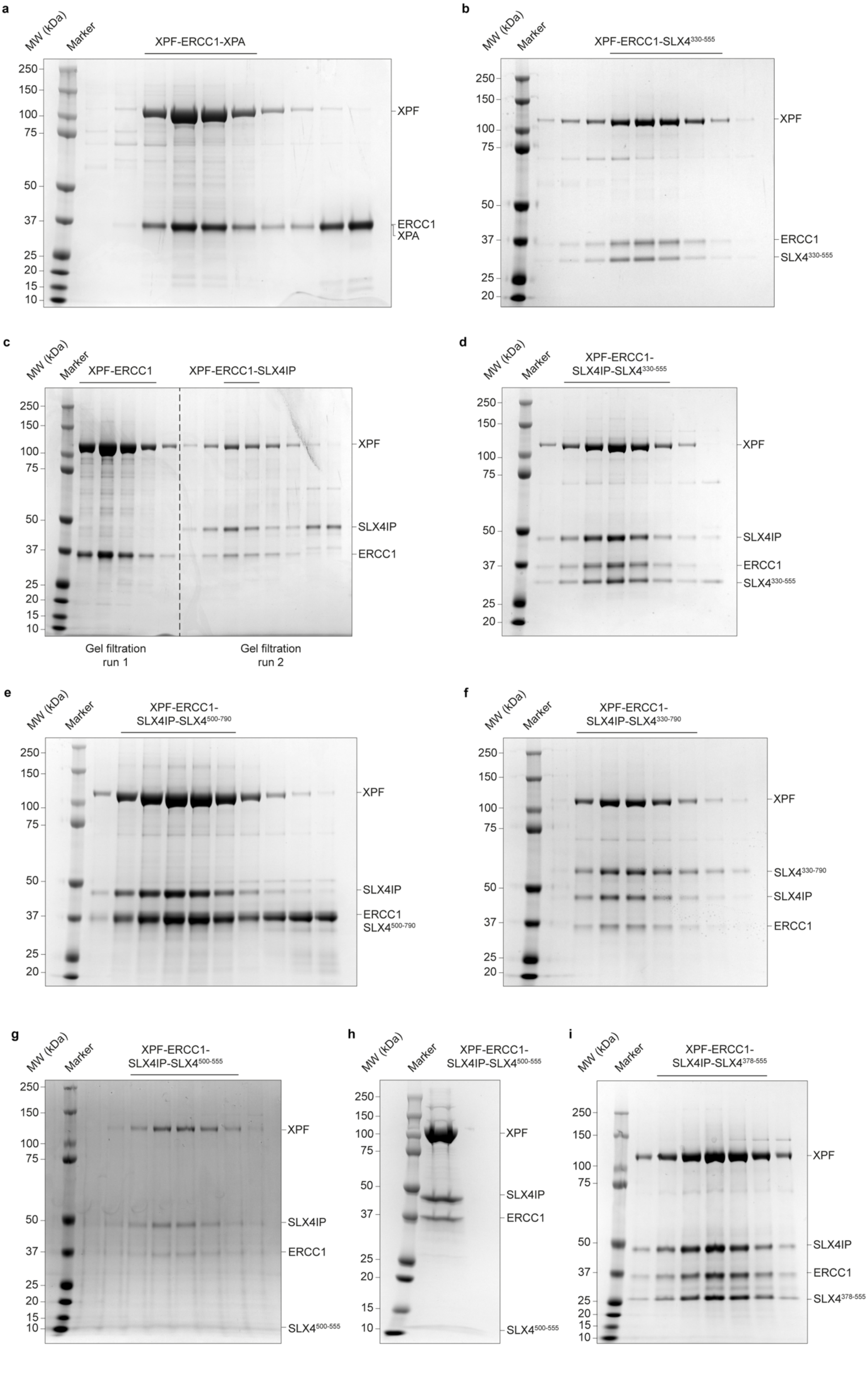

a
MW (kDa)
Marker
XPF-ERCC1-XPA
250
150
100
75
50
37
25
20
15
10
XPF
ERCC1
XPA
b
MW (kDa)
Marker
XPF-ERCC1-SLX4330-555
250
150
100
75
50
37
25
20
XPF
ERCC1
SLX4330-555
c
MW (kDa)
Marker
XPF-ERCC1
XPF-ERCC1-SLX4IP
250
150
100
75
50
37
25
20
15
10
XPF
SLX4IP
ERCC1
Gel filtration run 1
Gel filtration run 2
d
MW (kDa)
Marker
XPF-ERCC1-SLX4IP-SLX4330-555
250
150
100
75
50
37
25
20
XPF
SLX4IP
ERCC1
SLX4330-555
e
MW (kDa)
Marker
XPF-ERCC1-SLX4IP-SLX4500-790
250
150
100
75
50
37
25
20
XPF
SLX4IP
ERCC1
SLX4500-790
f
MW (kDa)
Marker
XPF-ERCC1-SLX4IP-SLX4330-790
250
150
100
75
50
37
25
20
XPF
SLX4330-790
SLX4IP
ERCC1
g
MW (kDa)
Marker
XPF-ERCC1-SLX4IP-SLX4500-555
250
150
100
75
50
37
25
20
15
10
XPF
SLX4IP
ERCC1
SLX4500-555
h
MW (kDa)
Marker
XPF-ERCC1-SLX4IP-SLX4500-555
250
150
100
75
50
37
25
20
15
10
XPF
SLX4IP
ERCC1
SLX4500-555
i
MW (kDa)
Marker
XPF-ERCC1-SLX4IP-SLX4378-555
250
150
100
75
50
37
25
20
15
10
XPF
SLX4IP
ERCC1
SLX4378-555

Supplementary Figure 2 | Protein purification. (**a**) Purification of XPF-ERCC1-XPA. SDS-PAGE analysis and Coomassie staining of fractions after size exclusion chromatography (Superdex 200 Increase 10/300 GL) is shown. A horizontal black bar indicates fractions collected, pooled, concentrated, and stored for subsequent experiments. ERCC1 and His_6-XPA run at the same apparent molecular weight. (**b**) Fractions of XPF-ERCC1-SLX4$^{330\text{-}555}$ after size exclusion chromatography (Superdex 200 Increase 10/300 GL). (**c**) Fractions of XPF-ERCC1 and XPF-ERCC1-SLX4IP after separate size exclusion chromatography runs (Superdex 200 Increase 10/300 GL) are shown. (**d**) Fractions of XPF-ERCC1-SLX4IP-SLX4$^{330\text{-}555}$ after size exclusion chromatography (Superdex 200 Increase 10/300 GL). (**e**) Fractions of XPF-ERCC1-SLX4IP-SLX4$^{500\text{-}790}$ after size exclusion chromatography (Superdex 200 Increase 10/300 GL). (**f**) Fractions of XPF-ERCC1-SLX4IP-SLX4$^{330\text{-}790}$ after size exclusion chromatography (Superdex 200 Increase 10/300 GL). (**g**) Fractions of XPF-ERCC1-SLX4IP-SLX4$^{500\text{-}555}$ after size exclusion chromatography (Superdex 200 Increase 10/300 GL). SLX4$^{500\text{-}555}$ runs at very low molecular weight and is poorly visualised. (**h**) SDS-PAGE analysis of a concentrated fraction of XPF-ERCC1-SLX4IP-SLX4$^{500\text{-}555}$ on an 4-20% Tris-glycine gel. SLX4$^{500\text{-}555}$ is visualised as a very low molecular weight band (see also Supplementary Fig. 7a-c). (**i**) Fractions of XPF-ERCC1-SLX4IP-SLX4$^{378\text{-}555}$ after size exclusion chromatography (Superdex 200 Increase 10/300 GL).

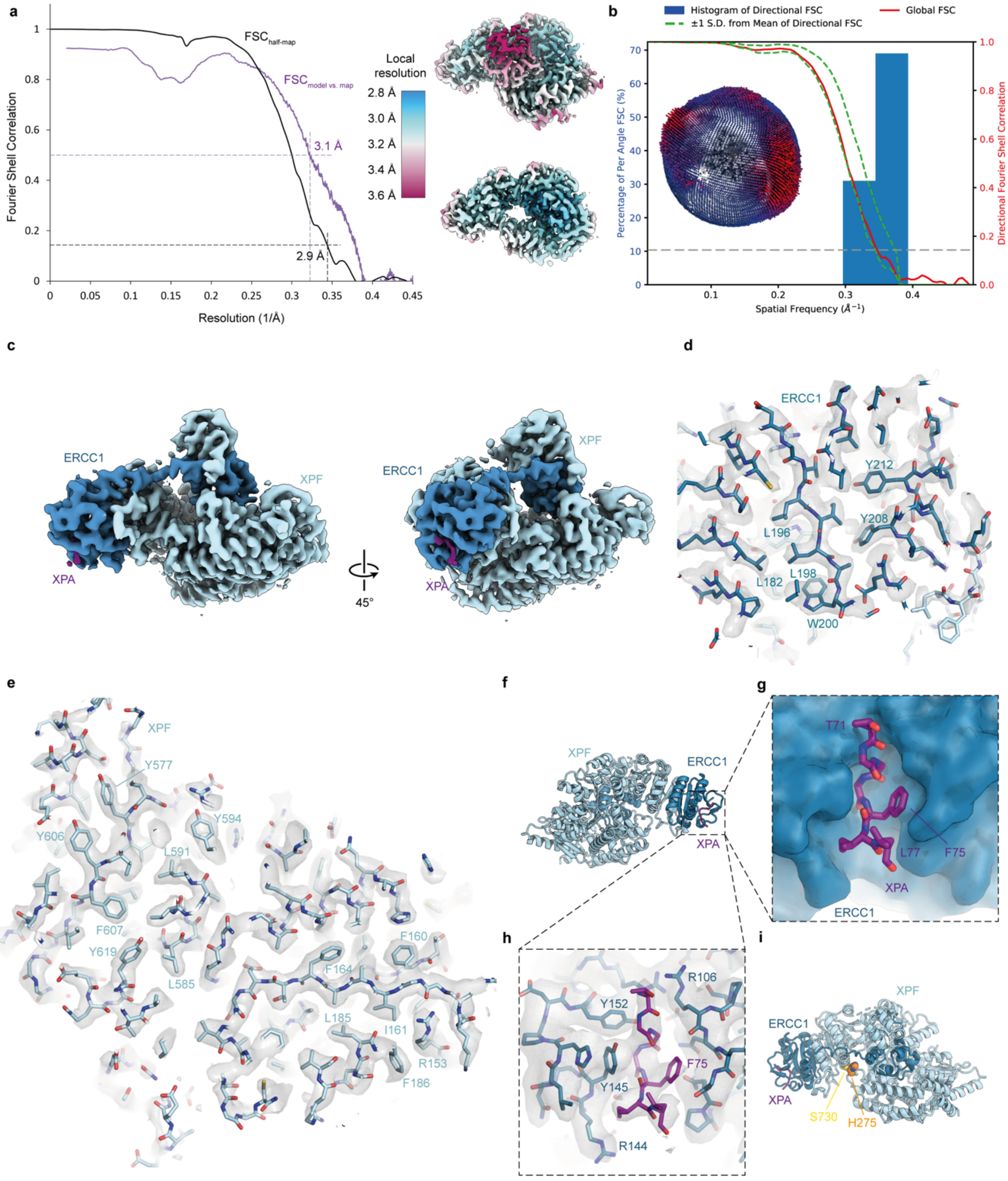

Supplementary Figure 3 | The structure of XPF-ERCC1-XPA and its validation. (**a**) Global (left) and local (right) resolution estimates by Fourier shell correlation. The FSC between half-maps is shown in black, and the model vs. map FSC is shown in purple. Resolutions at the appropriate FSC = 0.143 (half-maps) and FSC = 0.5 (model vs. map) thresholds [1] are indicated. The local resolution visualisation is shown in overview (top) and cut open (bottom) to additionally visualise the resolution at the core of the complex. Source data are provided in a Source Data file. (**b**) Histogram of directional FSCs for estimation of resolution anisotropy by the 3D FSC validation server. [2] Inset: Particle orientation distribution plot from RELION 3D auto-refinement. (**c**) Cryo-EM map of the XPF-ERCC1-XPA complex (XPF cyan, ERCC1 blue, XPA

purple; map low-pass filtered to 4 Å resolution to facilitate visualisation of the XPA peptide). (**d**, **e**) Slices through the structures of ERCC1 (d) and XPF (e) shown with the cryo-EM map. Large residues are labelled. (**f**-**h**) Structure of the XPA peptide in the context of the full structure (f), as a close-up view (g), and within the density (h). Due to higher flexibility and likely sub-stoichiometric binding of the XPA peptide, the map is filtered to 4 Å resolution in h. (**i**) Our structure assumes the auto-inhibited conformation reported previously [3], with an interaction between XPF residues H275 and S730.

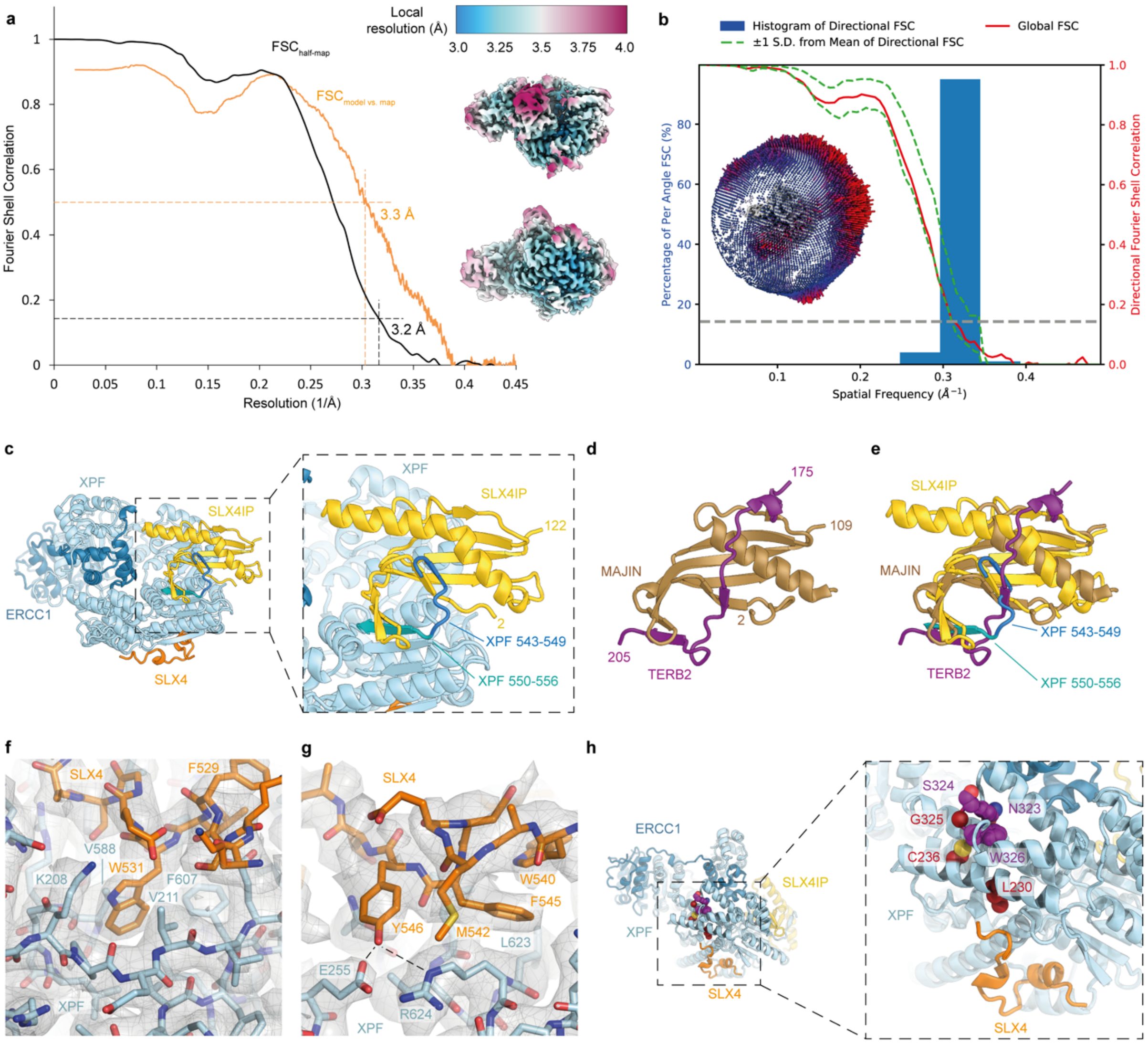

Supplementary Figure 4 | Cryo-EM structure of XPF-ERCC1-SLX4IP-SLX4$^{330\text{-}555}$. (**a**) Global (left) and local (right) resolution estimates by Fourier shell correlation. The FSC between half-maps is shown in black, and the model vs. map FSC is shown in orange. Resolutions at the FSC = 0.143 (half-maps) and FSC = 0.5 (model vs. map) thresholds [1] are indicated. The local resolution representation is shown in two views to visualise both SLX4IP and SLX4. Source data are provided in a Source Data file. (**b**) Histogram of directional FSCs for estimation of resolution anisotropy by the 3D FSC validation server. [2] Inset: Particle orientation distribution plot from RELION 3D auto-refinement. (**c**-**e**) Comparison of the structure of XPF-bound SLX4IP (c) with the X-ray crystal structure of MAJIN-TERB2 (d, e) (PDB ID 6GNX) [4]. (**f**, **g**) Molecular model of SLX4 residues 526-552 shown in the cryo-EM map. Large side chains are labelled. (**h**) Mapping of XPF mutations that impair the interaction with SLX4 onto our SLX4-bound structure. Point mutations in residues G325, L230, and C236 are coloured red; additional residues deleted in the Δ323-326 mutant are coloured purple.

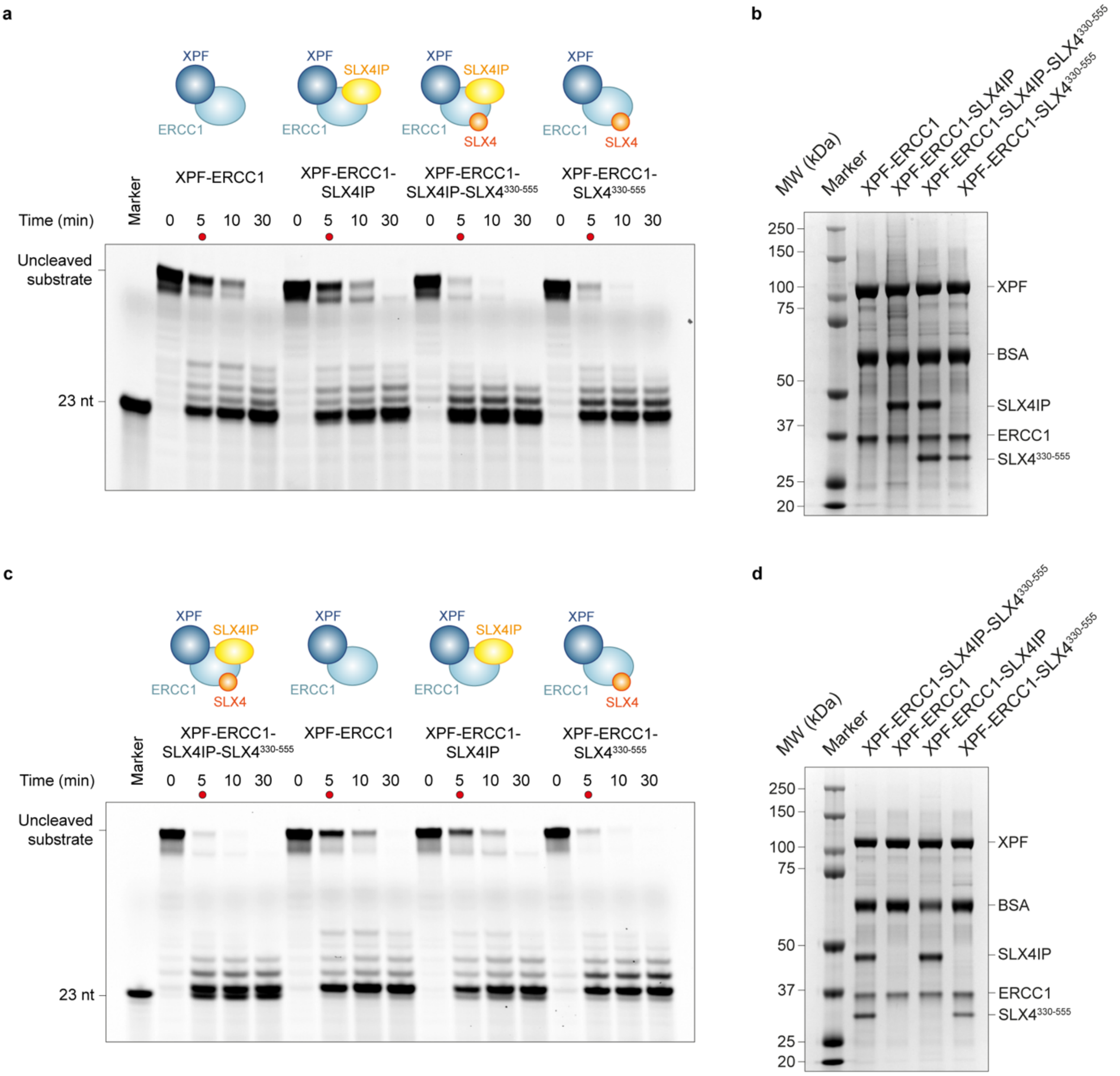

Supplementary Figure 5 | Replicates of nuclease assay shown in Fig. 3b, c. (**a**) Replicate 2 of nuclease activity assay using XPF-ERCC1, XPF-ERCC1-SLX4IP, XPF-ERCC1-SLX4$^{330-555}$, and XPF-ERCC1-SLX4IP-SLX4$^{330-555}$. Conversion of uncleaved input substrate into product (23-nt fragment, see marker lane) was monitored by detection of Cy3 fluorescence. To facilitate visualisation, 5 min time points are marked with a red dot. (**b**) Coomassie stained SDS-PAGE gel of protein sample processing control (before final 10x dilution into the nuclease reaction) to confirm that equivalent amounts of endonuclease were present in all samples in (a). (**c**, **d**) Replicate 3 of nuclease assay and protein sample processing control. Note that the loading order differs from repeats 1 and 2. Replicates were performed separately on different days.

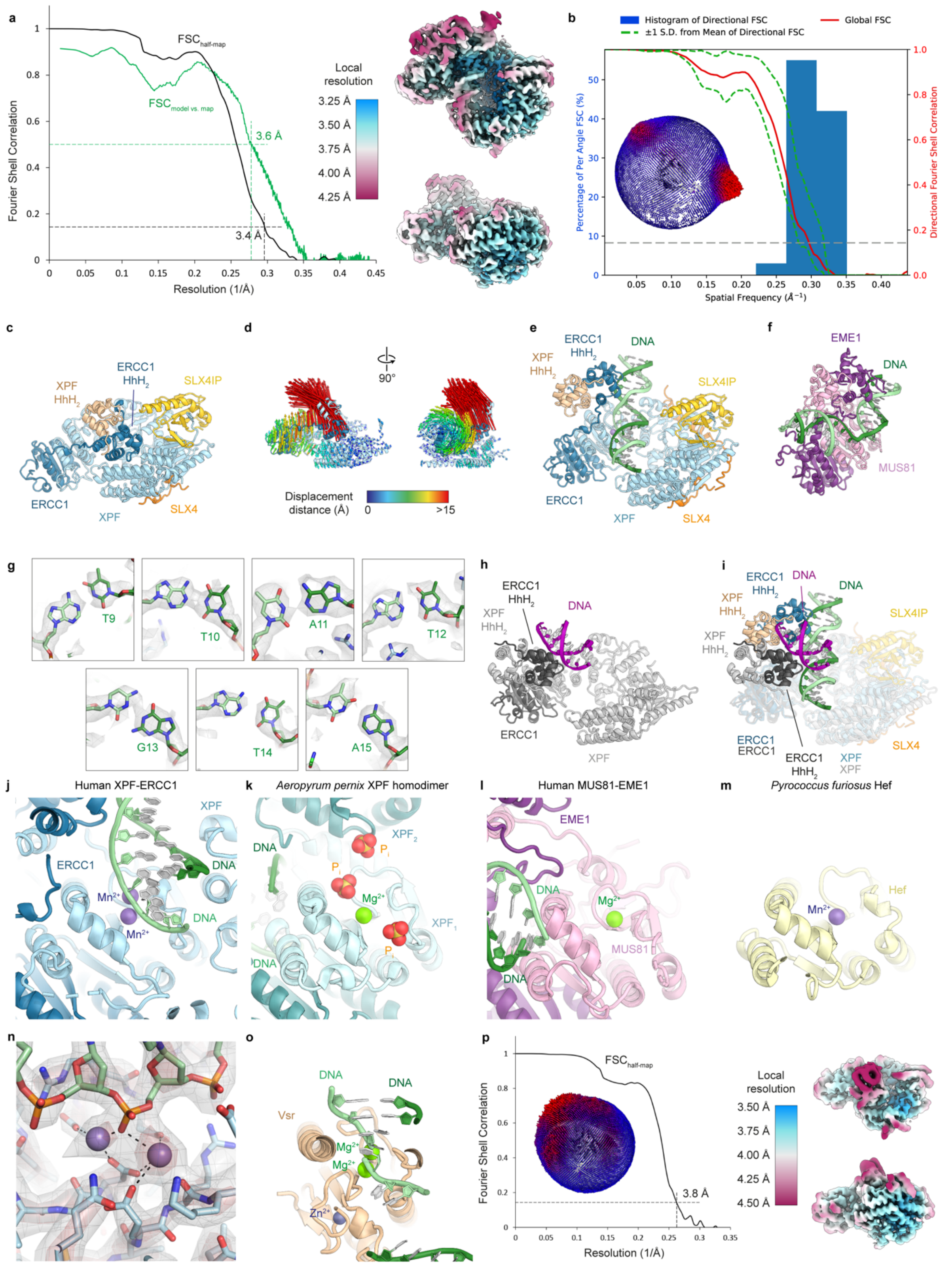

Supplementary Figure 6 | Cryo-EM structure of the XPF-ERCC1-SLX4IP-SLX4$^{330-555}$-DNA complex and its detailed analysis. (**a**) Global (left) and local (right) resolution estimates for the XPF-ERCC1-SLX4IP-SLX4$^{330-555}$-DNA complex by Fourier shell correlation. The FSC between half-maps is shown in black, and the model vs. map FSC is shown in green. Resolutions at the appropriate FSC = 0.143 (half-maps) and FSC = 0.5 (model vs. map) thresholds are indicated [1].

Source data are provided in a Source Data file. (**b**) Histogram of directional FSCs for estimation of resolution anisotropy by the 3D FSC validation server. [2] Inset: Particle orientation distribution plot from RELION 3D auto-refinement. (**c**-**e**) Comparison of the conformations of the free (c) and DNA-bound (e) XPF-ERCC1-SLX4IP-SLX4 complex along with a representation of protein Cα displacement vectors between the two states (d). (**f**) DNA path in the structure of MUS81-EME1 (PDB ID 4P0R) [5]. (**g**) Density for purines and pyrimidines in the cryo-EM map of the DNA-bound XPF-ERCC1-SLX4IP-SLX4 complex. (**h**, **i**). Conformational differences between our structure of DNA-bound XPF-ERCC1-SLX4IP-SLX4 (see panel e) and the structure of DNA-bound XPF-ERCC1 (PDB ID 6SXB) [3] (shown on its own in panel i and in superposition in panel j; proteins grey, DNA purple). The difference in DNA position is accompanied by a positional difference of the XPF-ERCC1 HhH_2 domain dimers between our complex and the prior XPF-ERCC1-DNA complex [3]. (**j**) Active site view of the structure of DNA-bound XPF-ERCC1-SLX4IP-SLX4. The structures in panels k-m and o have been superposed on this structure and are shown in the same reference view. (**k**) Active site view of the structure of an archaeal XPF dimer (PDB ID 2BGW) [6]. (**l**) Active site view of human MUS81-EME1 (PDB ID 4P0P) [5]. (**m**) Active site view of an archaeal Hef endonuclease (PDB ID 1J25) [7], which belongs to the same family as XPF and MUS81. (**n**) View of the cryo-EM density near the XPF active site with two Mn^{2+} ions shown as spheres. Density is shown at two thresholds (grey, lower threshold; red, higher threshold). (**o**) Active site view of the DNA-bound *Escherichia coli* Vsr endonuclease with two Mg^{2+} ions bound in the active site (PDB ID 1CW0) [8]. (**p**) Global (left) and local (right) resolution estimates for the DNA-free XPF-ERCC1-SLX4IP-$SLX4^{330-555}$ complex determined from the DNA-containing sample. The FSC between half-maps is shown in black and the resolution at the FSC = 0.143 threshold is indicated [1]. Inset: Particle orientation distribution plot from RELION 3D auto-refinement. Source data are provided in a Source Data file.

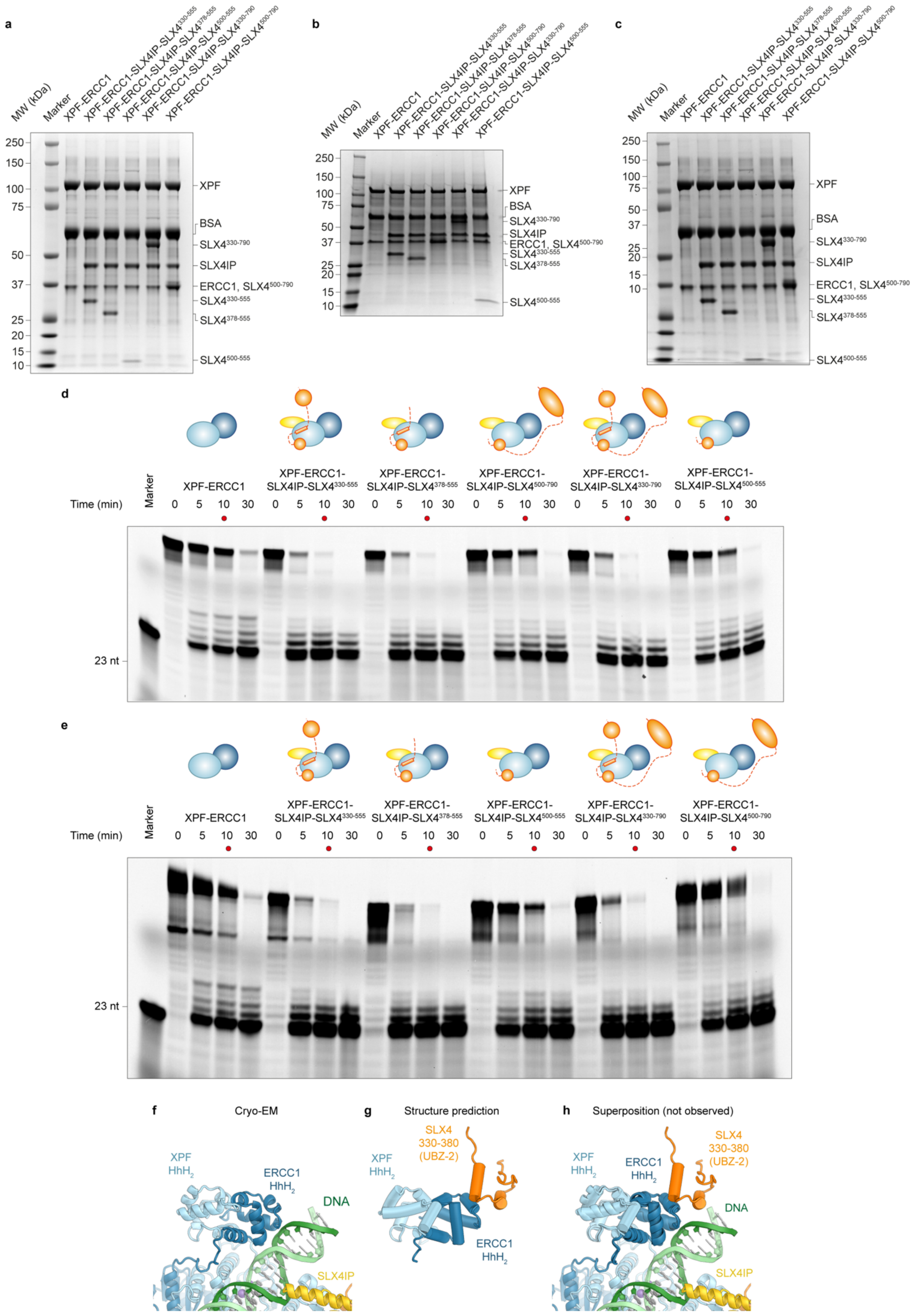

a
b
c
MW (kDa)
Marker
XPF-ERCC1
XPF-ERCC1-SLX4IP-SLX4330-555
XPF-ERCC1-SLX4IP-SLX4378-555
XPF-ERCC1-SLX4IP-SLX4500-555
XPF-ERCC1-SLX4IP-SLX4330-790
XPF-ERCC1-SLX4IP-SLX4500-790
XPF
BSA
SLX4330-790
SLX4IP
ERCC1, SLX4500-790
SLX4330-555
SLX4378-555
SLX4500-555
d
e
Time (min)
0 5 10 30
23 nt
f
Cryo-EM
g
Structure prediction
h
Superposition (not observed)
XPF HhH2
ERCC1 HhH2
DNA
SLX4IP
SLX4 330-380 (UBZ-2)

Supplementary Figure 7 | Effect of further SLX4 truncation on XPF-ERCC1 activity. (**a**) Protein sample processing control (before final 10x dilution into the nuclease reaction) for the endonuclease assay shown in Fig. 5c to confirm that equivalent amounts of endonuclease were present in all samples. (**b**-**e**) Additional repeats of the nuclease assay shown in Fig. 5c (b, d, sample processing control and nuclease assay for repeat 2; c, e, sample processing control and nuclease assay for repeat 3). To facilitate visualisation, 10 min time points are marked with a red dot. Note that the loading order differs for repeat 2 (panels b, d). Replicates were performed separately on different days. (**f**, **g**) Comparison of the DNA-bound structure of XPF-ERCC1-SLX4IP-SLX4$^{330-555}$ (f) and the predicted location of the SLX4 UBZ-2 domain on the ERCC1 HhH$_2$ domain (g, shown as cylindrical helices). (**h**) Superposition of the models shown in f and g.

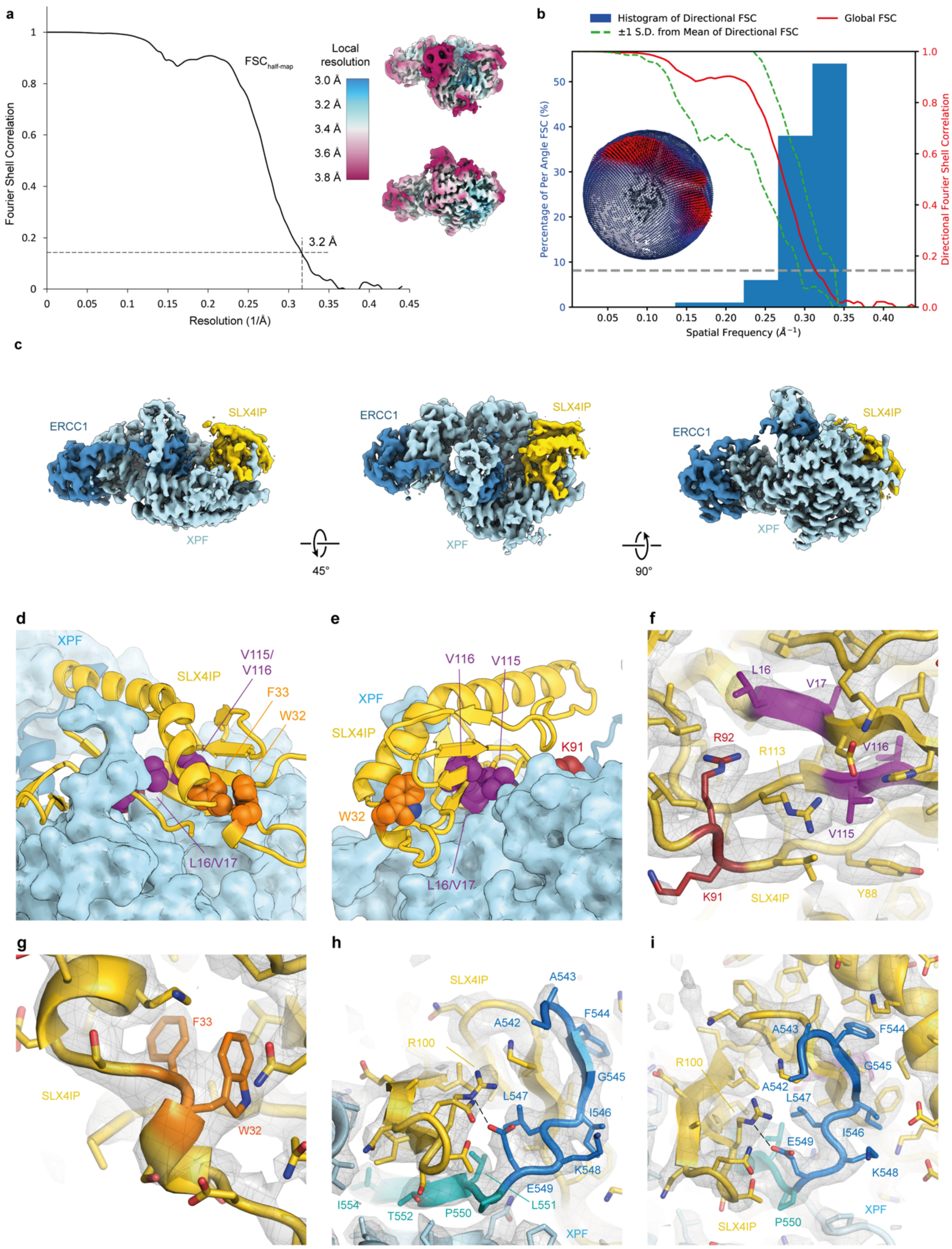

Supplementary Figure 8 | Cryo-EM structure of the XPF-ERCC1-SLX4IP complex and detailed analysis of residues involved in the XPF-SLX4IP interaction. (**a**) Global (left) and local (right) resolution estimates by Fourier shell correlation. The FSC between half-maps is shown in black. The resolution at the FSC = 0.143 threshold [1] is indicated. Source data are provided in a Source Data file. (**b**) Histogram of directional FSCs for estimation of resolution anisotropy by the 3D FSC validation server [2]. Inset: Particle orientation distribution plot from RELION 3D

auto-refinement. (**c**) Three views of the cryo-EM map revealing SLX4IP bound in the same location as in the XPF-ERCC1-SLX4IP-SLX4 complex even in the absence of SLX4. (**d**, **e**) SLX4IP residues reported to disrupt its interactions or localisation when mutated, shown in the context of the XPF-ERCC1-SLX4IP-SLX4 complex. (**f**) SLX4IP shown within the cryo-EM density. Important residues are coloured and labelled. Additional large residues supporting register assignment are labelled as well. (**g**) SLX4IP residues W32 and F33 shown in the cryo-EM density. (**h**, **i**) XPF residues involved in β-strand exchange are coloured, labelled, and shown with the cryo-EM density.

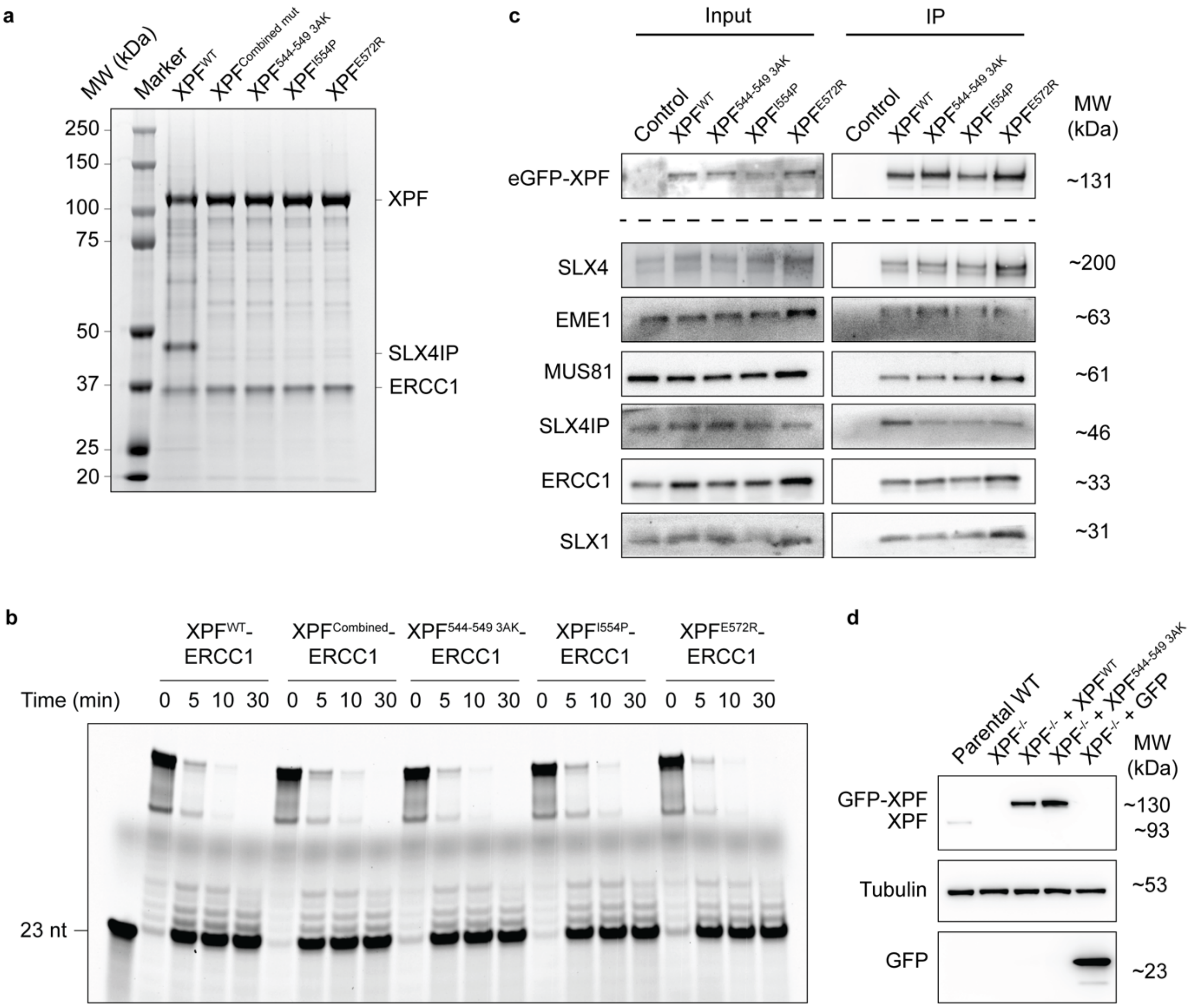

Supplementary Figure 9 | Characterisation of mutations that disrupt the interaction between SLX4IP and XPF. (**a**) Purified complexes containing mutant XPF-ERCC1. Single-residue mutants are denoted by the mutated residues. Other mutants are designated as follows: 544-549 3AK: F544A, I546A, L547A, E549K; combined: 544-549 3AK + I554P + E572R. (**b**) *In-vitro* endonuclease assay of mutant XPF-ERCC1. There is no discernible difference between the mutants and wild-type XPF-ERCC1. (**c**) IP of wild-type and mutant eGFP-XPF from HEK293TN cells. 1% of input lysate to the IP was analysed as input. (**d**) Expression of XPF in XPF knock-out and complemented cells.

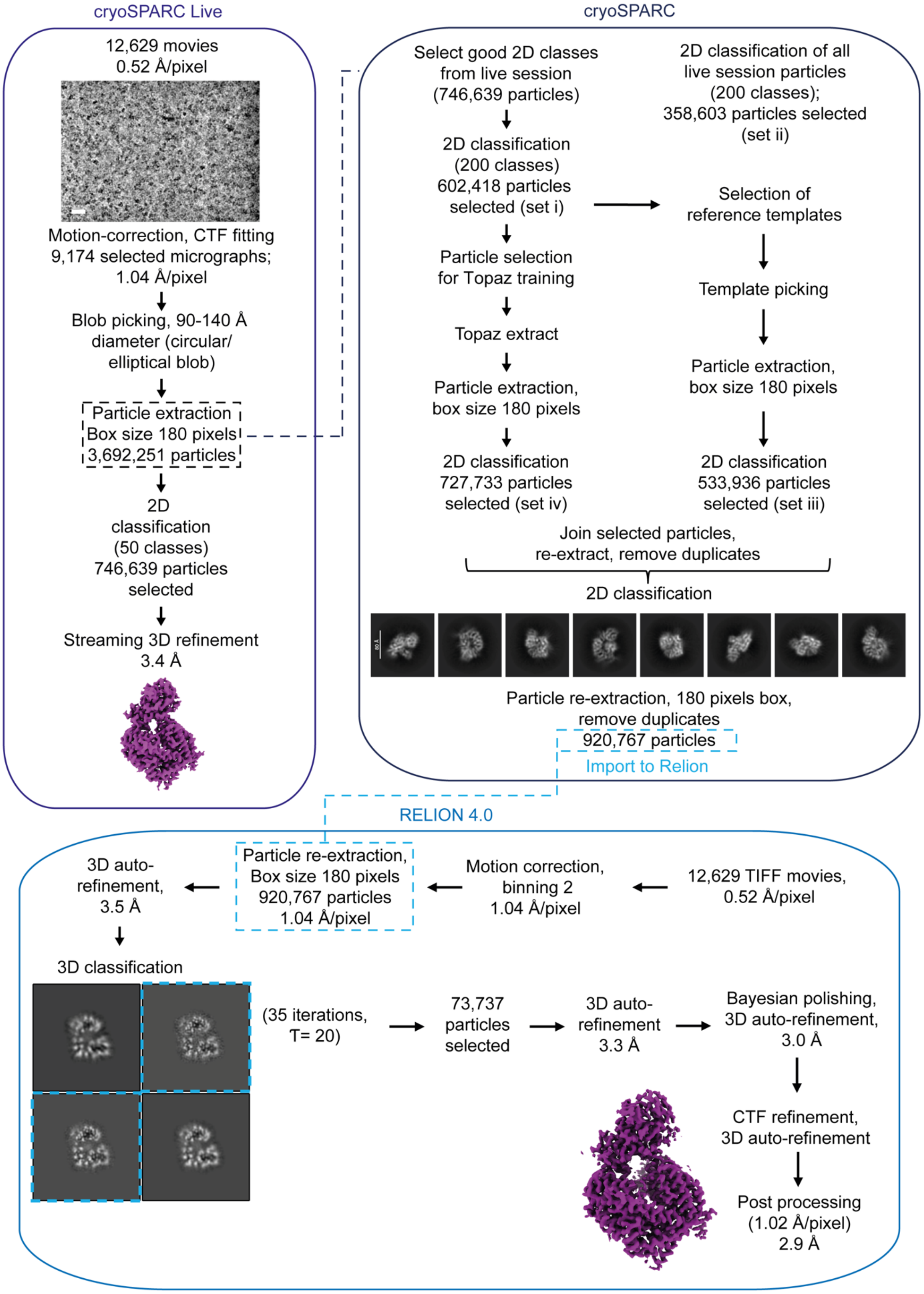

Supplementary Figure 10 | Image processing of XPF-ERCC1-XPA. See Methods for details. The sample micrograph was low-pass filtered to 20 Å for visualisation; scale bar: 200 Å.

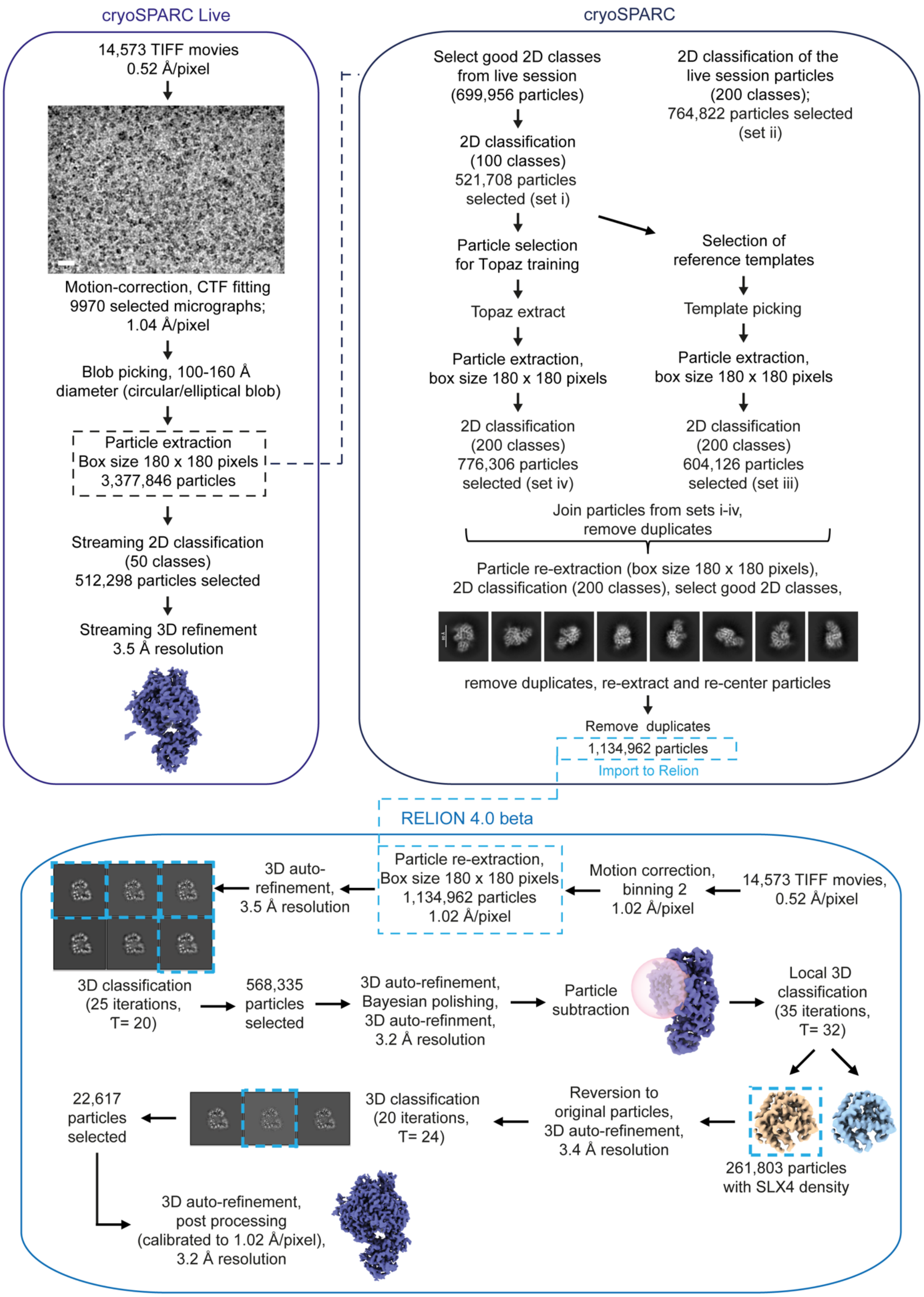

Supplementary Figure 11 | Image processing of XPF-ERCC1-SLX4IP-SLX4$^{330\text{-}555}$. See Methods for details. The sample micrograph was low-pass filtered to 20 Å for visualisation; scale bar: 200 Å.

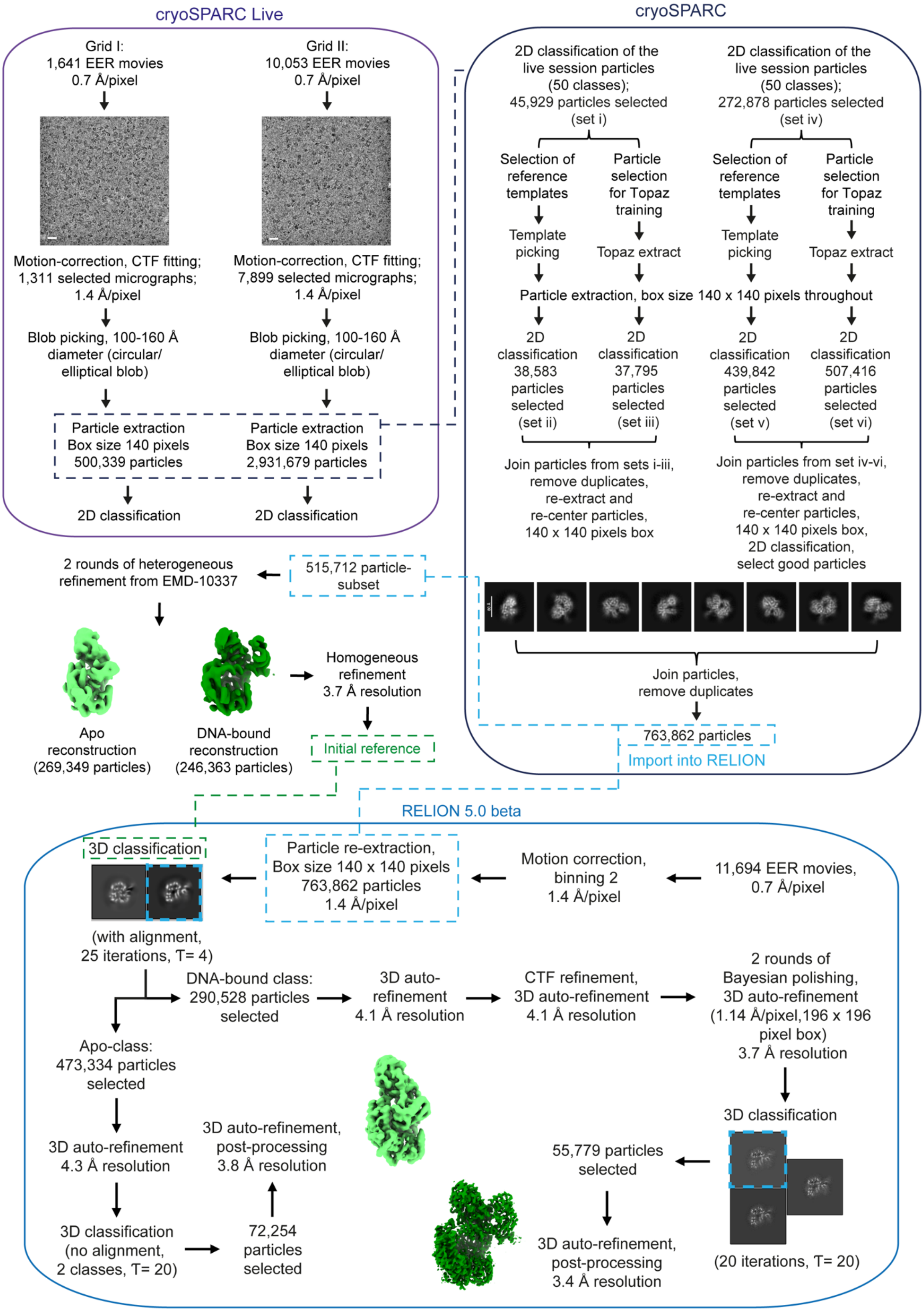
cryoSPARC Live
Grid I:
1,641 EER movies
0.7 Å/pixel
Grid II:
10,053 EER movies
0.7 Å/pixel
Motion-correction, CTF fitting;
1,311 selected micrographs;
1.4 Å/pixel
Motion-correction, CTF fitting;
7,899 selected micrographs;
1.4 Å/pixel
Blob picking, 100-160 Å
diameter (circular/
elliptical blob)
Blob picking, 100-160 Å
diameter (circular/
elliptical blob)
Particle extraction
Box size 140 pixels
500,339 particles
Particle extraction
Box size 140 pixels
2,931,679 particles
2D classification
2D classification
cryoSPARC
2D classification of the
live session particles
(50 classes);
45,929 particles selected
(set i)
2D classification of the
live session particles
(50 classes);
272,878 particles selected
(set iv)
Selection of
reference
templates
Particle
selection
for Topaz
training
Selection of
reference
templates
Particle
selection
for Topaz
training
Template
picking
Topaz extract
Template
picking
Topaz extract
Particle extraction, box size 140 x 140 pixels throughout
2D
classification
38,583
particles
selected
(set ii)
2D
classification
37,795
particles
selected
(set iii)
2D
classification
439,842
particles
selected
(set v)
2D
classification
507,416
particles
selected
(set vi)
Join particles from sets i-iii,
remove duplicates,
re-extract and
re-center particles,
140 x 140 pixels box
Join particles from set iv-vi,
remove duplicates,
re-extract and
re-center particles,
140 x 140 pixels box,
2D classification,
select good particles
Join particles,
remove duplicates
763,862 particles
Import into RELION
2 rounds of heterogeneous
refinement from EMD-10337
515,712 particle-
subset
Apo
reconstruction
(269,349 particles)
DNA-bound
reconstruction
(246,363 particles)
Homogeneous
refinement
3.7 Å resolution
Initial reference
RELION 5.0 beta
3D classification
(with alignment,
25 iterations, T= 4)
Particle re-extraction,
Box size 140 x 140 pixels
763,862 particles
1.4 Å/pixel
Motion correction,
binning 2
1.4 Å/pixel
11,694 EER movies,
0.7 Å/pixel
DNA-bound class:
290,528 particles
selected
3D auto-
refinement
4.1 Å resolution
CTF refinement,
3D auto-refinement
4.1 Å resolution
2 rounds of
Bayesian polishing,
3D auto-refinement
(1.14 Å/pixel,196 x 196
pixel box)
3.7 Å resolution
Apo-class:
473,334 particles
selected
3D auto-refinement
4.3 Å resolution
3D auto-refinement,
post-processing
3.8 Å resolution
3D classification
(no alignment,
2 classes, T= 20)
72,254
particles
selected
3D classification
55,779 particles
selected
3D auto-refinement,
post-processing
3.4 Å resolution
(20 iterations, T= 20)

Supplementary Figure 12 | Image processing of XPF-ERCC1-SLX4IP-SLX4-DNA. See Methods for details. The sample micrographs were low-pass filtered to 20 Å for visualisation; scale bar: 200 Å.

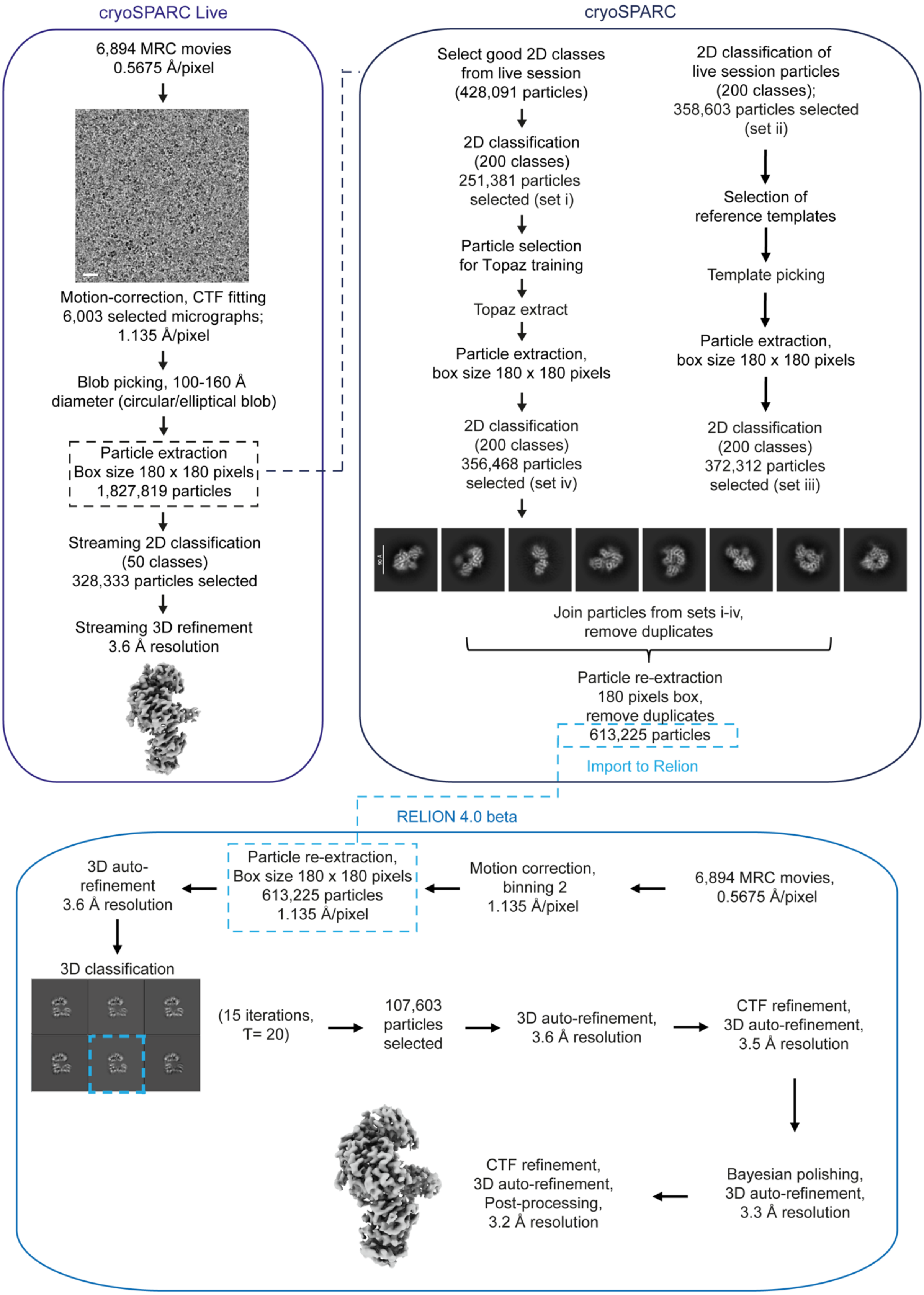

Supplementary Figure 13 | Image processing of XPF-ERCC1-SLX4IP. See Methods for details. The sample micrograph was low-pass filtered to 20 Å for visualisation; scale bar: 200 Å.

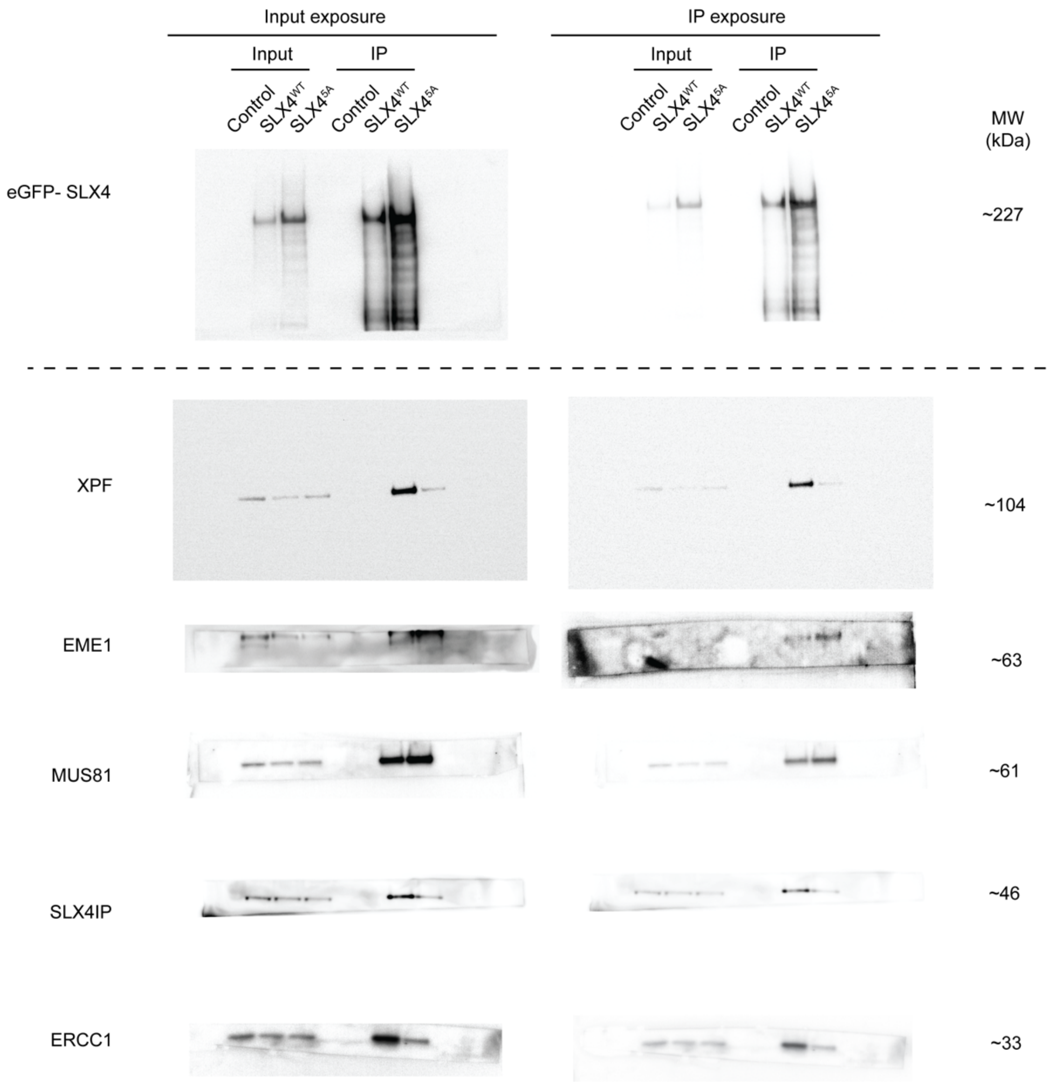

Supplementary Figure 14 | Uncropped membranes for Western blots in Fig. 2d. 10% of total sample volume was run independently to probe for bait (SLX4 or SLX4 5A-mutant) only. The remaining total sample volume was divided in half, and two Western blots were run. On the first Western blot membranes were probed against XPF, SLX4IP, and MUS81. The second membrane was probed with antibodies against EME1 and ERCC1. Membranes were cut horizontally and incubated with primary antibodies independently.

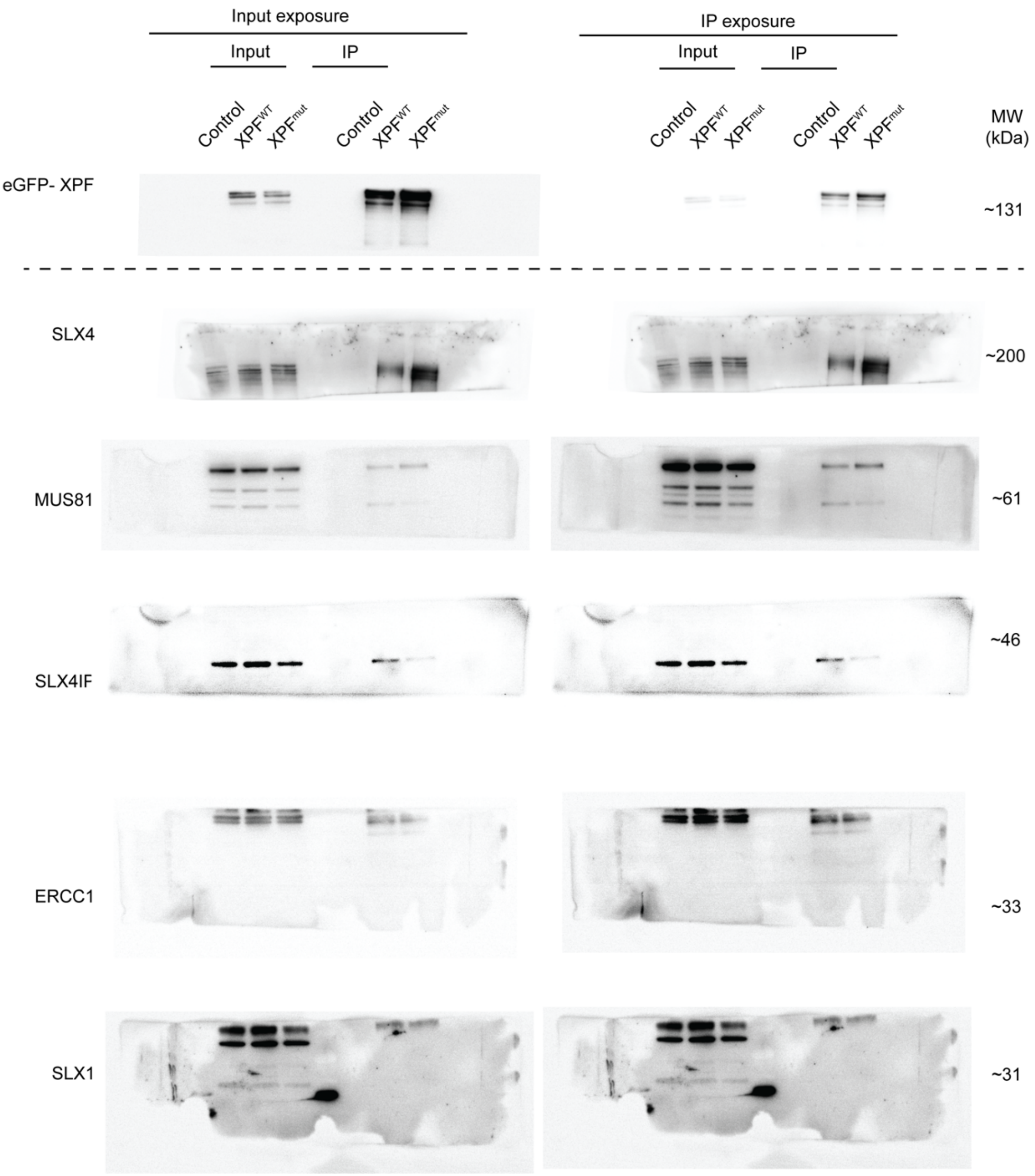

Supplementary Figure 15 | Uncropped membranes for Western blots in Fig. 7b. Total sample volume was divided in half, and two Western blots were performed. The first membrane was probed against SLX4, MUS81, and SLX1. The second membrane was probed against GFP, SLX4IP, and ERCC1. Membranes were cut horizontally and incubated with primary antibodies independently.

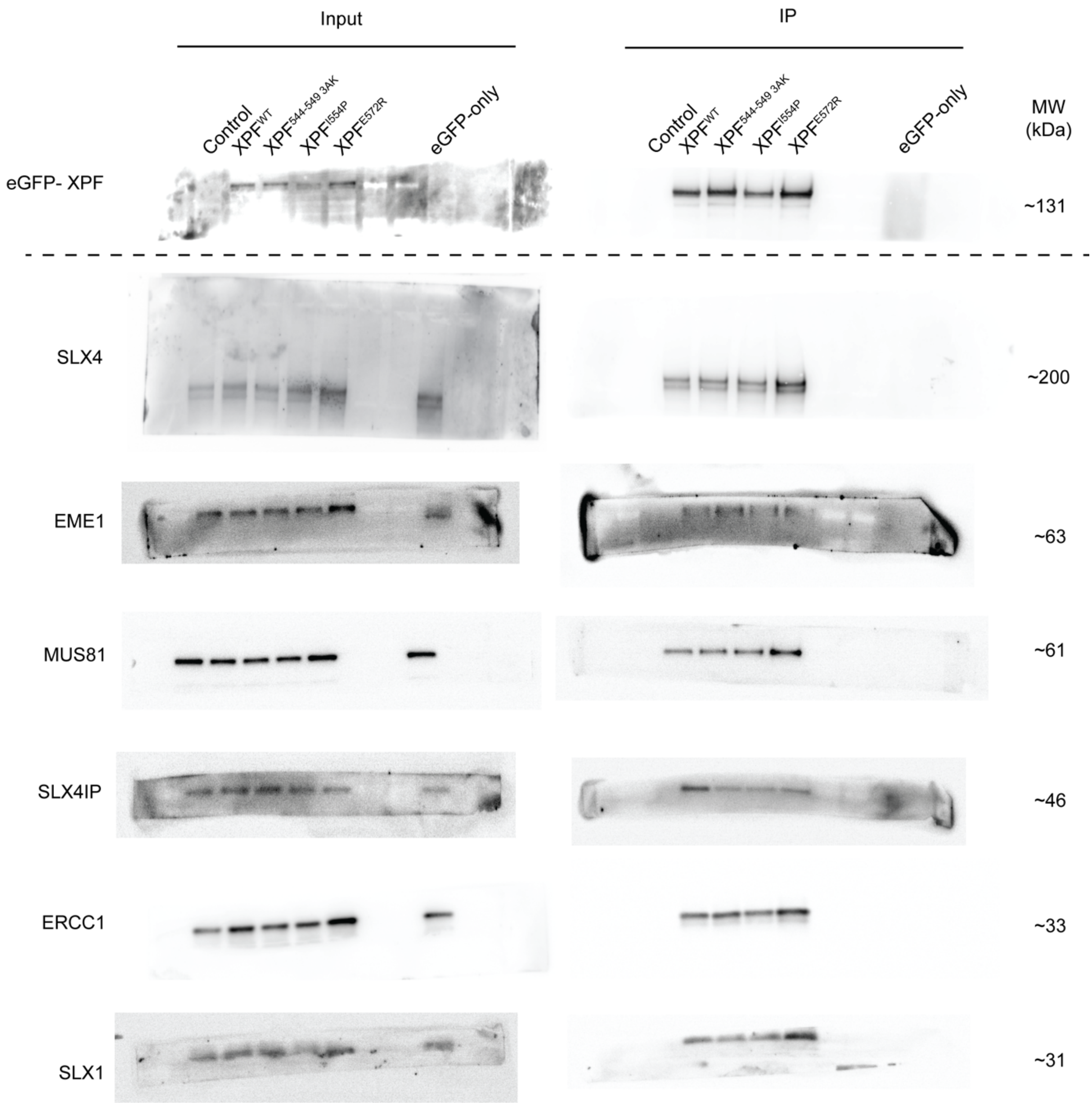

Supplementary Figure 16 | Uncropped membranes for Western blots in Supplementary Fig. 9c. Total sample volume was divided in half, and two Western blots were performed. The first membrane was probed against eGFP, SLX4, MUS81, and SLX1. The second membrane was probed against EME1, SLX4IP, and ERCC1. Membranes were cut horizontally and incubated with primary antibodies independently.

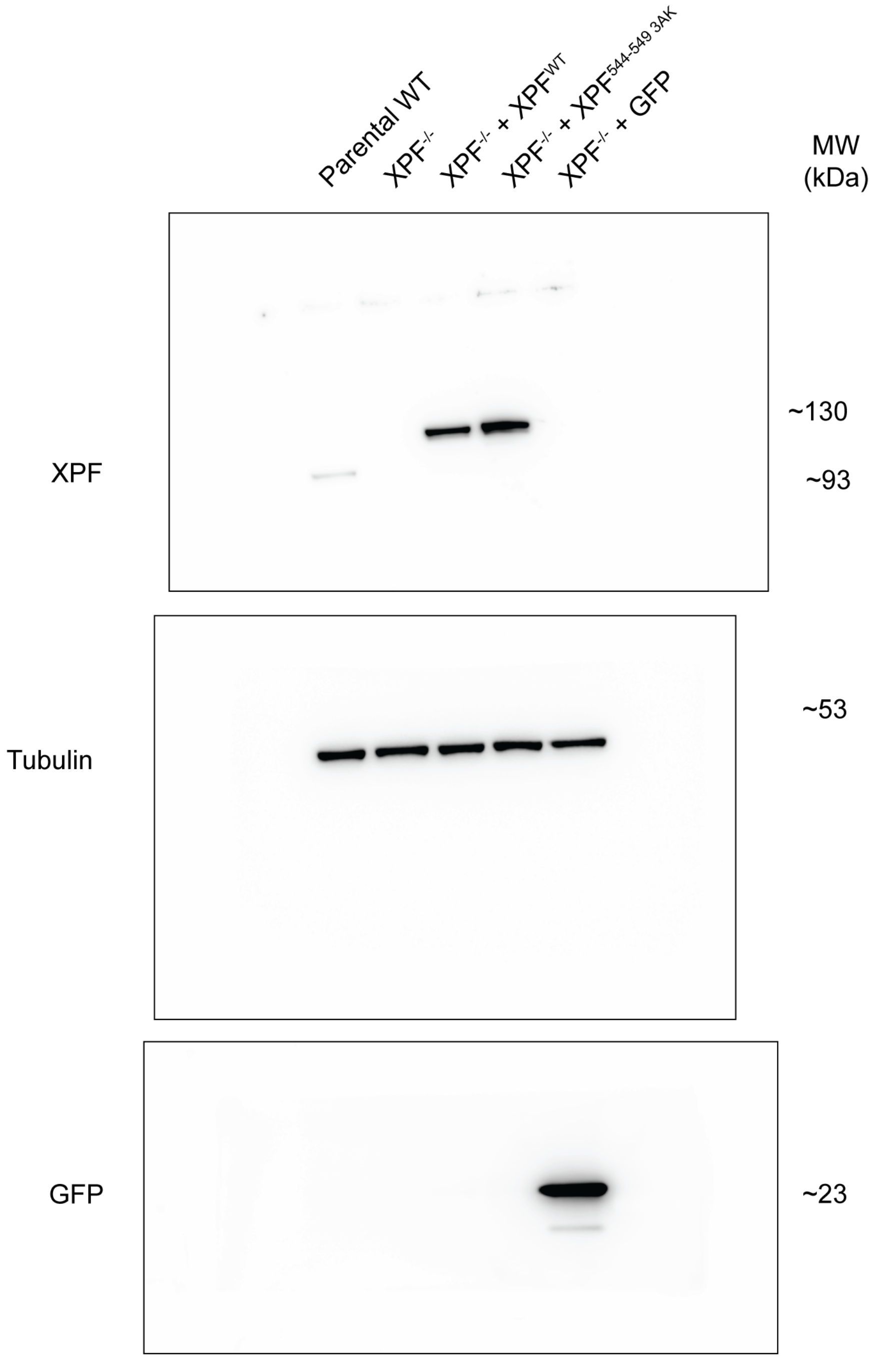

Supplementary Figure 17 | Uncropped membranes for Western blots in Supplementary Fig. 9d.

Supplementary Table 1 | Cryo-EM data collection, 3D reconstruction, and refinement statistics for XPF-ERCC1 complexes, part 1.

**Dataset**	**XPF-ERCC1-XPA**	**XPF-ERCC1-SLX4IP-SLX4$^{330-555}$**	**XPF-ERCC1-SLX4IP**
Microscope	Titan Krios G3i	Titan Krios G3i	Glacios
Stage type	Autoloader	Autoloader	Autoloader
Voltage (kV)	300	300	200
Detector	Gatan K3	Gatan K3	Falcon 4i
Energy filter	BioQuantum	BioQuantum	-
Acquisition mode	2x binning	2x binning	Counting
Pixel size (Å)	0.51	0.51	0.5675
Defocus range (µm underfocus)	0.4-2.5	1.0-2.5	0.7-1.7
Electron exposure (e^-/A^2)	70	70	60
**Reconstruction**	**EMD-53054**	**EMD-53055**	**EMD-53061**
Software	RELION 4.0	RELION 4.0	RELION 4.0
BLUSH used	No	No	No
Particles used	73,737	22,617	107,603
Box size (pixels)	180 x 180 x 180	180 x 180 x 180	180 x 180 x 180
Final pixel size (Å)	1.04	1.04	1.135
Accuracy rotations (°)	1.6	1.5	1.9
Accuracy translations (Å)	0.5	0.5	0.6
Map resolution (Å)	2.9	3.2	3.2
Map resolution range	2.8-3.5	3.0-3.5	3.0-3.5
Sphericity	0.97	0.97	0.89
Map sharpening B-factor ($Å^2$)	-75	-20	-50
**Coordinate refinement**			
Software	PHENIX	PHENIX	-
Refinement algorithm	REAL SPACE	REAL SPACE	-
Resolution cutoff (Å)	3.0	3.3	-
$FSC_{model-vs-map}$=0.5 (Å)	3.1	3.3	-
**Model**	**PDB-9QEC**	**PDB-9QED**	**-**
Number of residues	959	1088	-
Protein	959	1088	-
DNA	0	0	-
Ligand	0	0	-
B-factors overall	53.03	71.80	-
Protein	53.03	71.80	-
DNA	-	-	-
Ligand	-	-	-
R.M.S. deviations			
Bond lengths (Å)	0.002	0.004	-
Bond angles (°)	0.485	0.533	-
**Validation**			
Molprobity score	2.12	1.78	-
Molprobity clashscore	7.48	8.45	-
Rotamer outliers (%)	3.99	0.10	-
C_β deviations (%)	0.00	0.00	-
Ramachandran plot			
Favored (%)	96.30	95.34	-
Allowed (%)	3.39	4.66	-
Outliers (%)	0.32	0.00	-

Supplementary Table 2 | Cryo-EM data collection, 3D reconstruction, and refinement statistics for XPF-ERCC1 complexes, part 2.

**Dataset**	**XPF-ERCC1-SLX4IP-SLX4$^{330\text{-}555}$-DNA**	
Microscope	Glacios	
Stage type	Autoloader	
Voltage (kV)	200	
Detector	Falcon 4i	
Energy filter	Selectris	
Acquisition mode	Counting	
Pixel size (Å)	0.7	
Defocus range (μm underfocus)	0.4-1.7	
Electron exposure (e^-/A^2)	60	
**Reconstruction**	**EMD-53058**	**EMD-53059**
**Ligand**	**DNA**	**apo**
Software	RELION 5.0	RELION 5.0
BLUSH used	Yes	Yes
Particles used	55,799	72,254
Box size (pixels)	196 x 196 x 196	140 x 140 x 140
Final pixel size (Å)	1.14286	1.4
Accuracy rotations (°)	1.6	2.1
Accuracy translations (Å)	0.6	0.8
Map resolution (Å)	3.4	3.8
Map resolution range	3.2-5.0	3.5-6.0
Sphericity	0.92	-
Map sharpening B-factor ($Å^2$)	-70	-
**Coordinate refinement**		
Software	PHENIX	-
Refinement algorithm	REAL SPACE	-
Resolution cutoff (Å)	3.4	-
$FSC_{model\text{-}vs\text{-}map}$=0.5 (Å)	3.6	-
**Model**	**PDB-9QEE**	**-**
Number of residues	1162	-
Protein	1120	-
DNA	40	-
Ligand	2	-
B-factors overall	55.29	-
Protein	50.54	-
DNA	107.20	-
Ligand	42.67	-
R.M.S. deviations		
Bond lengths (Å)	0.006	-
Bond angles (°)	0.680	-
**Validation**		
Molprobity score	1.93	-
Molprobity clashscore	9.51	-
Rotamer outliers (%)	0.50	-
C_β deviations (%)	0.00	-
Ramachandran plot		
Favored (%)	93.56	-
Allowed (%)	6.44	-
Outliers (%)	0.00	-

SUPPLEMENTARY REFERENCES

1 Rosenthal, P. B. & Henderson, R. Optimal determination of particle orientation, absolute hand, and contrast loss in single-particle electron cryomicroscopy. *J. Mol. Biol.* **333**, 721-745 (2003). https://doi.org/10.1016/j.jmb.2003.07.013

2 Tan, Y. Z. *et al.* Addressing preferred specimen orientation in single-particle cryo-EM through tilting. *Nat. Meth.* **14**, 793-796 (2017). https://doi.org/10.1038/nmeth.4347

3 Jones, M. *et al.* Cryo-EM structures of the XPF-ERCC1 endonuclease reveal how DNA-junction engagement disrupts an auto-inhibited conformation. *Nat. Commun.* **11**, 1120-1114 (2020). https://doi.org/10.1038/s41467-020-14856-2

4 Dunce, J. M. *et al.* Structural basis of meiotic telomere attachment to the nuclear envelope by MAJIN-TERB2-TERB1. *Nat. Commun.* **9**, 5355 (2018). https://doi.org/10.1038/s41467-018-07794-7

5 Gwon, G. H. *et al.* Crystal structures of the structure-selective nuclease Mus81-Eme1 bound to flap DNA substrates. *EMBO J.* **33**, 1061-1072 (2014). https://doi.org/10.1002/embj.201487820

6 Newman, M. *et al.* Structure of an XPF endonuclease with and without DNA suggests a model for substrate recognition. *EMBO J.* **24**, 895-905 (2005). https://doi.org/10.1038/sj.emboj.7600581

7 Nishino, T., Komori, K., Ishino, Y. & Morikawa, K. X-ray and biochemical anatomy of an archaeal XPF/Rad1/Mus81 family nuclease: similarity between its endonuclease domain and restriction enzymes. *Structure* **11**, 445-457 (2003). https://doi.org/10.1016/S0969-2126(03)00046-7

8 Tsutakawa, S. E., Jingami, H. & Morikawa, K. Recognition of a TG Mismatch: The Crystal Structure of Very Short Patch Repair Endonuclease in Complex with a DNA Duplex. *Cell* **99**, 615-623 (1999). https://doi.org/10.1016/s0092-8674(00)81550-0
