# Supplementary material for: Molecular basis of XPF-ERCC1 targeting to SLX4-dependent DNA repair pathways
Source: Nat Commun. 2025 Dec 16;17:522. doi: 10.1038/s41467-025-67216-3 (PMC12804970; doi:10.1038/s41467-025-67216-3)
Supplement: Supplementary file 2 — Description of Additional Supplementary Files [file 41467_2025_67216_MOESM2_ESM.pdf]

Description of additional Supplementary Files

File name: Supplementary Data 1

Description: List of Oligonucleotide sequences used in this study.
