# Supplementary material for: Molecular basis of XPF-ERCC1 targeting to SLX4-dependent DNA repair pathways
Source: Nat Commun. 2025 Dec 16;17:522. doi: 10.1038/s41467-025-67216-3 (PMC12804970; doi:10.1038/s41467-025-67216-3)
Supplement: Supplementary file 4 — Reporting Summary [file 41467_2025_67216_MOESM4_ESM.pdf]

nature portfolio

Corresponding author(s): Basil J. Greber

Last updated by author(s): Nov 12, 2025

Reporting Summary

Nature Portfolio wishes to improve the reproducibility of the work that we publish. This form provides structure for consistency and transparency in reporting. For further information on Nature Portfolio policies, see our Editorial Policies and the Editorial Policy Checklist.

Statistics

For all statistical analyses, confirm that the following items are present in the figure legend, table legend, main text, or Methods section.

n/a	Confirmed	
☐	☒	The exact sample size (*n*) for each experimental group/condition, given as a discrete number and unit of measurement
☐	☒	A statement on whether measurements were taken from distinct samples or whether the same sample was measured repeatedly
☒	☐	The statistical test(s) used AND whether they are one- or two-sided<br>*Only common tests should be described solely by name; describe more complex techniques in the Methods section.*
☒	☐	A description of all covariates tested
☒	☐	A description of any assumptions or corrections, such as tests of normality and adjustment for multiple comparisons
☒	☐	A full description of the statistical parameters including central tendency (e.g. means) or other basic estimates (e.g. regression coefficient) AND variation (e.g. standard deviation) or associated estimates of uncertainty (e.g. confidence intervals)
☒	☐	For null hypothesis testing, the test statistic (e.g. *F*, *t*, *r*) with confidence intervals, effect sizes, degrees of freedom and *P* value noted<br>*Give P values as exact values whenever suitable.*
☒	☐	For Bayesian analysis, information on the choice of priors and Markov chain Monte Carlo settings
☒	☐	For hierarchical and complex designs, identification of the appropriate level for tests and full reporting of outcomes
☒	☐	Estimates of effect sizes (e.g. Cohen's *d*, Pearson's *r*), indicating how they were calculated

Our web collection on statistics for biologists contains articles on many of the points above.

Software and code

Policy information about availability of computer code

Data collection	Thermo Fisher Scientific EPU and EPU multi-grid (versions 3.4 (Krios) and 3.8.1 (Glacios)), Sherpa (version 1), and Digital Micrograph (version 3.53.41360; Krios collections only) were used for cryo-EM data collection.<br><br>Cell-titer glo assay data were collected using the Perkin Elmer 2030 software (version 4.0).
Data analysis	Cryo-EM data were analysed using cryoSPARC and RELION (version 4.0-beta and 5.0-beta). Atomic models were built in COOT version 0.9.6. Atomic coordinates were refined using PHENIX (versions 1.20, 1.21). Map and model visualisation, interpretation, and preparation of figures were performed in UCSF ChimeraX (version 1.6) and PyMOL (versions 2.5-3.0).<br><br>Cis-platin survival data were plotted in GraphPad Prism 10.6.1.

For manuscripts utilizing custom algorithms or software that are central to the research but not yet described in published literature, software must be made available to editors and reviewers. We strongly encourage code deposition in a community repository (e.g. GitHub). See the Nature Portfolio guidelines for submitting code & software for further information.

Data

Policy information about availability of data

All manuscripts must include a data availability statement. This statement should provide the following information, where applicable:

- Accession codes, unique identifiers, or web links for publicly available datasets
- A description of any restrictions on data availability
- For clinical datasets or third party data, please ensure that the statement adheres to our policy

The cryo-EM maps and atomic coordinate models for the XPF-ERCC1-XPA, XPF-ERCC1-SLX4IP-SLX4330-555, and XPF-ERCC1-SLX4IP-SLX4330-555-DNA complexes were deposited to the Electron Microscopy Data Bank (EMDB) and Protein Data Bank (PDB) with accession codes EMD-53054 [https://www.ebi.ac.uk/pdbe/entry/emdb/EMD-53054], EMD-53055 [https://www.ebi.ac.uk/pdbe/entry/emdb/EMD-53055], and EMD-53058 [https://www.ebi.ac.uk/pdbe/entry/emdb/EMD-53058], and PDB-9QEC [http://doi.org/10.2210/pdb9QEC/pdb], PDB-9QED [http://doi.org/10.2210/pdb9QED/pdb], and PDB-9QEE [http://doi.org/10.2210/pdb9QEE/pdb], respectively. The cryo-EM maps of the XPF-ERCC1-SLX4IP complex and the DNA-free XPF-ERCC1-SLX4IP-SLX4330-555 complex obtained from the same grid as the DNA-bound complex were deposited to the EMDB with accession codes EMD-53061 [https://www.ebi.ac.uk/pdbe/entry/emdb/EMD-53061] and EMD-53059 [https://www.ebi.ac.uk/pdbe/entry/emdb/EMD-53059]. Source data are provided with this paper. Requests for materials should be addressed to B.J.G.

Research involving human participants, their data, or biological material

Policy information about studies with human participants or human data. See also policy information about sex, gender (identity/presentation), and sexual orientation and race, ethnicity and racism.

Reporting on sex and gender	Our research did not involve human participants or animals.
Reporting on race, ethnicity, or other socially relevant groupings	Our research did not involve human participants or animals.
Population characteristics	Our research did not involve human participants or animals.
Recruitment	Our research did not involve human participants or animals.
Ethics oversight	Our research did not involve human participants or animals.

Note that full information on the approval of the study protocol must also be provided in the manuscript.

Field-specific reporting

Please select the one below that is the best fit for your research. If you are not sure, read the appropriate sections before making your selection.

☒ Life sciences ☐ Behavioural & social sciences ☐ Ecological, evolutionary & environmental sciences

For a reference copy of the document with all sections, see nature.com/documents/nr-reporting-summary-flat.pdf

Life sciences study design

All studies must disclose on these points even when the disclosure is negative.

Sample size	Dataset sizes of cryo-EM experiments were determined according to workflow requirements and reconstruction resolution targets (3.0-3.5 Å resolution).<br><br>Other experiments do not rely on statistical analysis, and samples sizes at n=2 or n=3 were chosen to ensure reproducibility of the results.
Data exclusions	Data exclusion during cryo-EM data collection and data processing was performed according to established standards in the field. Poor quality micrographs were excluded based on contamination with crystalline ice, high specimen motion leading to poor CTF fit estimates, and ice thickness. Cryo-EM particle images were classified using two- and three-dimensional classification algorithms to remove poor-quality particles.
Replication	During cryo-EM sample screening, we collected data from more than one (typically two) grids per specimen with consistent results between grids, except for quality differences that can be explained by the stochasticity of the grid preparation process.<br><br>Biochemical experiments (e.g. co-purification assays and co-immunoprecipitations) were performed twice to ensure reproducibility of the results. Endonuclease assays for comparison of nuclease activity between complexes with different subunit content were performed three times as technical replicates, and results of the repeats are provided in the Supplementary Materials. Cis-platin survival assays were repeated with three biological replicates and six technical replicates for each biological replicate, as described in the Materials Methods section and the figure legend of Fig. 7.
Randomization	The nature of this study does not require randomization because it does not involve a clinical trial or treatment allocation.

Blinding	The nature of this study does not require blinding because it does not involve a clinical trial or treatment allocation.

Reporting for specific materials, systems and methods

We require information from authors about some types of materials, experimental systems and methods used in many studies. Here, indicate whether each material, system or method listed is relevant to your study. If you are not sure if a list item applies to your research, read the appropriate section before selecting a response.

Materials & experimental systems

n/a	Involved in the study	
☐	☒	Antibodies
☐	☒	Eukaryotic cell lines
☒	☐	Palaeontology and archaeology
☒	☐	Animals and other organisms
☒	☐	Clinical data
☒	☐	Dual use research of concern
☒	☐	Plants

Methods

n/a	Involved in the study	
☒	☐	ChIP-seq
☒	☐	Flow cytometry
☒	☐	MRI-based neuroimaging

Antibodies

Antibodies used	Primary antibodies used were: GFP (Roche Cat# 11814460001, RRID:AB_390913, 1:500), MUS81 (Santa Cruz Biotechnology Cat# sc-47692, RRID:AB_2147129,1:500), ERCC1 (Santa Cruz Biotechnology Cat# sc-17809, RRID:AB_2278023,1:500), SLX4 (MRC-PPU Cat# S714C, RRID:AB_2752254, 1:500), SLX1 (Proteintech Cat# 21158-1-AP, RRID:AB_2752255, 1:500), EME1 (Santa Cruz Biotechnology Cat# sc-393363, 1:500), XPF (Bethyl Cat# A301-315A, RRID:AB_938089 1:500), and SLX4IP (Santa Cruz Biotechnology Cat# sc-377066, RRID:AB_2752253).<br><br>Secondary antibodies used were anti-mouse IgG-HRP (Dako, P0447, 1:2000), anti-rabbit IgG-HRP (Dako, P0448, 1:5000) and anti-sheep IgG-HRP (Abcam Cat# ab6747, RRID:AB_955453, 1:1000).
Validation	Use of previously validated antibodies, by siRNA/ CRISPR knock out, were used where possible. Use of antibodies that were also manufacturer validated and literature validated were also prioritised.

Eukaryotic cell lines

Policy information about cell lines and Sex and Gender in Research

Cell line source(s)	Insect cells: Our study used commercially available Spodoptera frugiperda Sf9 and Trichoplusia ni High5 insect cell lines purchased from Thermo Fisher (catalogue numbers 11496015 and B85502, respectively).<br><br>Human cells: Our study used (i) commercially available HEK293TN cells (Systems Biosciences, cat. no. LV900A-1) and (ii) RPE-1 p53-/- FRT/TR cells (initially provided by S. Jackson and maintained in the laboratory of W.N.).
Authentication	Cell lines were not authenticated.<br><br>The XPF knock-out cell line was verified using Western blot and a commercial deep sequencing service, as described in the manuscript.
Mycoplasma contamination	Human cells were regularly tested to confirm the absence of mycoplasma contamination.
Commonly misidentified lines (See ICLAC register)	None.

Plants

Seed stocks	Not applicable to this study.
Novel plant genotypes	Not applicable to this study.
Authentication	Not applicable to this study.
