# Supplementary figures and images for: Molecular basis of XPF-ERCC1 targeting to SLX4-dependent DNA repair pathways
Source: Nat Commun. 2025 Dec 16;17:522. doi: 10.1038/s41467-025-67216-3 (PMC12804970; doi:10.1038/s41467-025-67216-3)

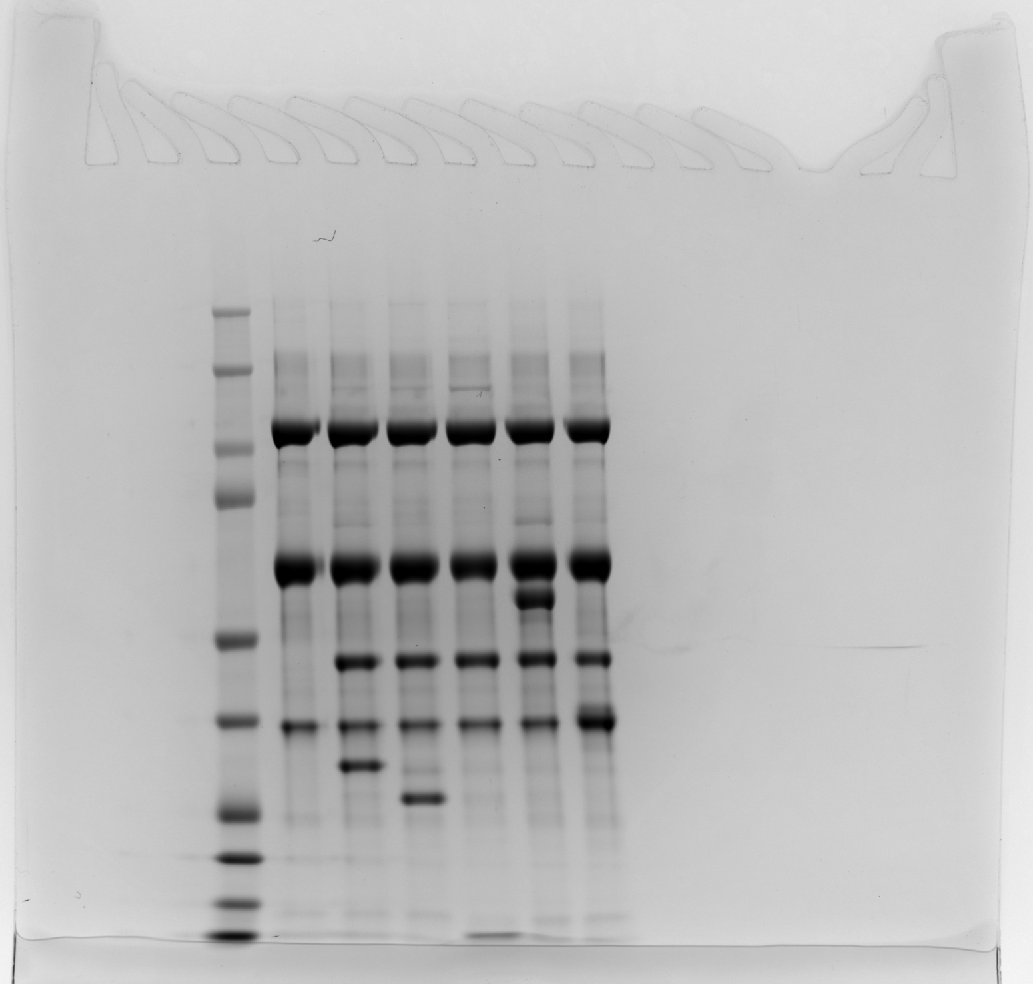

Supplement: Supplementary file 6 — Source data [file 41467_2025_67216_MOESM6_ESM.zip › Feng_etal_Source_Data/SupplementaryFig7/SupFig7c_uncropped.tif]

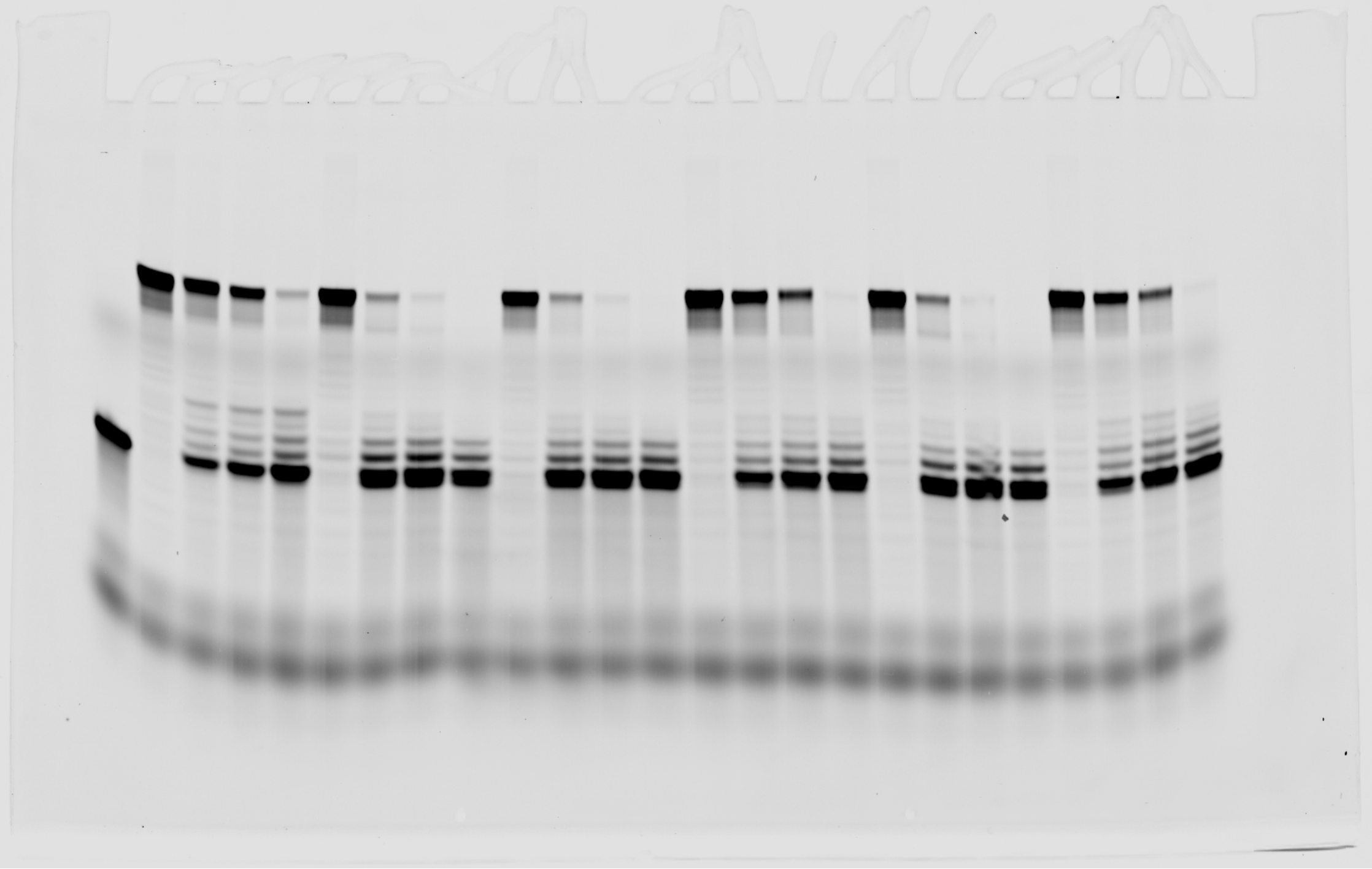

Supplement: Supplementary file 6 — Source data [file 41467_2025_67216_MOESM6_ESM.zip › Feng_etal_Source_Data/SupplementaryFig7/SupFig7d_uncropped.tif]

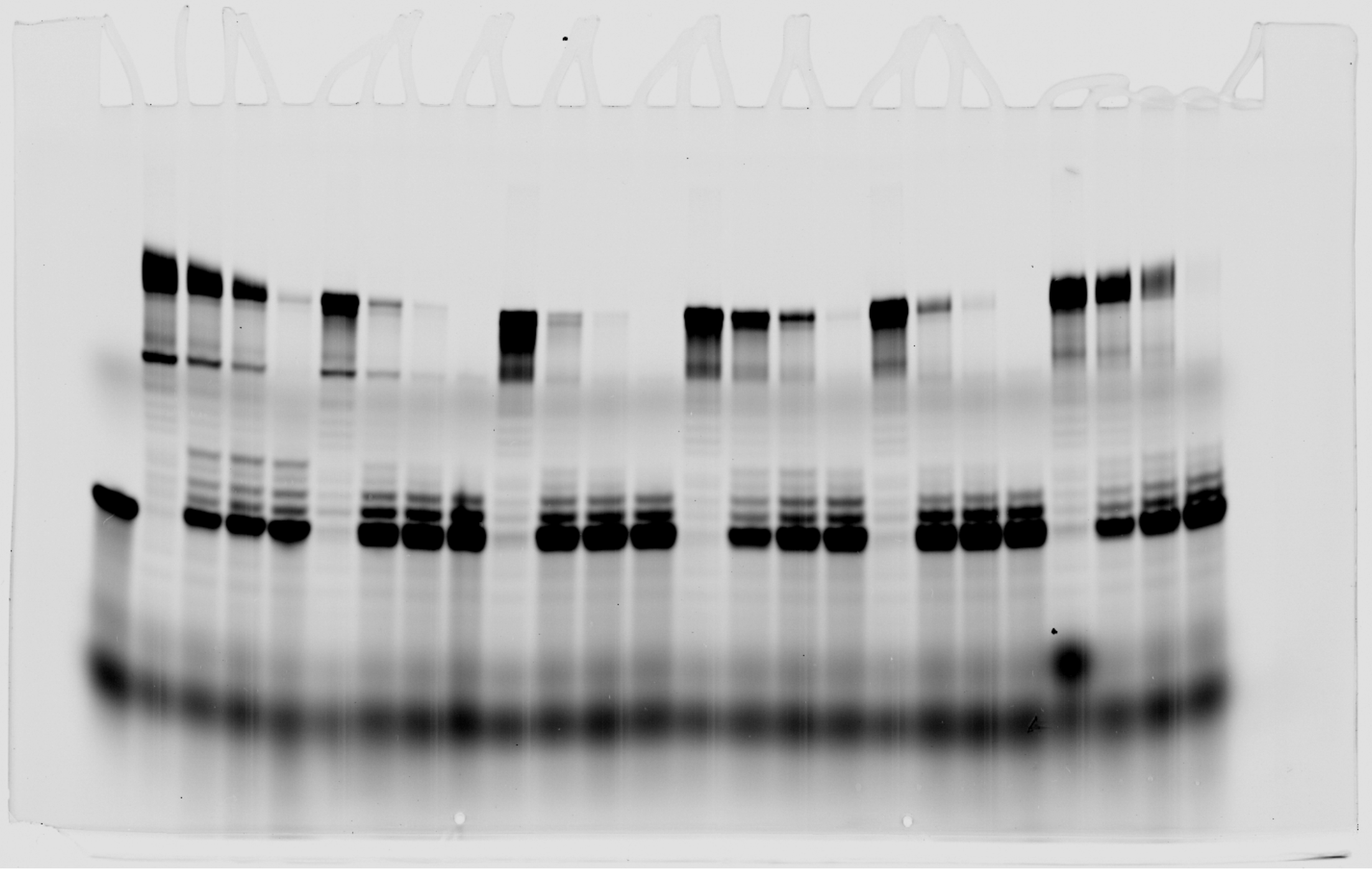

Supplement: Supplementary file 6 — Source data [file 41467_2025_67216_MOESM6_ESM.zip › Feng_etal_Source_Data/SupplementaryFig7/SupFig7e_uncropped.tif]

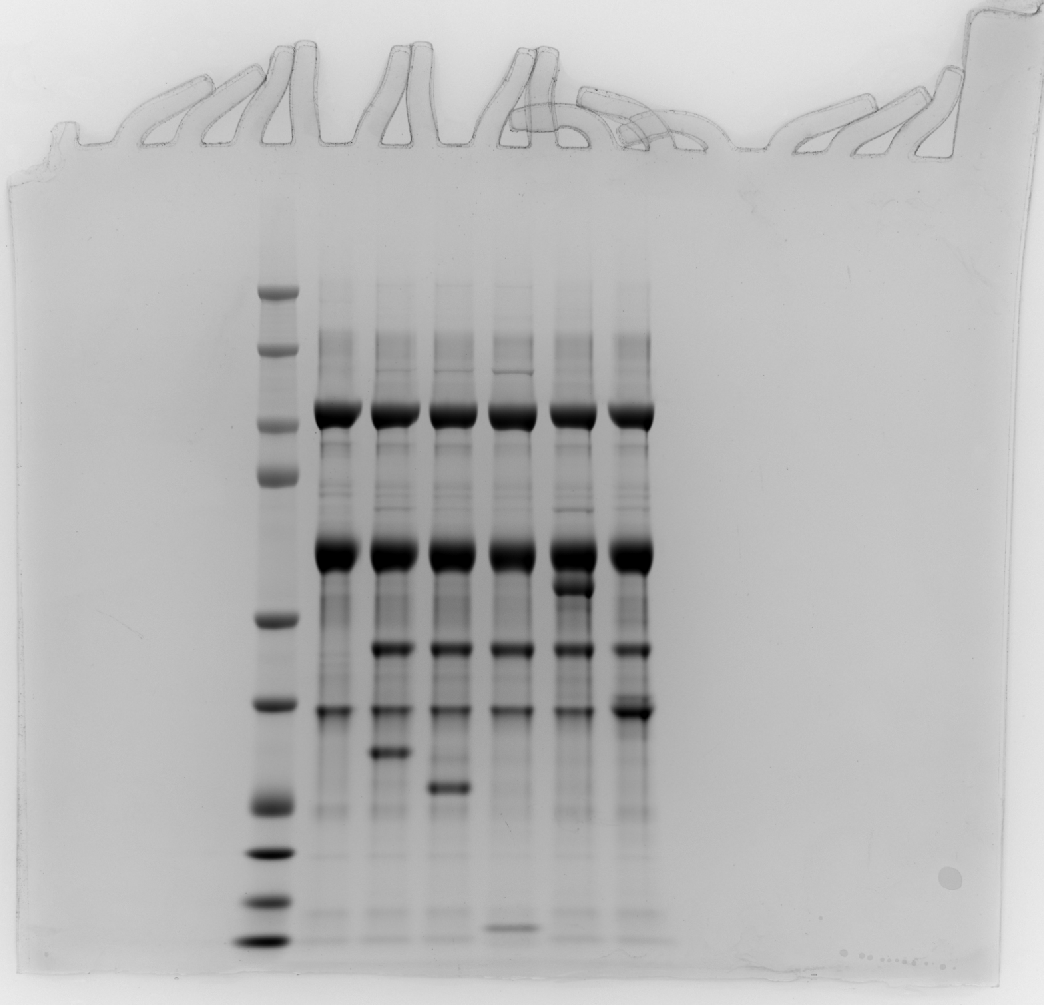

Supplement: Supplementary file 6 — Source data [file 41467_2025_67216_MOESM6_ESM.zip › Feng_etal_Source_Data/SupplementaryFig7/SupFig7a_uncropped.tif]

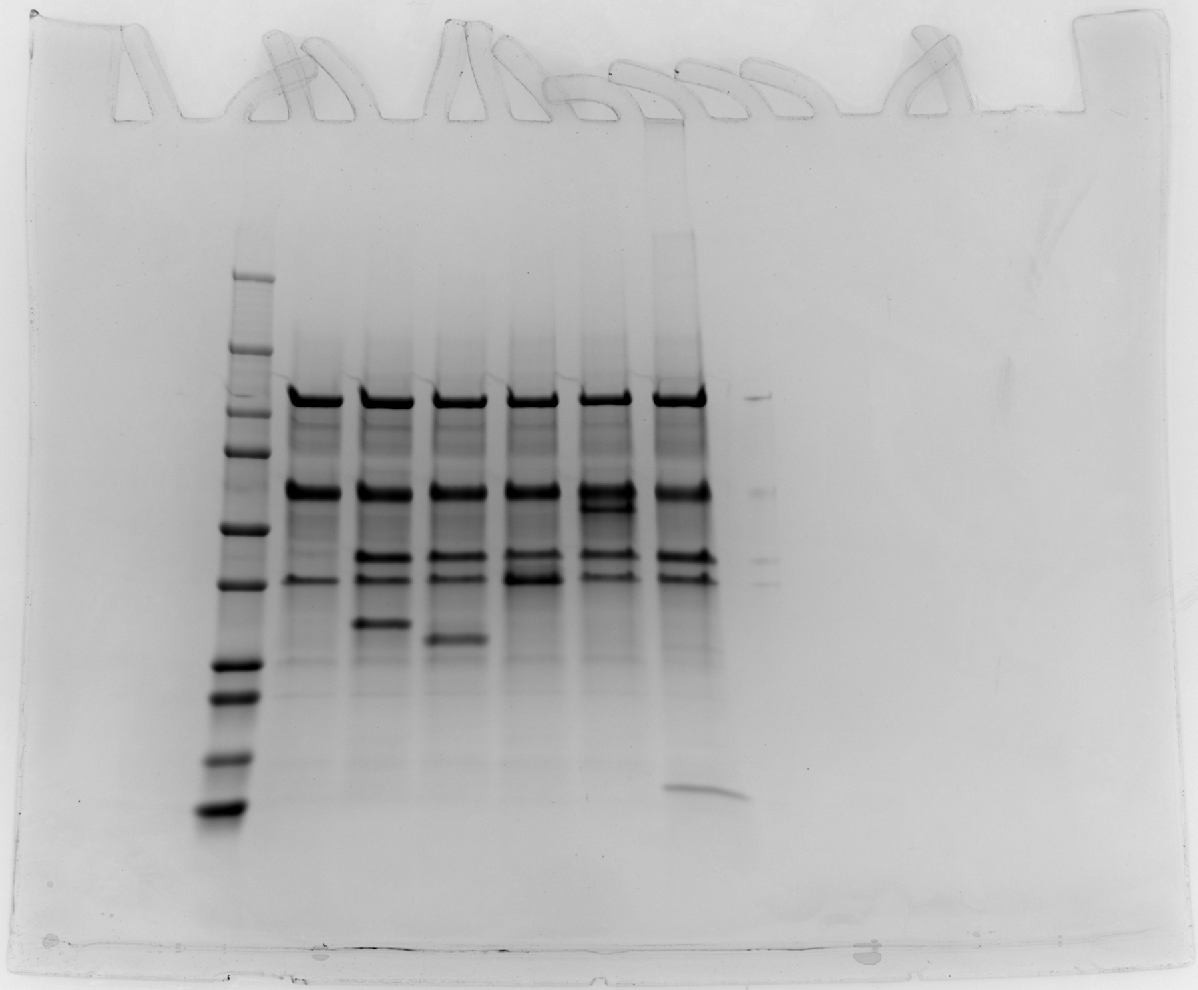

Supplement: Supplementary file 6 — Source data [file 41467_2025_67216_MOESM6_ESM.zip › Feng_etal_Source_Data/SupplementaryFig7/SupFig7b_uncropped.tif]

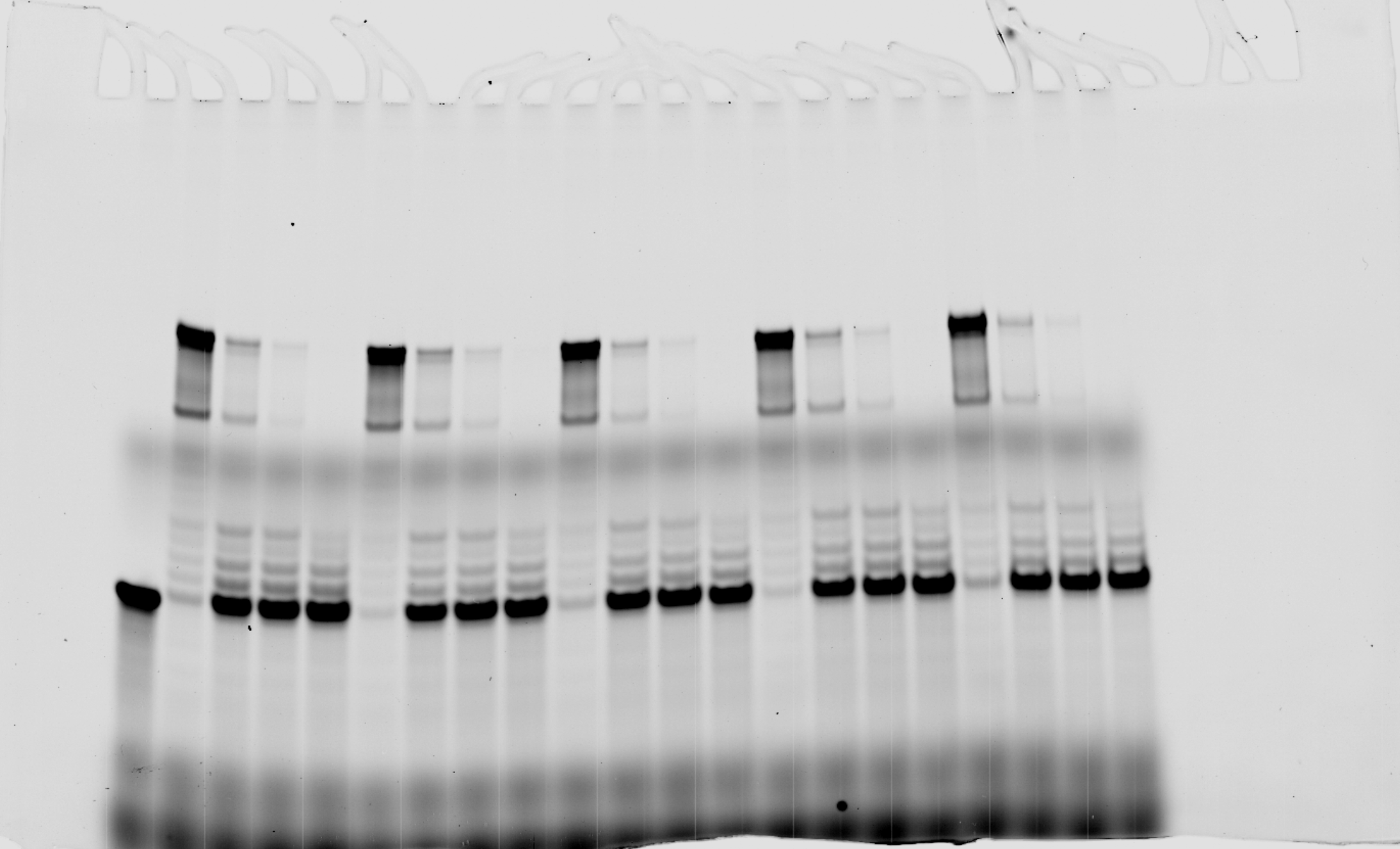

Supplement: Supplementary file 6 — Source data [file 41467_2025_67216_MOESM6_ESM.zip › Feng_etal_Source_Data/SupplementaryFig9/SupFig9b_uncropped.tif]

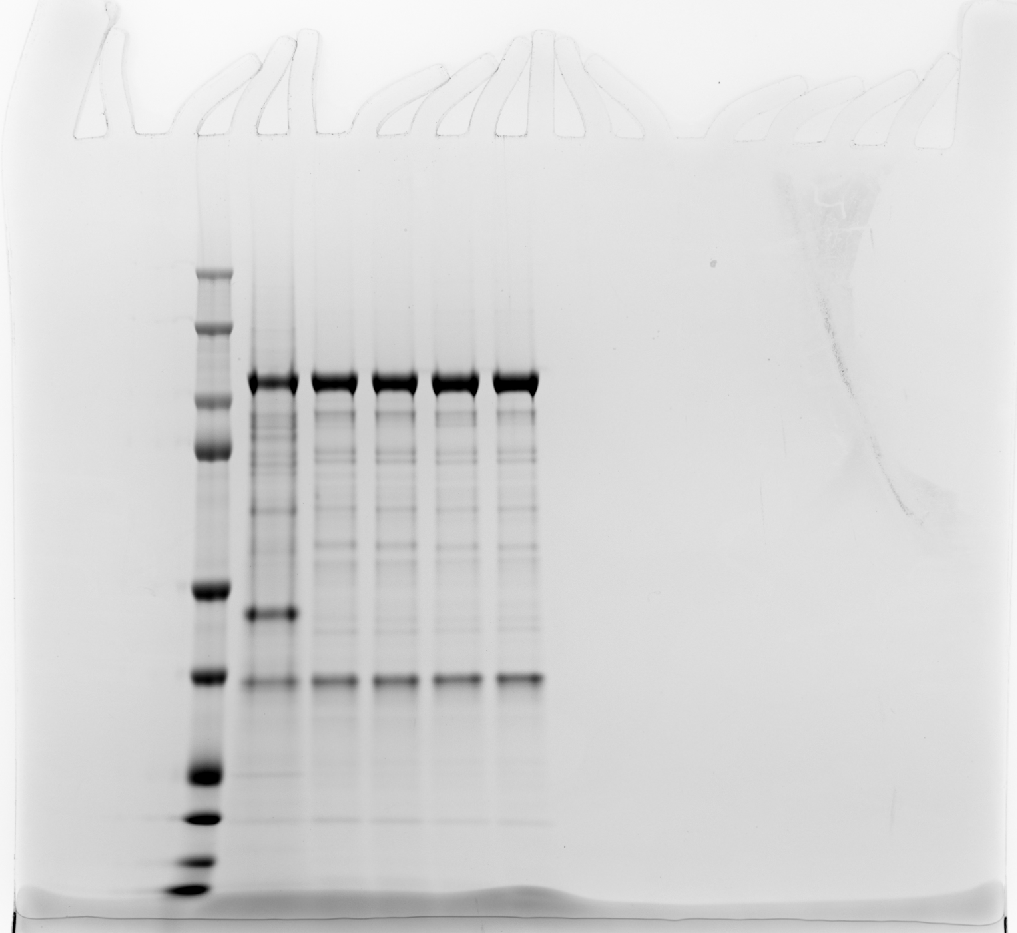

Supplement: Supplementary file 6 — Source data [file 41467_2025_67216_MOESM6_ESM.zip › Feng_etal_Source_Data/SupplementaryFig9/SupFig9a_uncropped.tif]

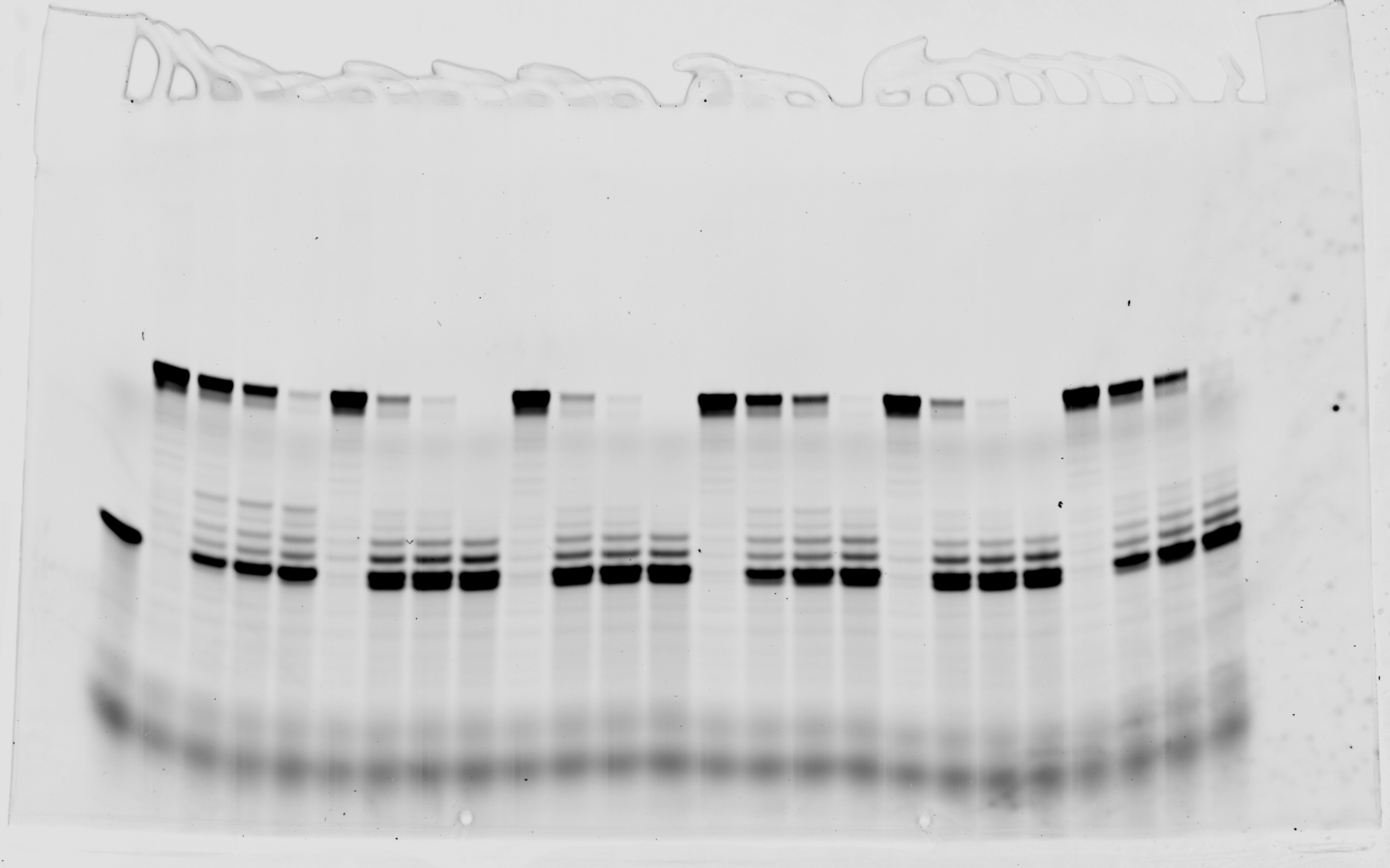

Supplement: Supplementary file 6 — Source data [file 41467_2025_67216_MOESM6_ESM.zip › Feng_etal_Source_Data/Fig5/Fig5c_uncropped.tif]

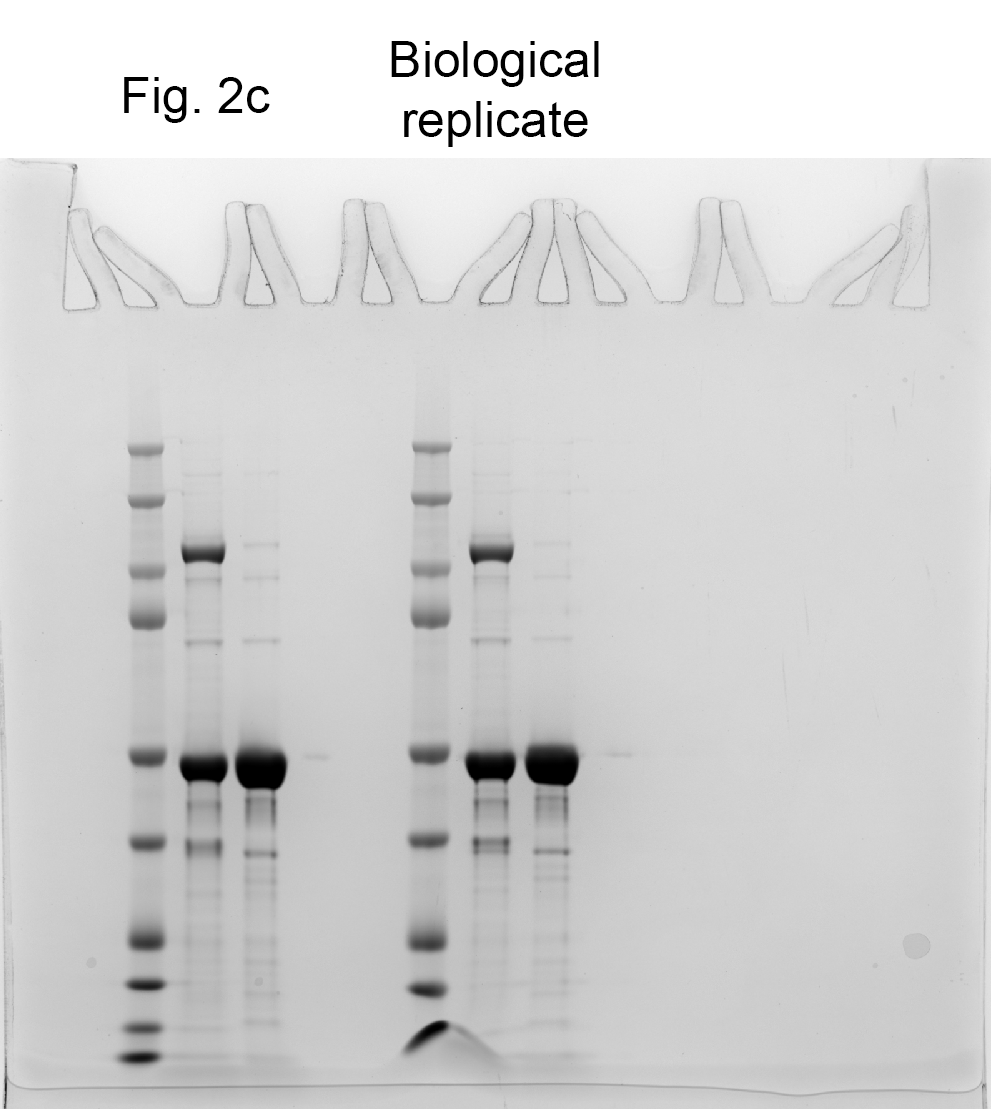

Supplement: Supplementary file 6 — Source data [file 41467_2025_67216_MOESM6_ESM.zip › Feng_etal_Source_Data/Fig2/Fig2c_uncropped.tif]

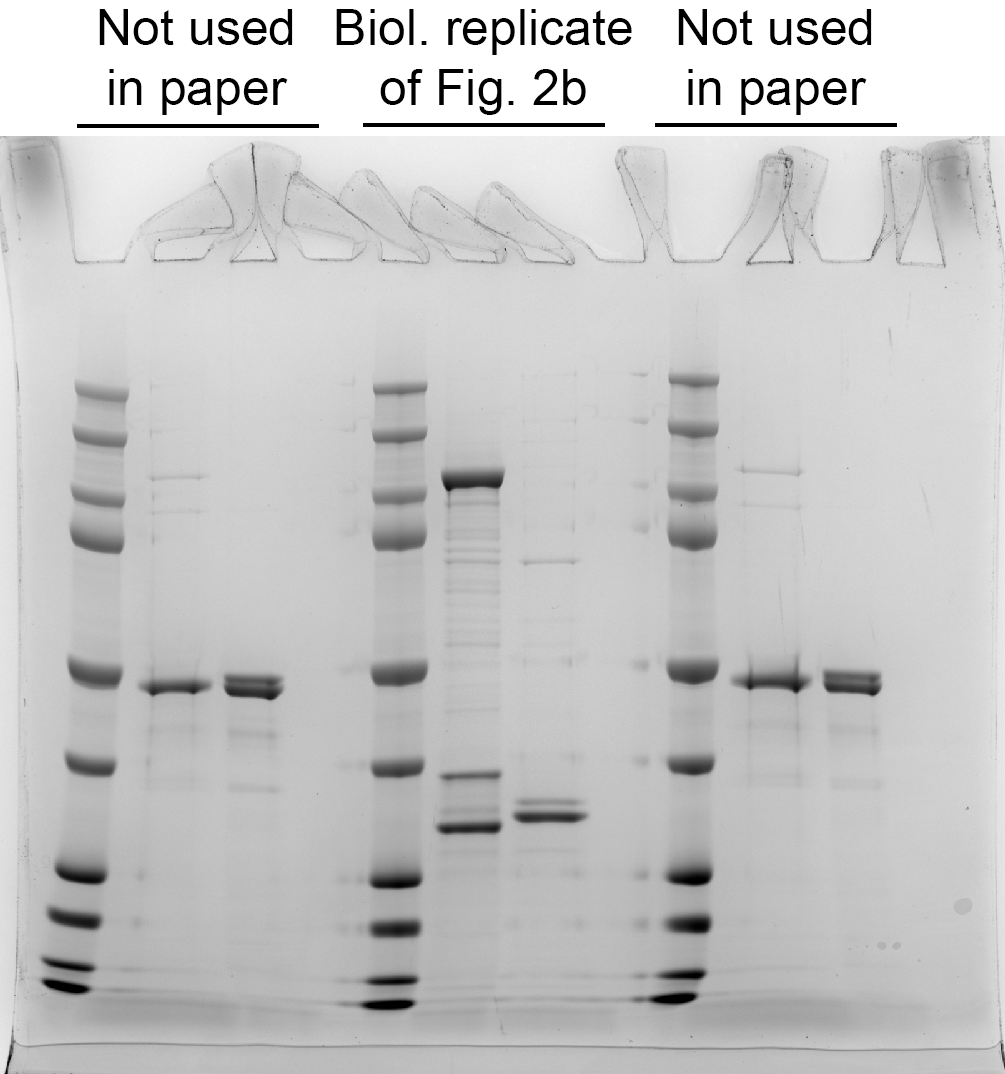

Supplement: Supplementary file 6 — Source data [file 41467_2025_67216_MOESM6_ESM.zip › Feng_etal_Source_Data/Fig2/Fig2b_replicate_uncropped.tif]

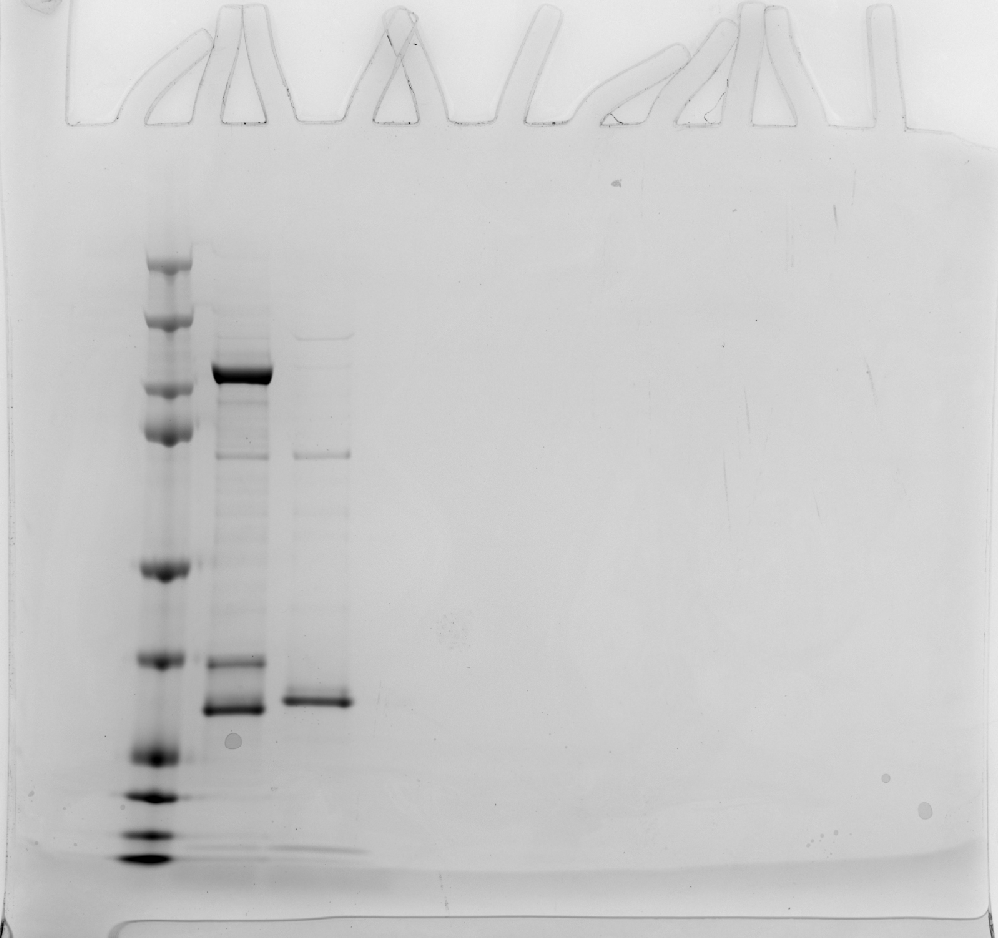

Supplement: Supplementary file 6 — Source data [file 41467_2025_67216_MOESM6_ESM.zip › Feng_etal_Source_Data/Fig2/Fig2b_uncropped.tif]

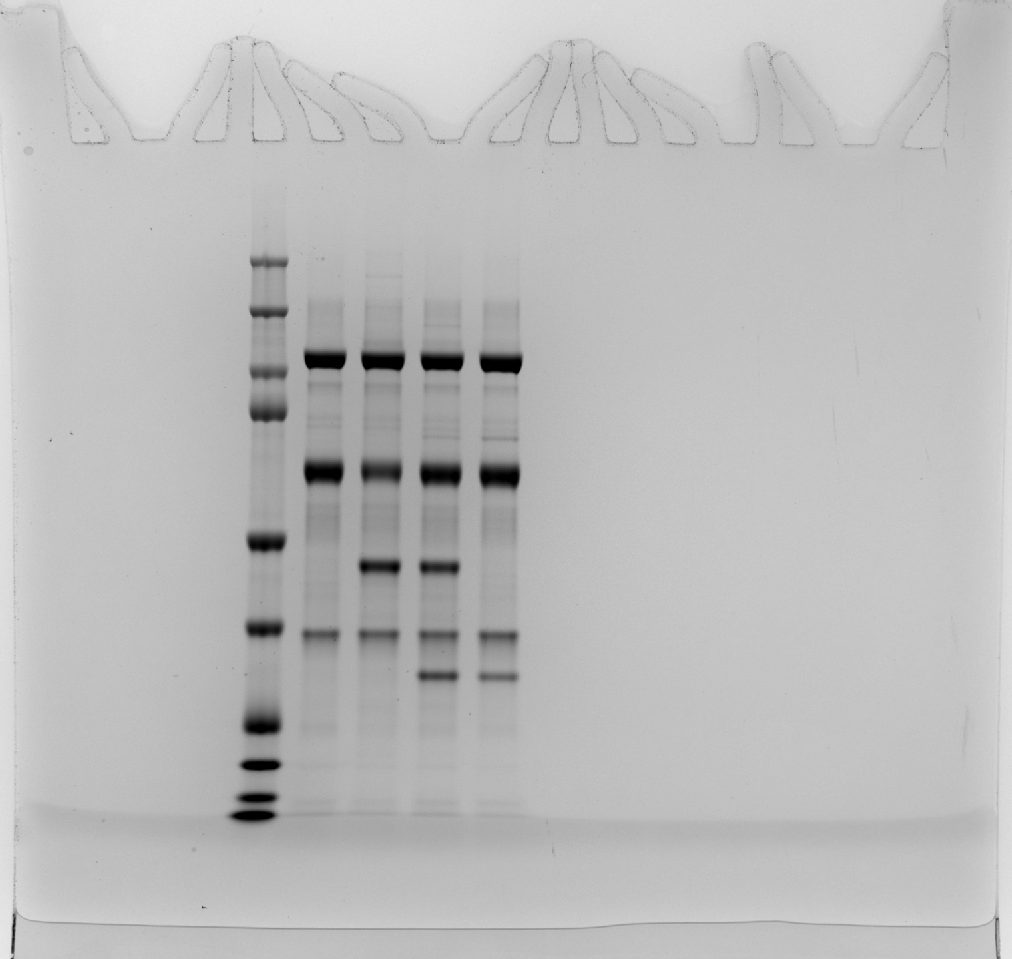

Supplement: Supplementary file 6 — Source data [file 41467_2025_67216_MOESM6_ESM.zip › Feng_etal_Source_Data/Fig3/Fig3c_uncropped.tif]

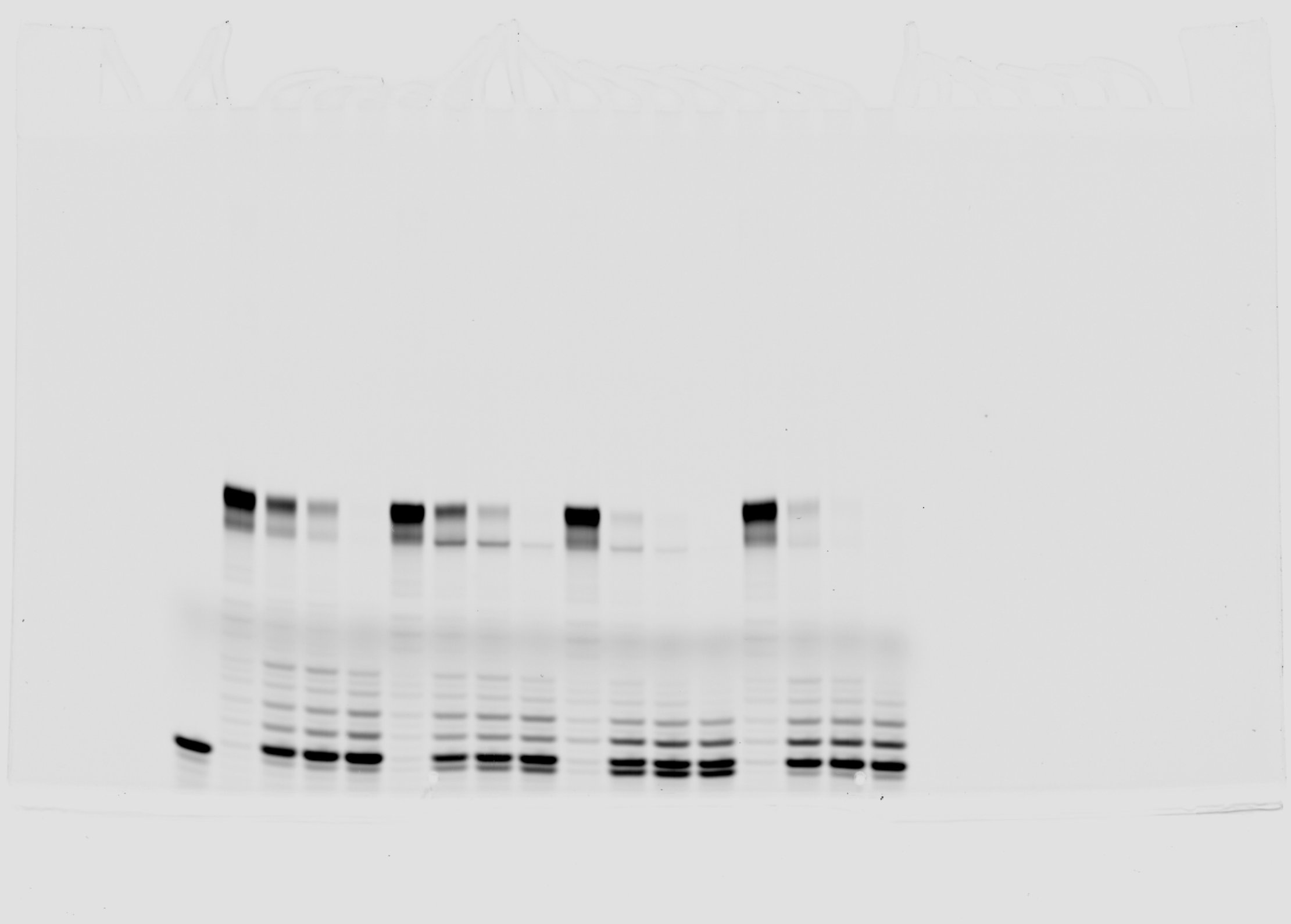

Supplement: Supplementary file 6 — Source data [file 41467_2025_67216_MOESM6_ESM.zip › Feng_etal_Source_Data/Fig3/Fig3b_uncropped.tif]

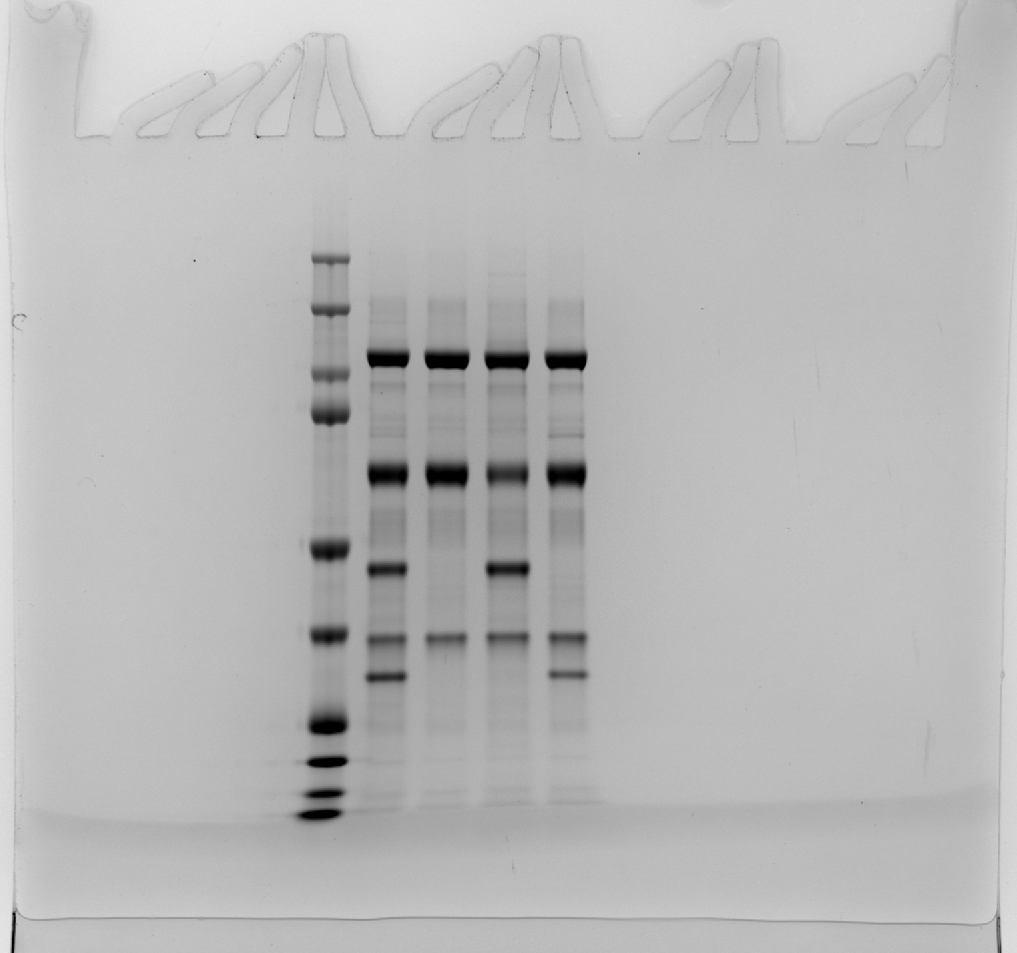

Supplement: Supplementary file 6 — Source data [file 41467_2025_67216_MOESM6_ESM.zip › Feng_etal_Source_Data/SupplementaryFig5/SupFig5d_uncropped.tif]

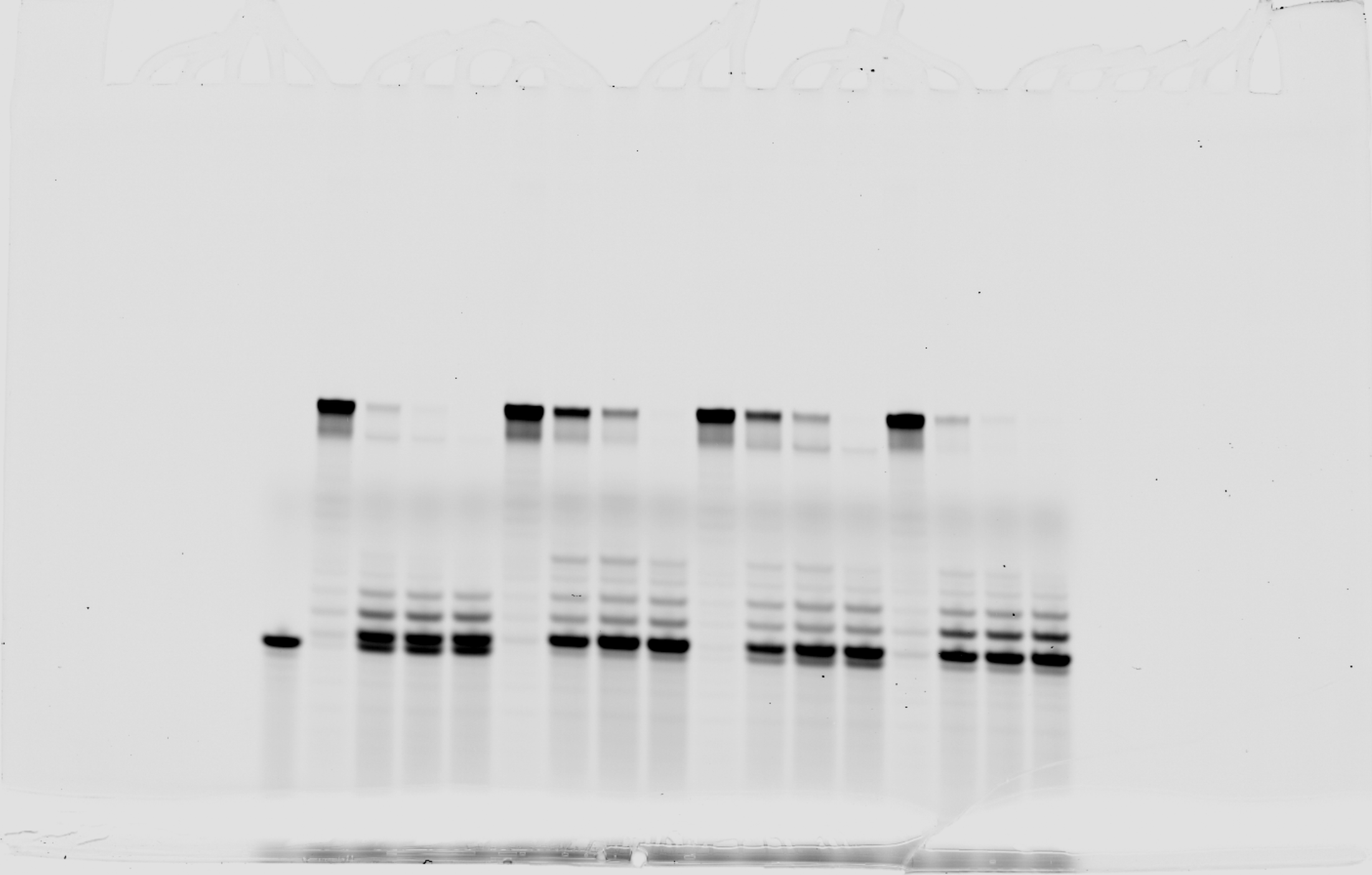

Supplement: Supplementary file 6 — Source data [file 41467_2025_67216_MOESM6_ESM.zip › Feng_etal_Source_Data/SupplementaryFig5/SupFig5c_uncropped.tif]

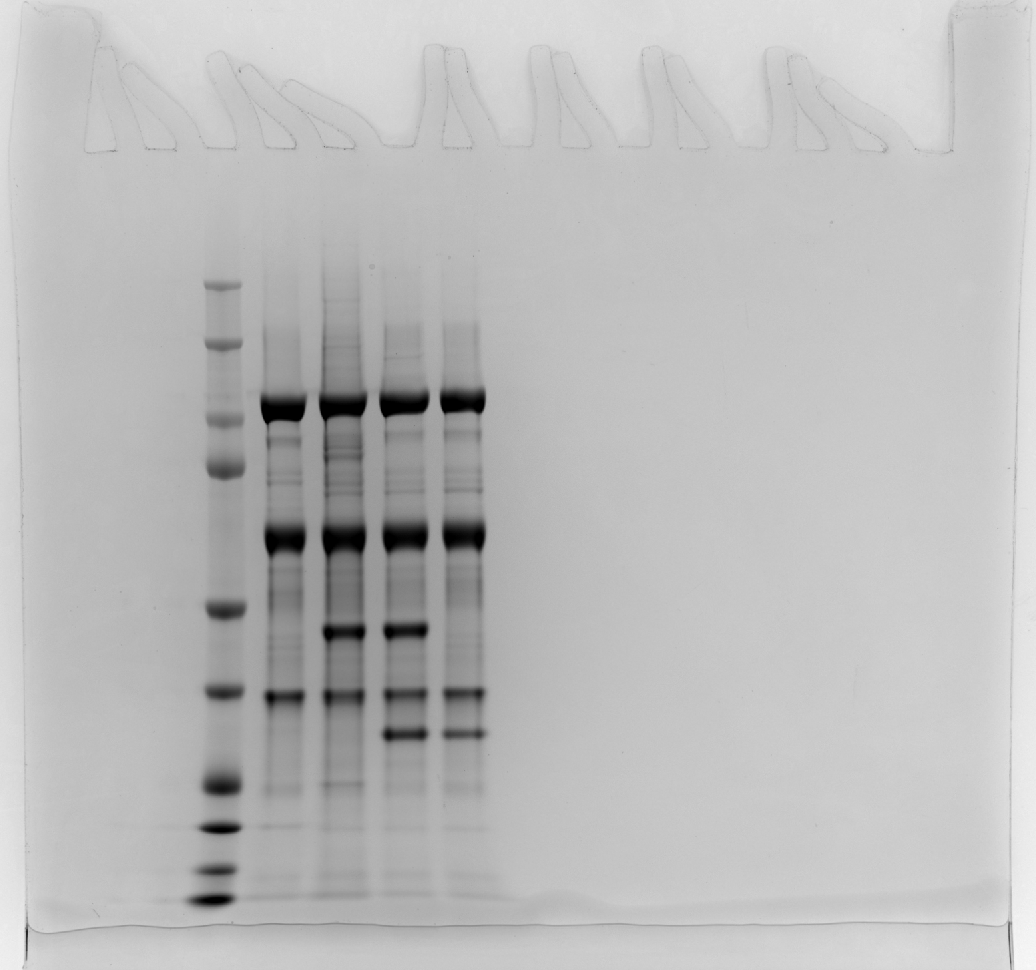

Supplement: Supplementary file 6 — Source data [file 41467_2025_67216_MOESM6_ESM.zip › Feng_etal_Source_Data/SupplementaryFig5/SupFig5b_uncropped.tif]

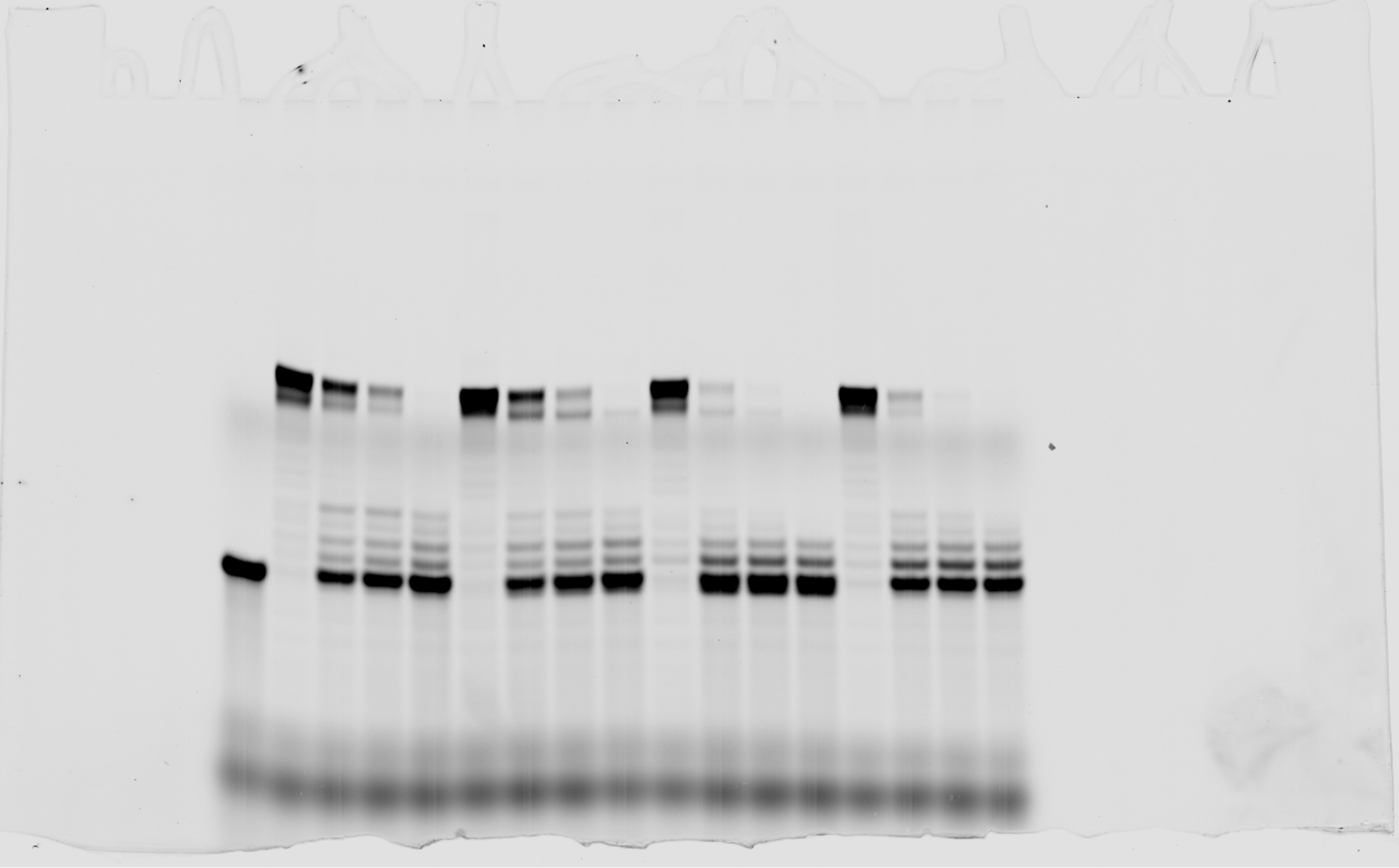

Supplement: Supplementary file 6 — Source data [file 41467_2025_67216_MOESM6_ESM.zip › Feng_etal_Source_Data/SupplementaryFig5/SupFig5a_uncropped.tif]

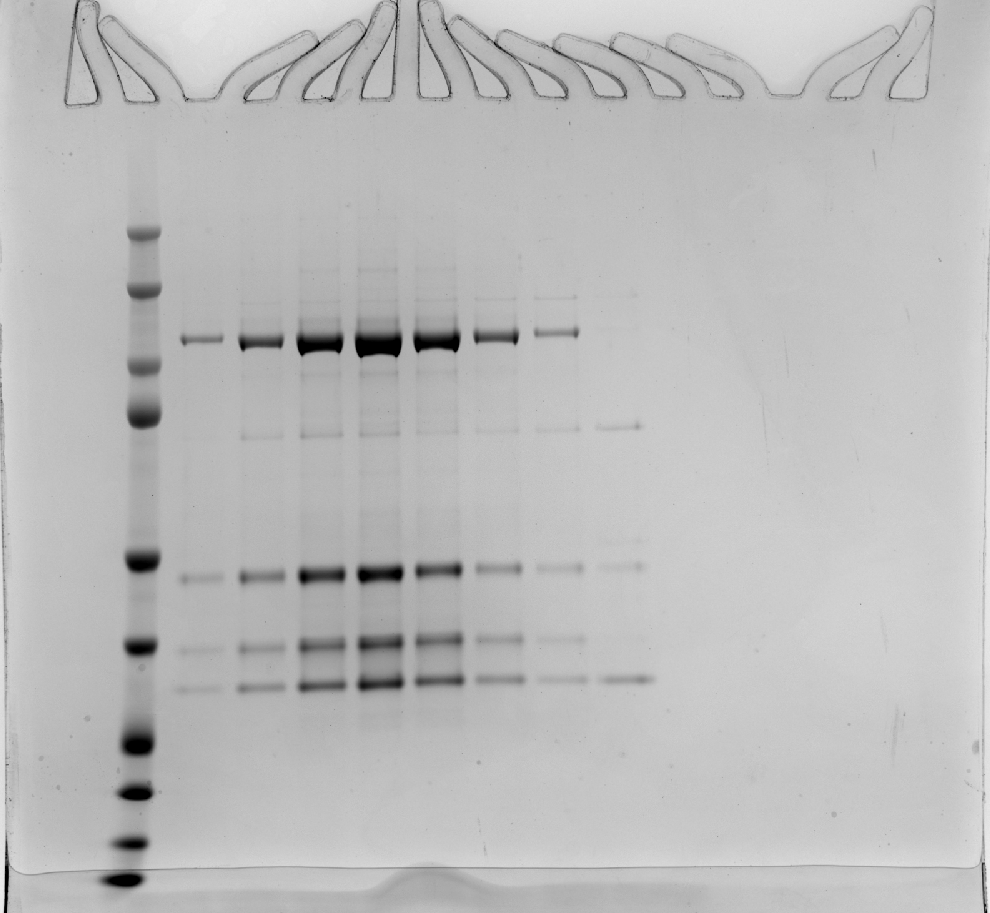

Supplement: Supplementary file 6 — Source data [file 41467_2025_67216_MOESM6_ESM.zip › Feng_etal_Source_Data/SupplementaryFig2/SupFig2d_uncropped.tif]

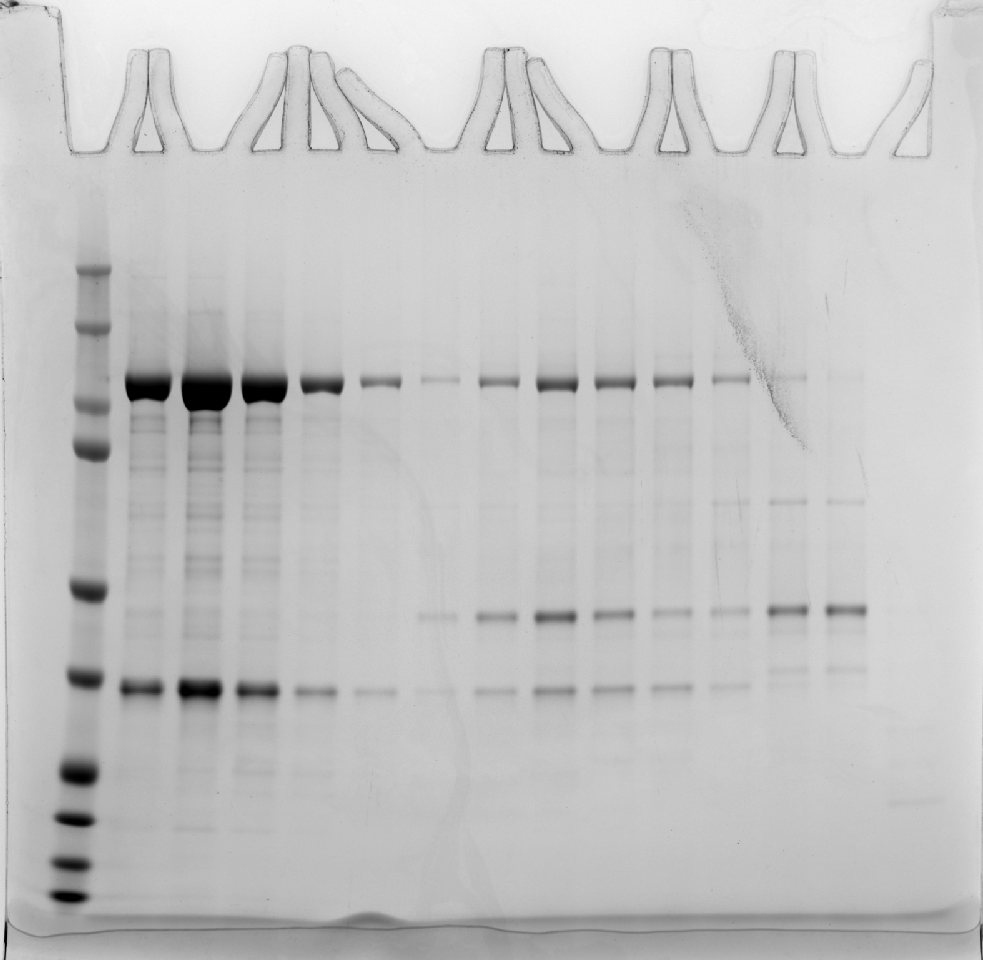

Supplement: Supplementary file 6 — Source data [file 41467_2025_67216_MOESM6_ESM.zip › Feng_etal_Source_Data/SupplementaryFig2/SupFig2c_uncropped.tif]

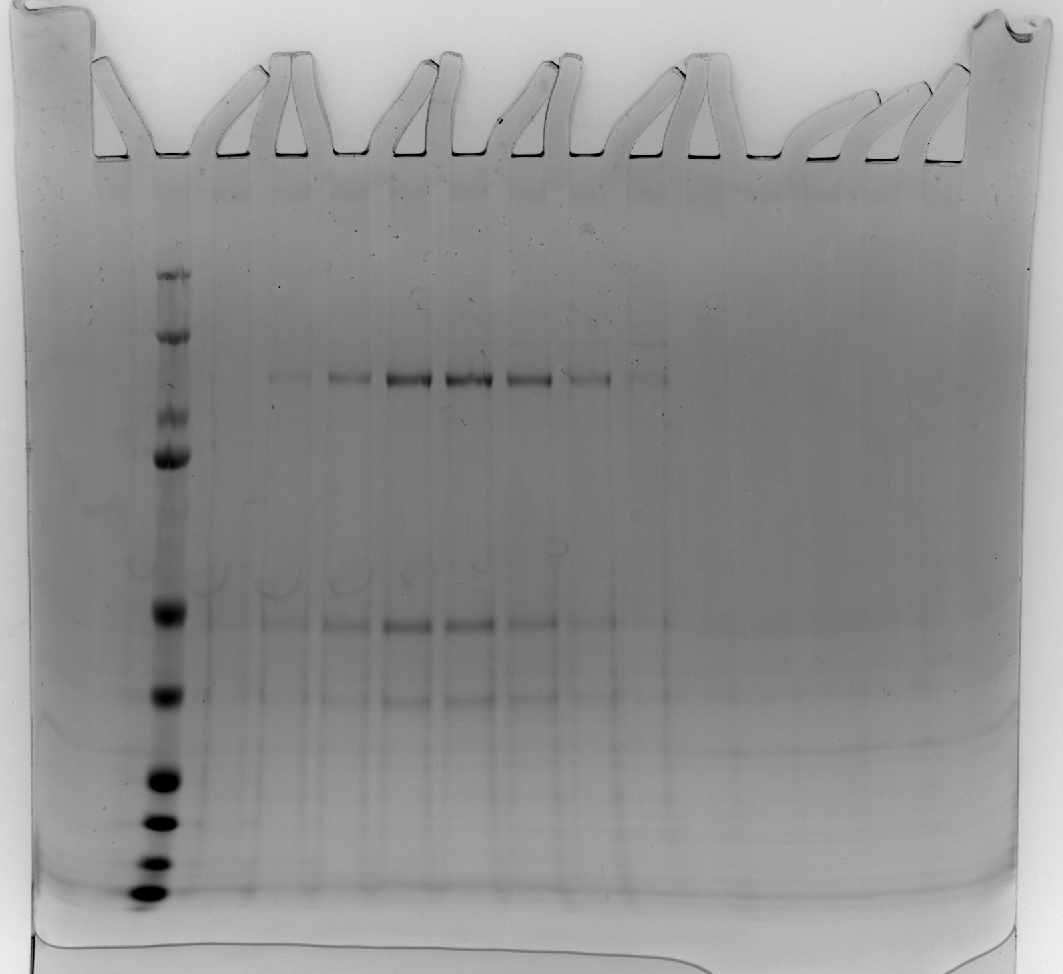

Supplement: Supplementary file 6 — Source data [file 41467_2025_67216_MOESM6_ESM.zip › Feng_etal_Source_Data/SupplementaryFig2/SupFig2g_uncropped.tif]

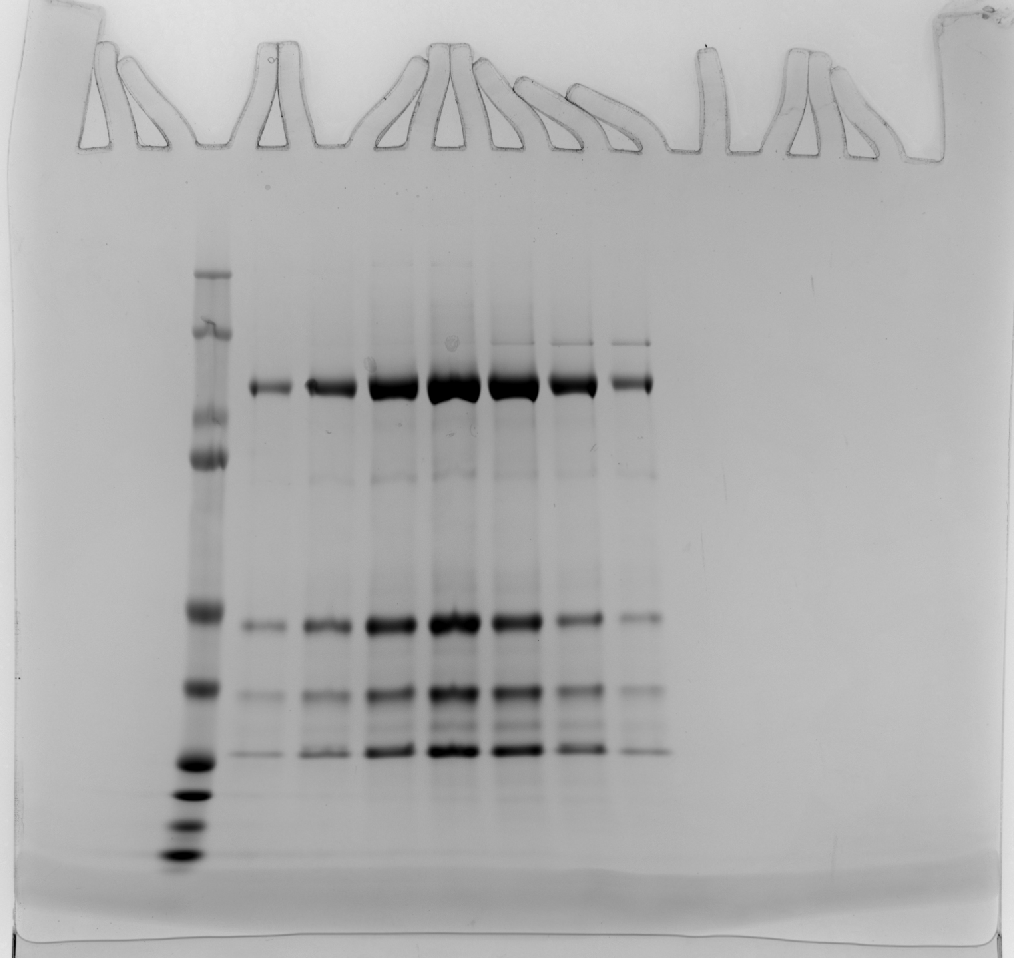

Supplement: Supplementary file 6 — Source data [file 41467_2025_67216_MOESM6_ESM.zip › Feng_etal_Source_Data/SupplementaryFig2/SupFig2i_uncropped.tif]

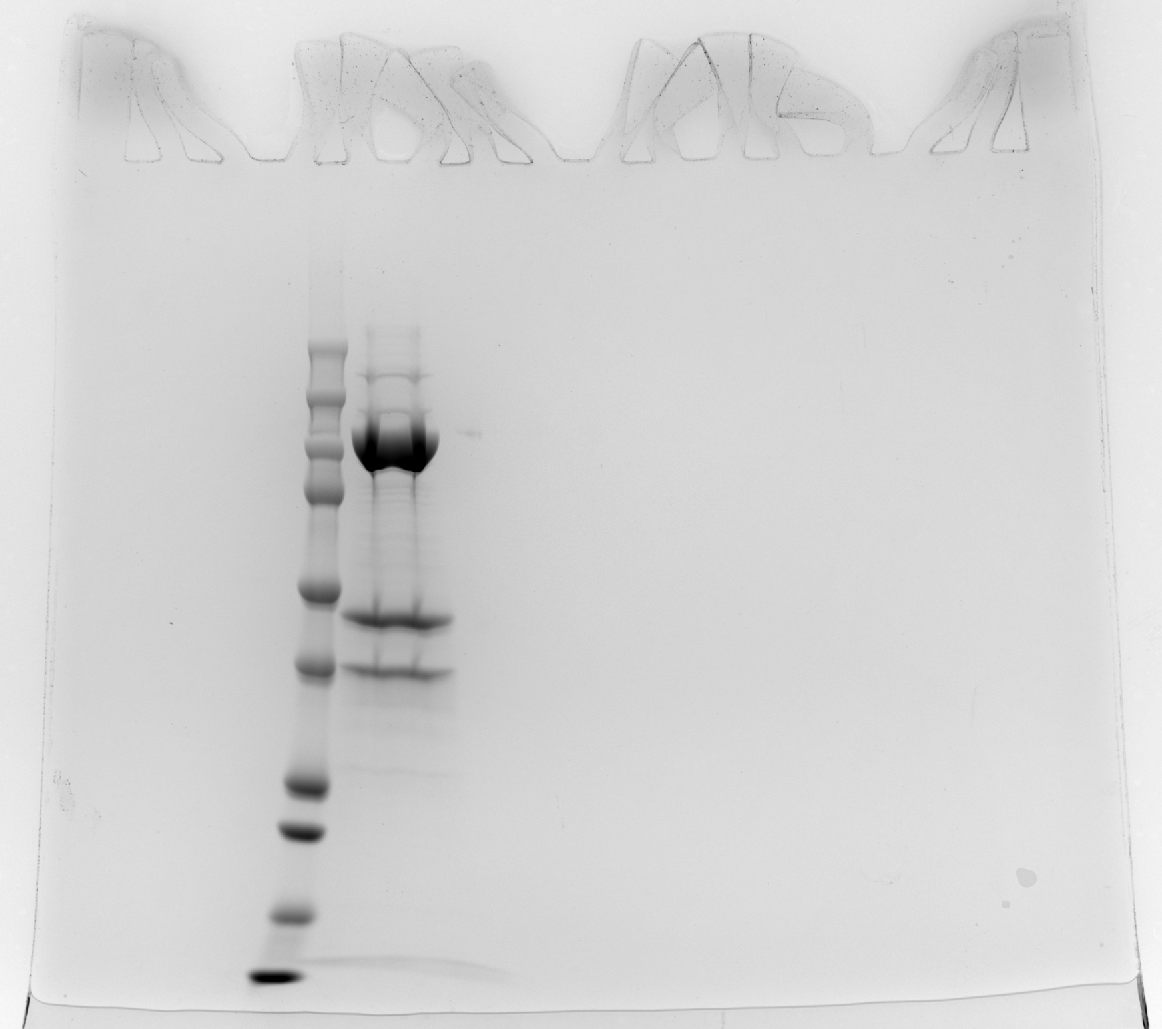

Supplement: Supplementary file 6 — Source data [file 41467_2025_67216_MOESM6_ESM.zip › Feng_etal_Source_Data/SupplementaryFig2/SupFig2h_uncropped.tif]

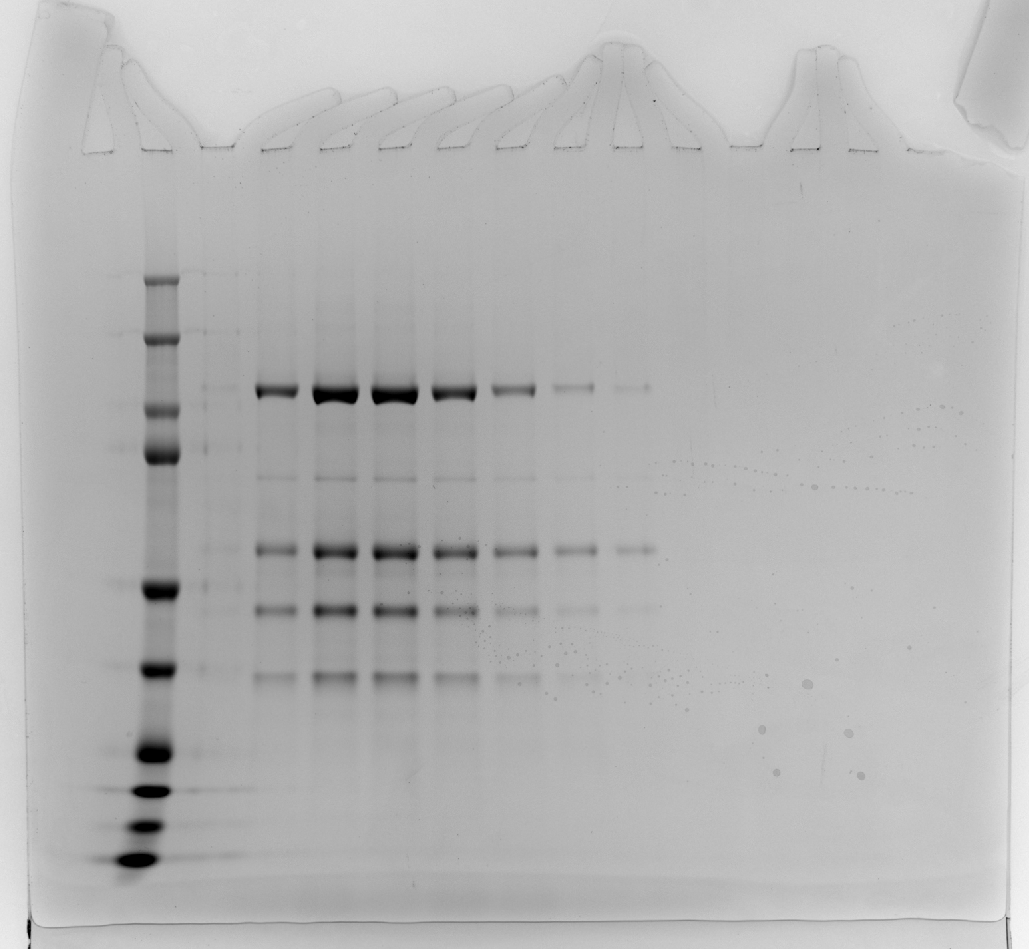

Supplement: Supplementary file 6 — Source data [file 41467_2025_67216_MOESM6_ESM.zip › Feng_etal_Source_Data/SupplementaryFig2/SupFig2f_uncropped.tif]

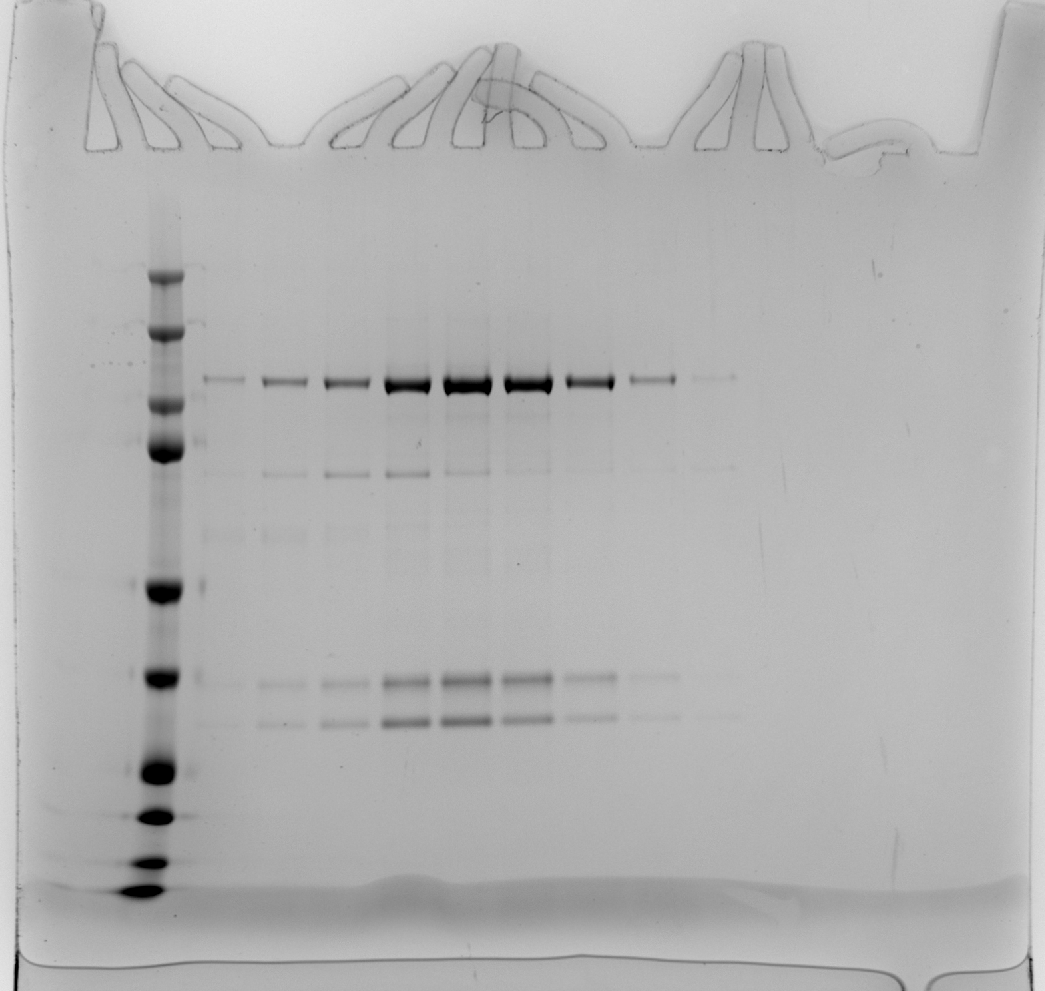

Supplement: Supplementary file 6 — Source data [file 41467_2025_67216_MOESM6_ESM.zip › Feng_etal_Source_Data/SupplementaryFig2/SupFig2b_uncropped.tif]

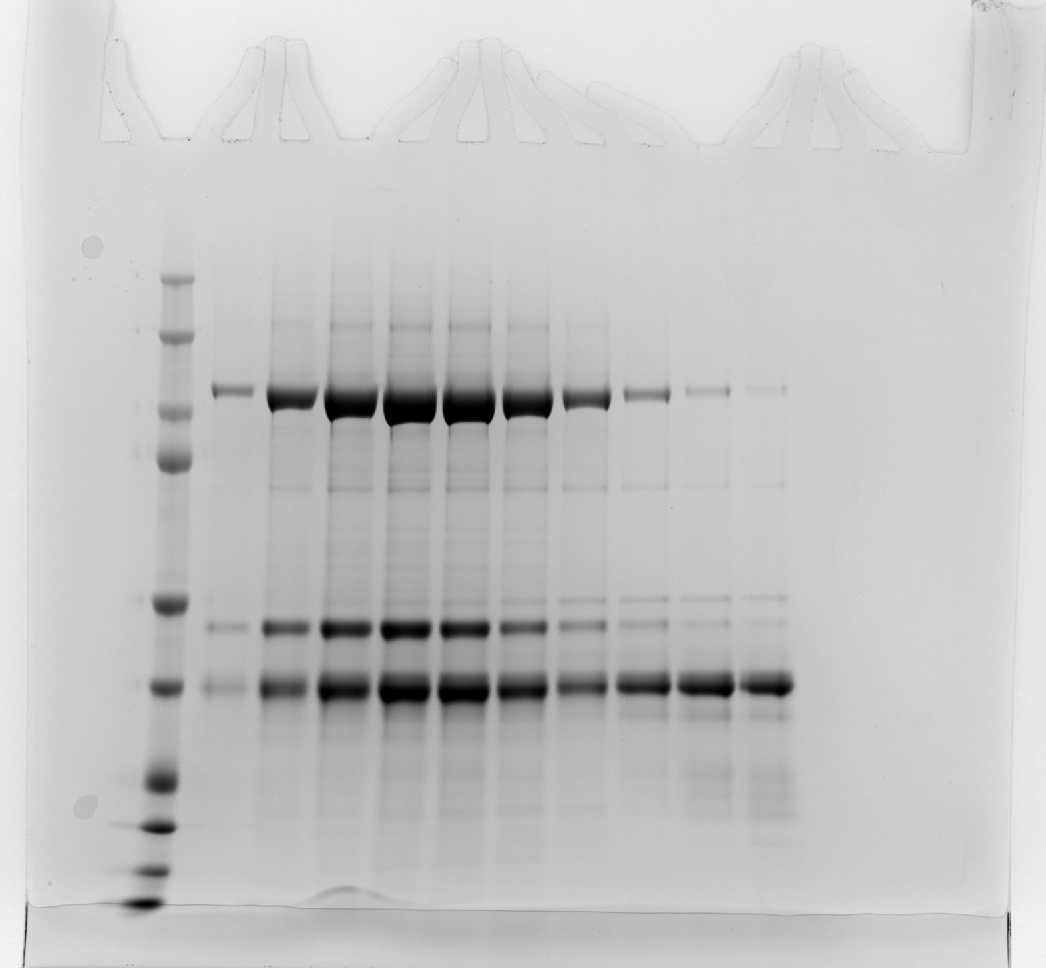

Supplement: Supplementary file 6 — Source data [file 41467_2025_67216_MOESM6_ESM.zip › Feng_etal_Source_Data/SupplementaryFig2/SupFig2e_uncropped.tif]

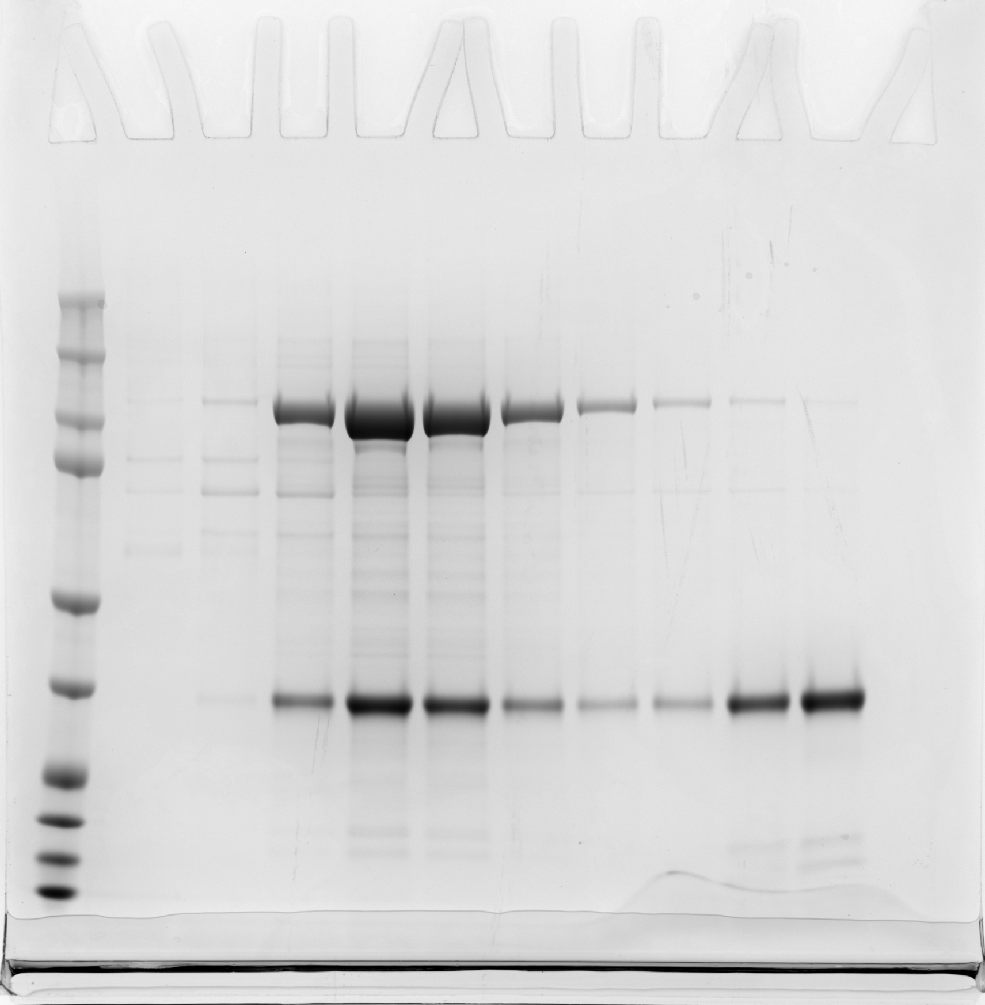

Supplement: Supplementary file 6 — Source data [file 41467_2025_67216_MOESM6_ESM.zip › Feng_etal_Source_Data/SupplementaryFig2/SupFig2a_uncropped.tif]

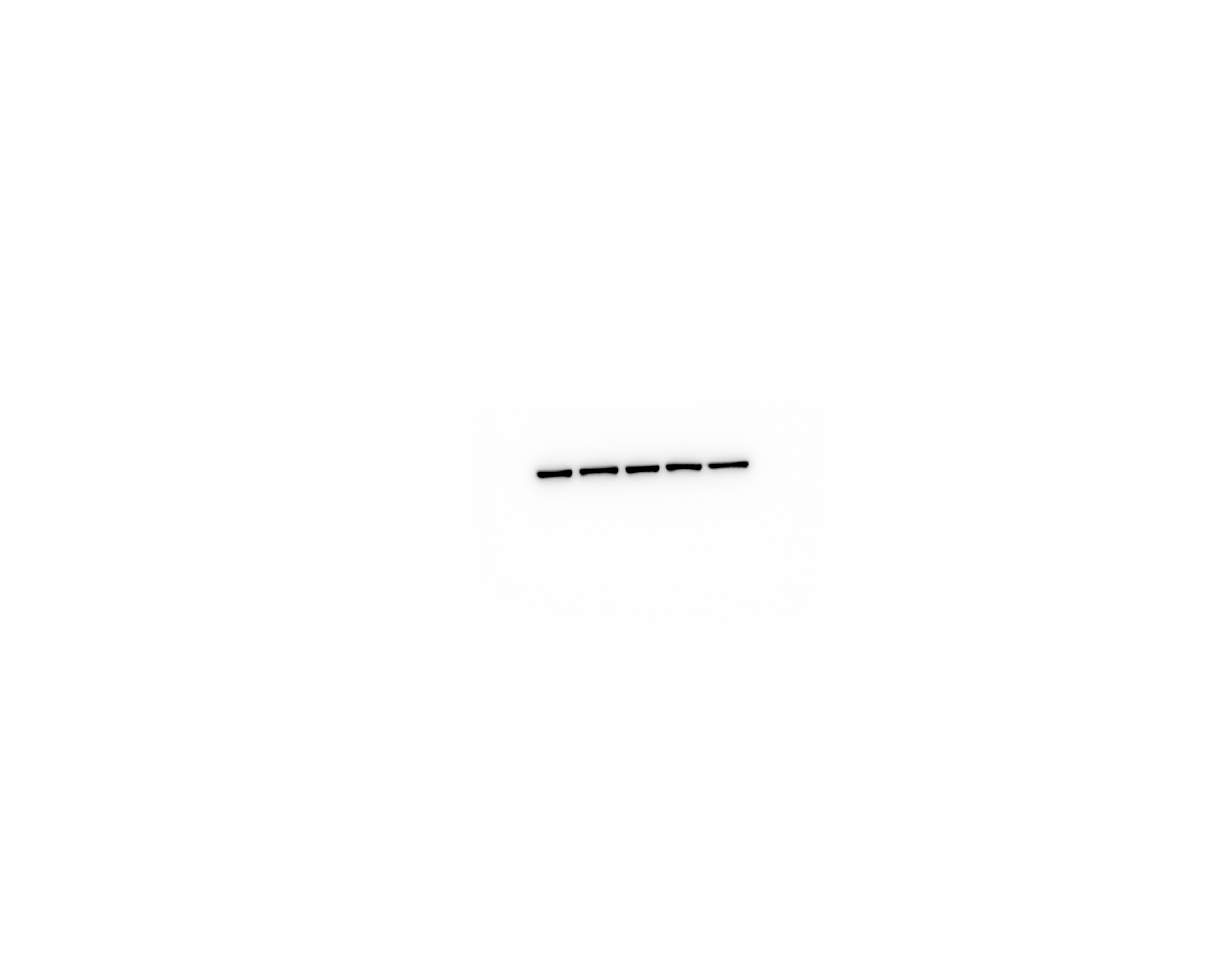

Supplement: Supplementary file 6 — Source data [file 41467_2025_67216_MOESM6_ESM.zip › Feng_etal_Source_Data/SupplementaryFig9/SupFig9d/tubulin_blot.tif]

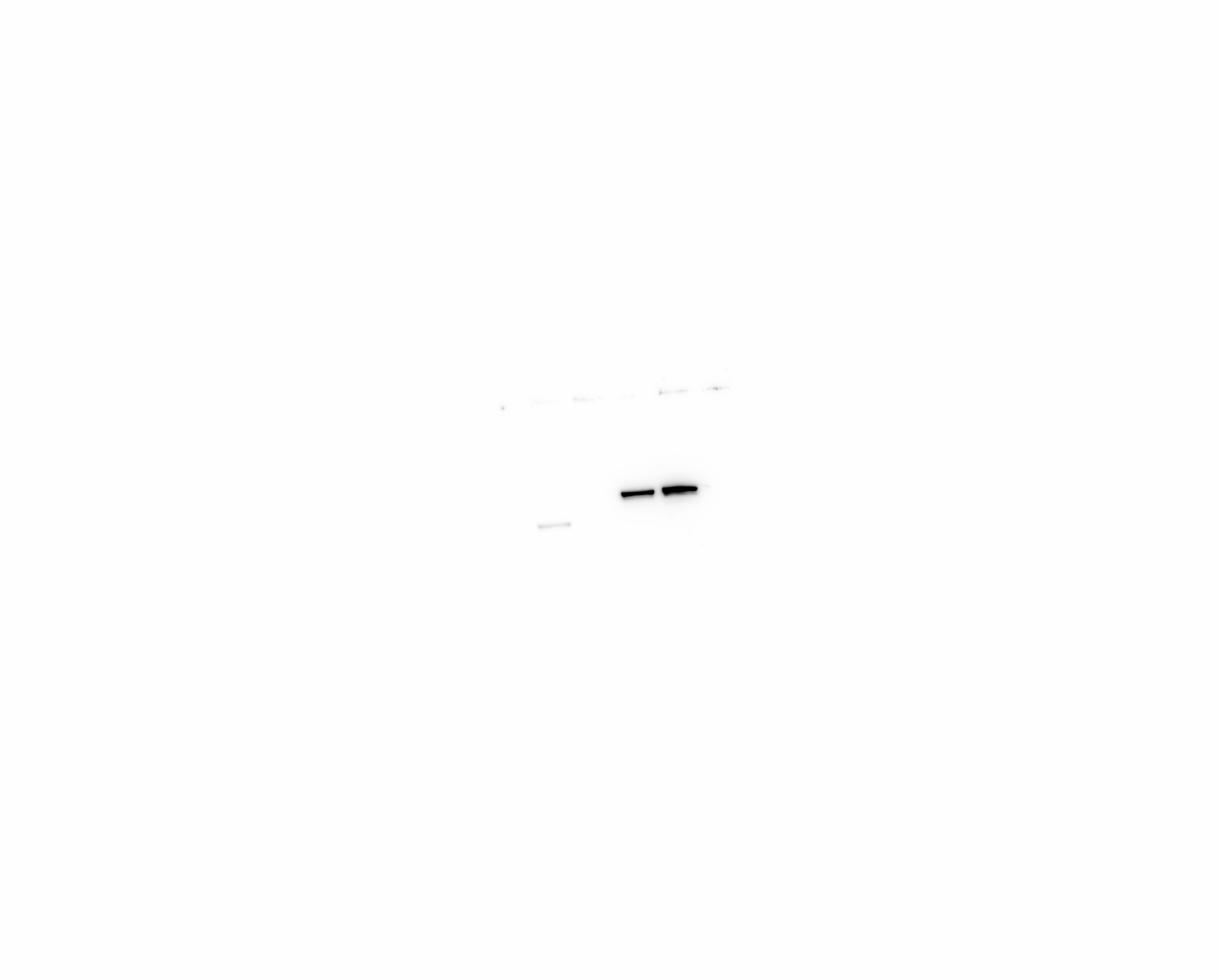

Supplement: Supplementary file 6 — Source data [file 41467_2025_67216_MOESM6_ESM.zip › Feng_etal_Source_Data/SupplementaryFig9/SupFig9d/XPF_blot.tif]

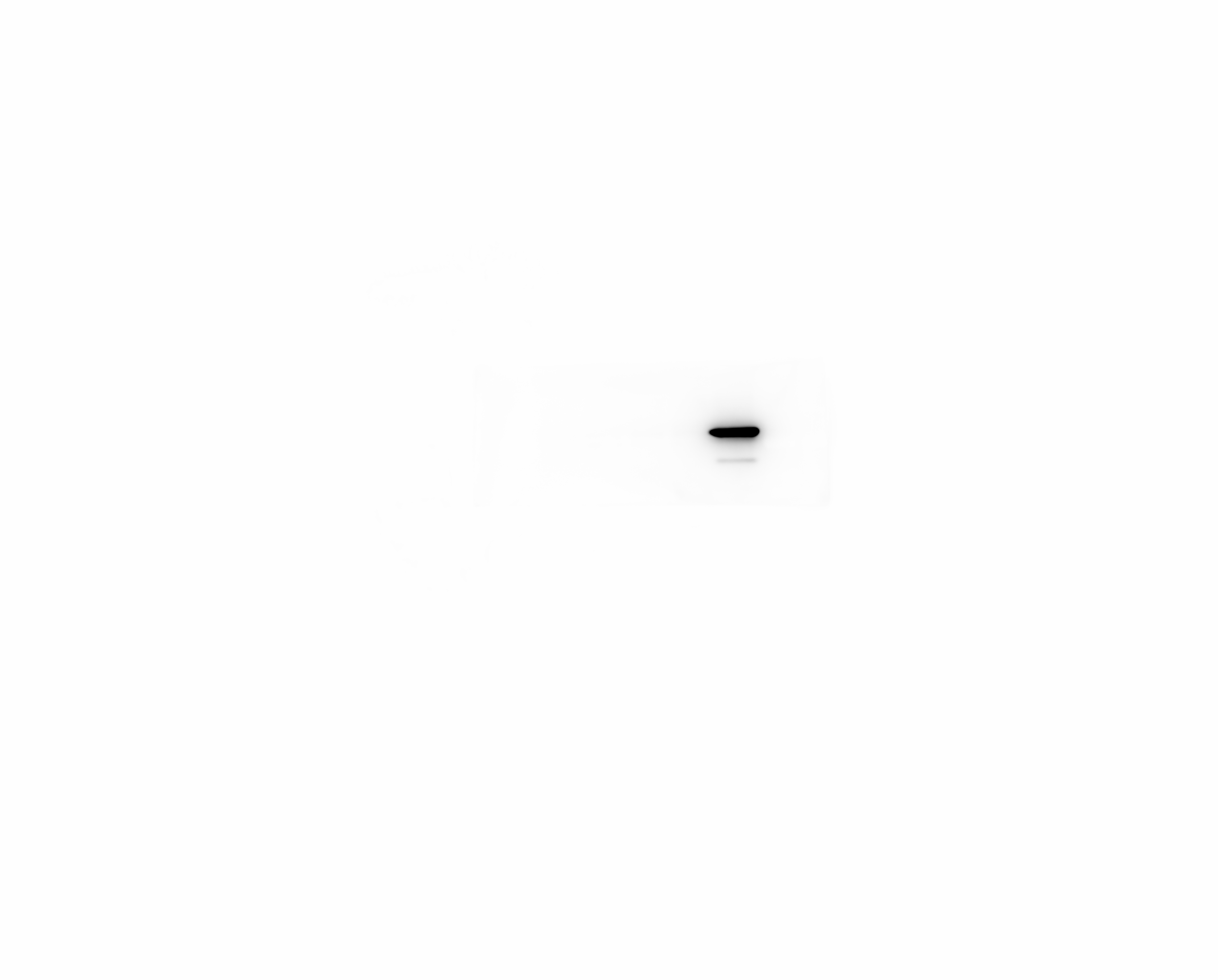

Supplement: Supplementary file 6 — Source data [file 41467_2025_67216_MOESM6_ESM.zip › Feng_etal_Source_Data/SupplementaryFig9/SupFig9d/GFP_blot.tif]

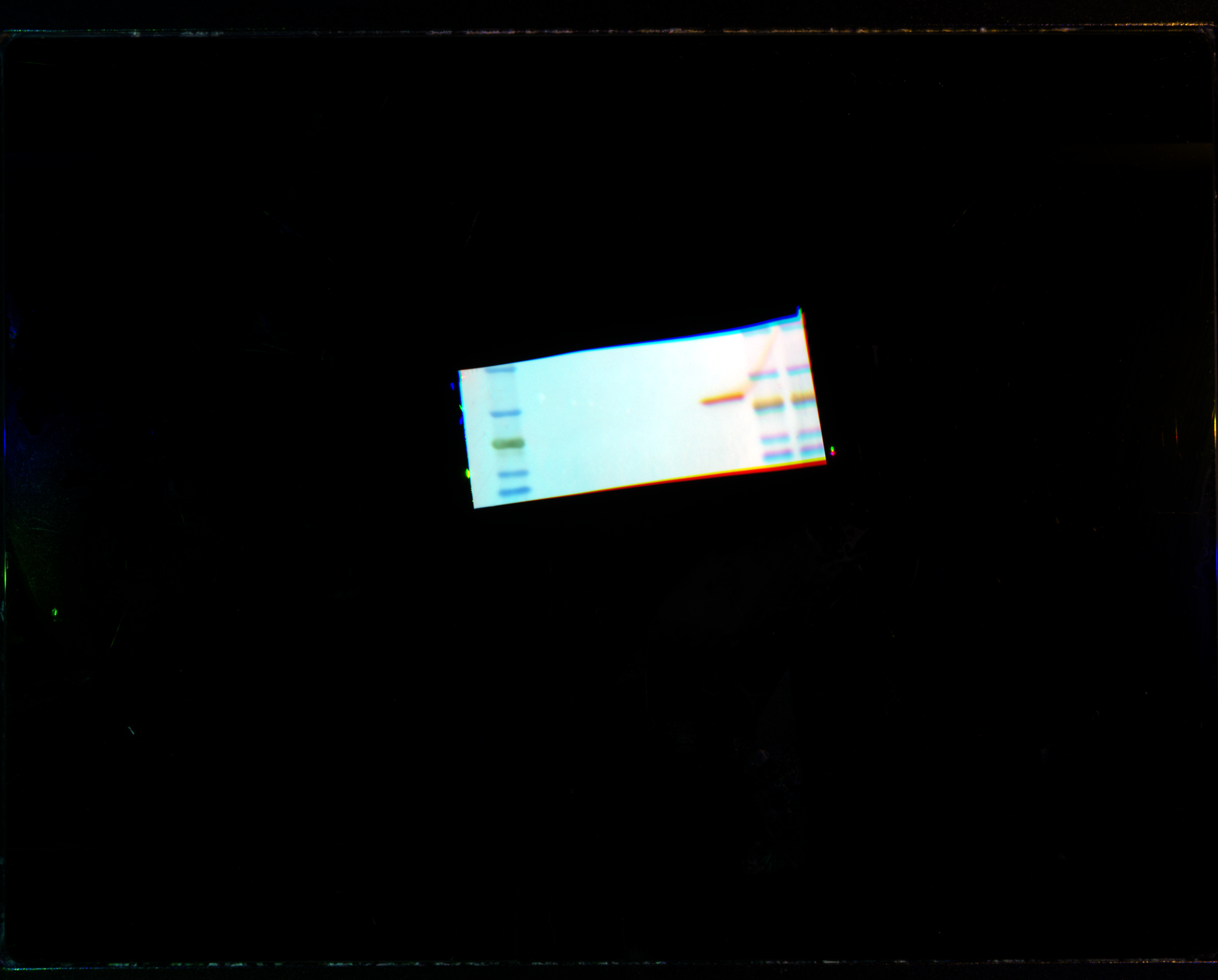

Supplement: Supplementary file 6 — Source data [file 41467_2025_67216_MOESM6_ESM.zip › Feng_etal_Source_Data/SupplementaryFig9/SupFig9d/GFP_marker.tif]

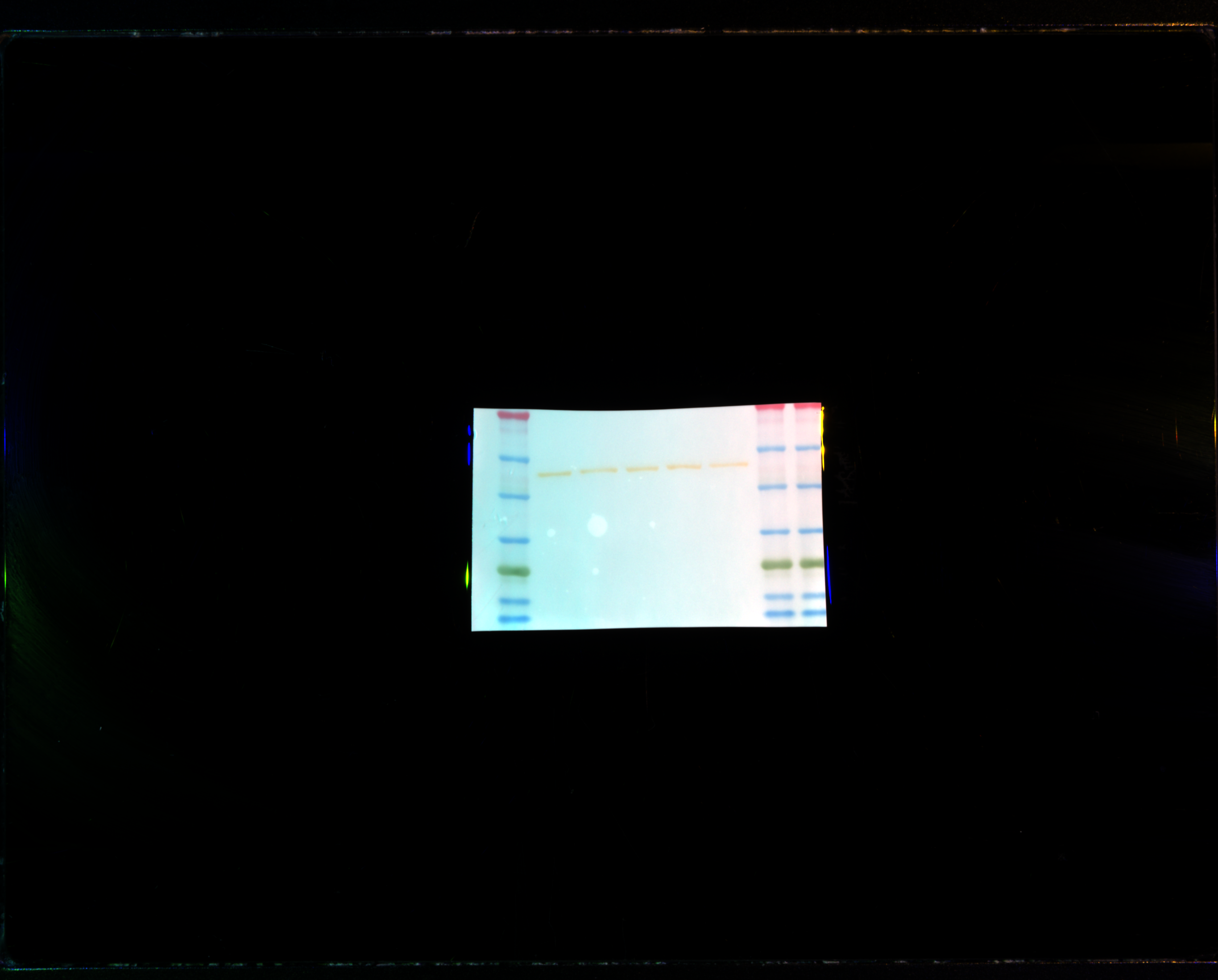

Supplement: Supplementary file 6 — Source data [file 41467_2025_67216_MOESM6_ESM.zip › Feng_etal_Source_Data/SupplementaryFig9/SupFig9d/tubulin_marker.tif]

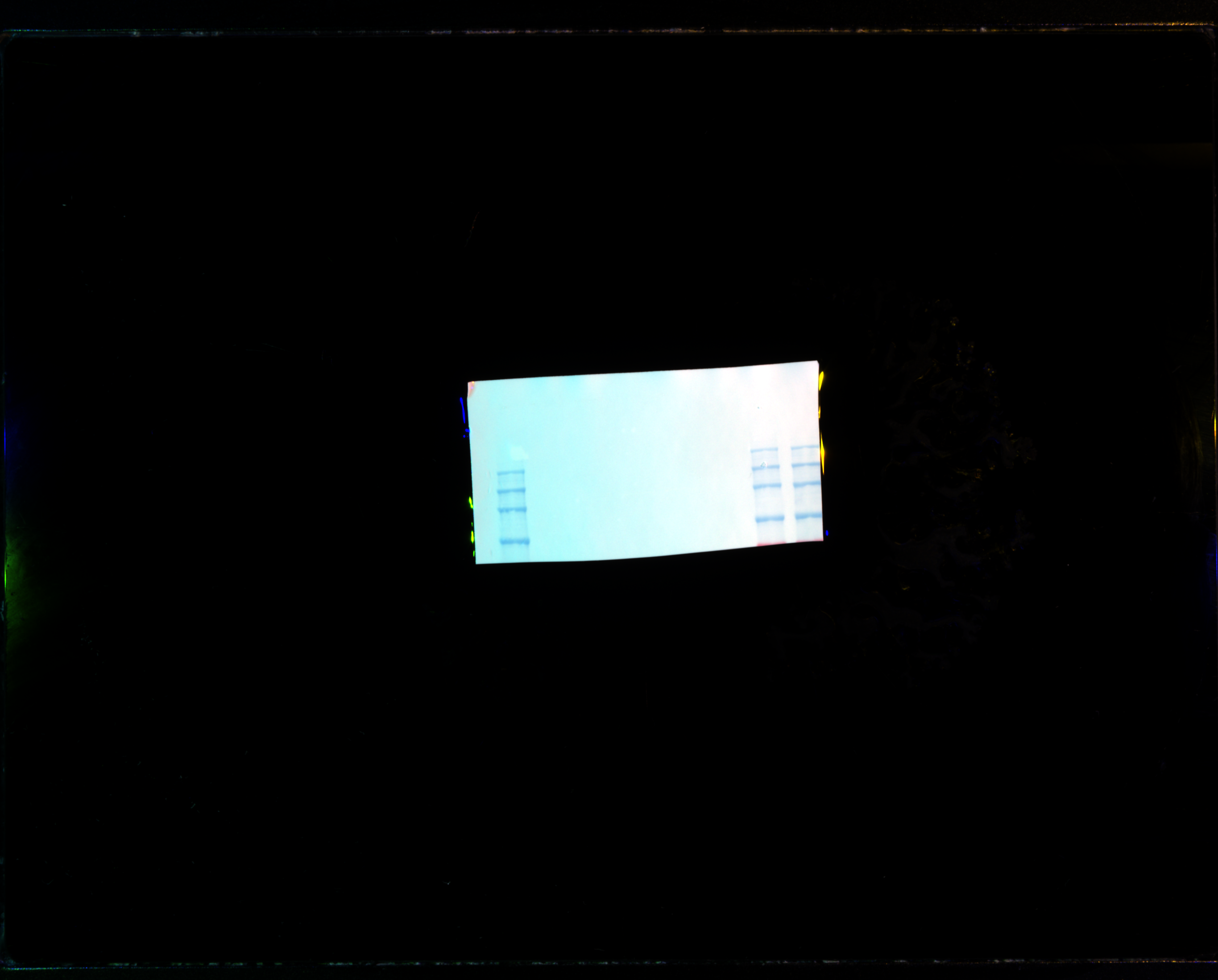

Supplement: Supplementary file 6 — Source data [file 41467_2025_67216_MOESM6_ESM.zip › Feng_etal_Source_Data/SupplementaryFig9/SupFig9d/XPF_marker.tif]

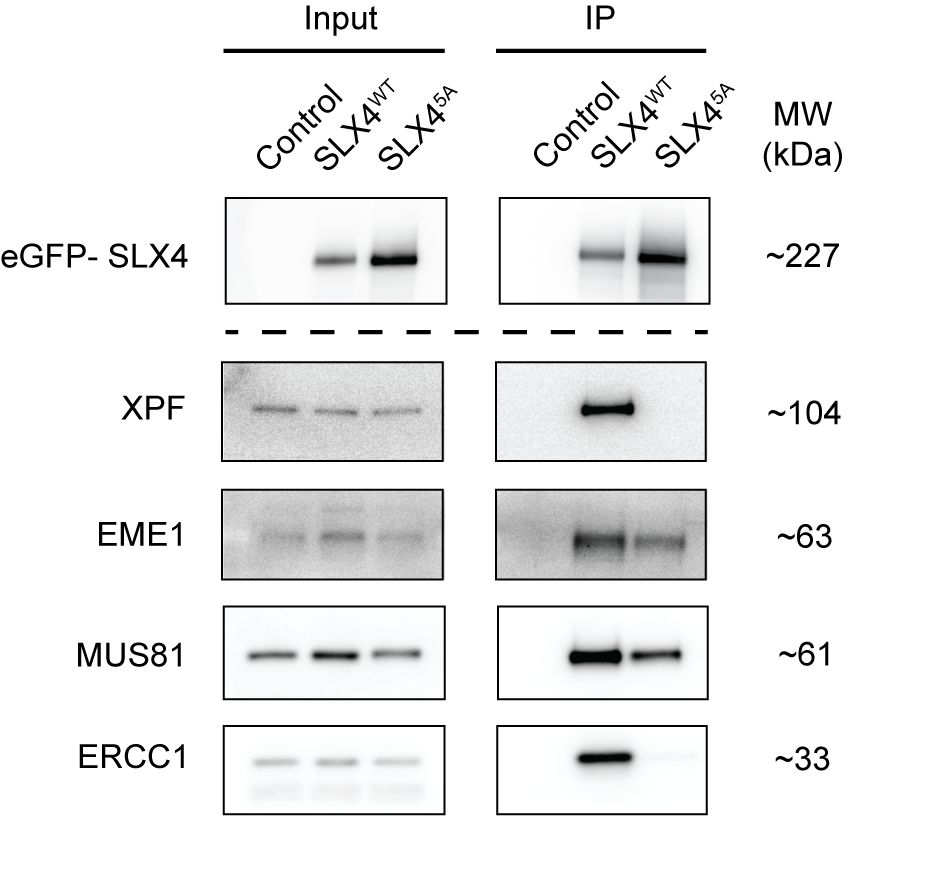

Supplement: Supplementary file 6 — Source data [file 41467_2025_67216_MOESM6_ESM.zip › Feng_etal_Source_Data/Fig2/Fig2d_biological_replicate/Fig2d_replicate.png]

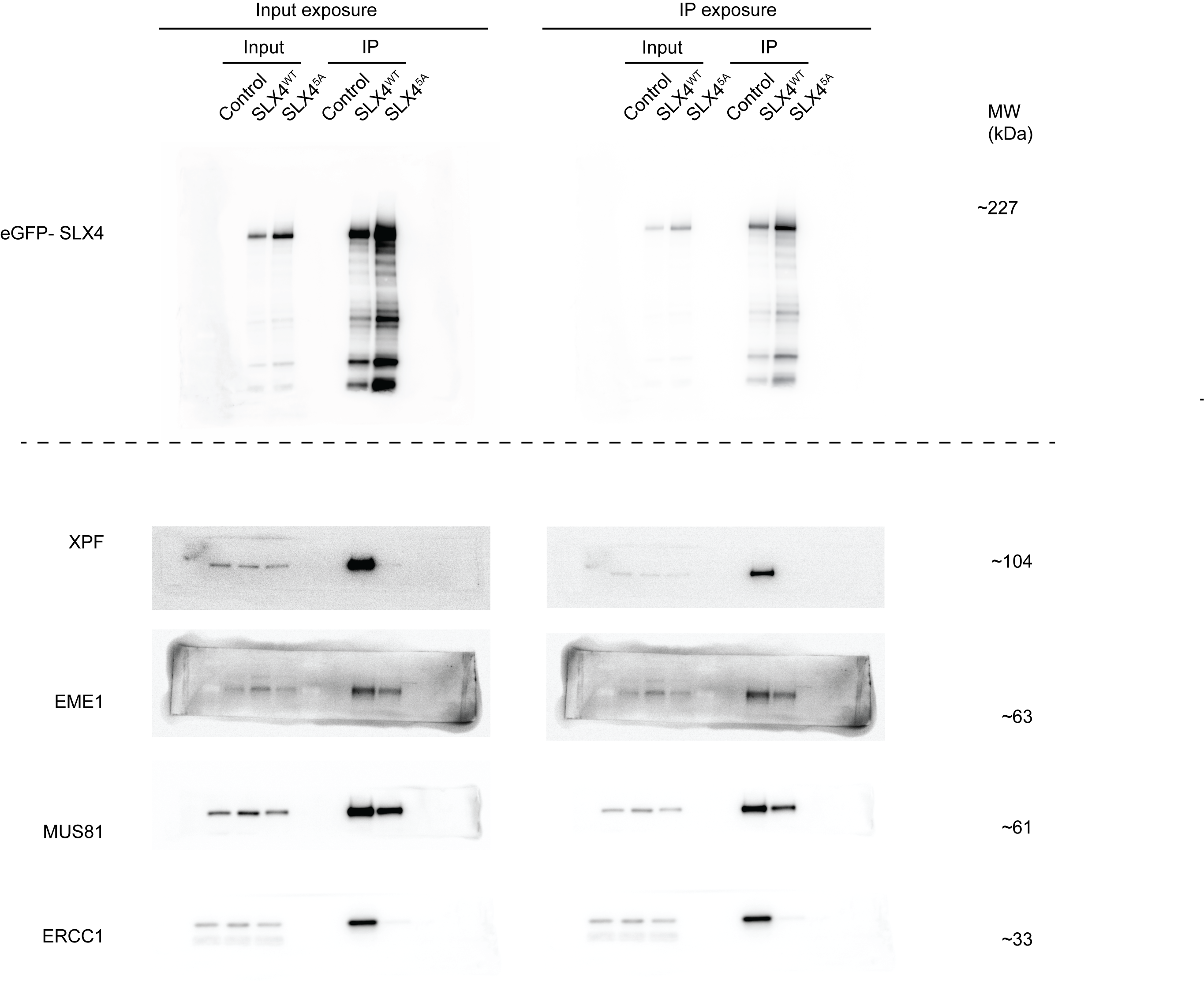

Supplement: Supplementary file 6 — Source data [file 41467_2025_67216_MOESM6_ESM.zip › Feng_etal_Source_Data/Fig2/Fig2d_biological_replicate/Fig2d_replicate_uncropped_membranes.png]
